# Gaps in the global health research landscape for mpox: an analysis of research activities and existing evidence

**DOI:** 10.1186/s12916-025-04350-1

**Published:** 2025-09-29

**Authors:** Rodrigo Furst, Emilia Antonio, Marieke de Swart, Isabel Foster, Johannes Paolo Cerrado, Zaharat Kadri-Alabi, Susan Khader Ibrahim, Lauren Ashley, Duduzile Ndwandwe, Louise Sigfrid, Alice Norton

**Affiliations:** 1 Policy and Practice Research Group, Pandemic Sciences Institute, University of Oxford, Oxford, UK; 2 GloPID-R Research and Policy Team, Policy and Practice Research Group, Pandemic Sciences Institute, University of Oxford, Oxford, UK; 3 Cochrane South Africa, South African Medical Research Council, Cape Town, South Africa; 4 GloPID-R Africa Hub, South African Medical Research Council, Cape Town, South Africa

**Keywords:** Mpox, Research prioritisation, Research funding, Research gaps

## Abstract

**Background:**

Since December 2022, human cases of mpox in the Democratic Republic of the Congo (DRC) have risen at unprecedented rates. We identified a need for systematic mapping of the active research landscape and evidence, assessing their alignment with both local and global research priorities, to inform urgently needed research investments to support response efforts.

**Methods:**

We conducted a mapping review of global research funding and international clinical trial registries and established a systematic rapid research needs appraisals platform to identify existing evidence gaps on mpox research. We analysed the alignment of these to established research categories and both local and globally identified mpox-specific research priorities.

**Results:**

We identified 124 ongoing mpox research grants, 79 registered trials and 415 published studies. Most grants (85.0%, *n* = 105/124), clinical trials (49.3%, *n* = 39/79) and primary studies (57.7%, *n* = 205/355) were conducted in high-income countries, with most evidence published in response to the 2022 clade II global mpox outbreaks. Research funding has been focussed on vaccine and therapeutic pre-clinical development. Key gaps remain in both ongoing research and evidence relating to clinical characterisation among populations at risk, clinical trials on effective medical countermeasures specific to clade I, social sciences, health systems research, and healthcare and community protection.

**Conclusions:**

Our findings highlight persistent research gaps related to mpox clade Ib, particularly the limited knowledge on its characteristics and a lack of ongoing efforts to develop effective medical countermeasures, posing a risk to control efforts. Aligning research and investments to locally and globally identified research priorities and evidence gaps will help support national, regional and international responses to prevent transmission and improve outcomes.

**Supplementary Information:**

The online version contains supplementary material available at 10.1186/s12916-025-04350-1.

## Background

Mpox is a zoonotic disease caused by the monkeypox virus, belonging to the *Orthopoxvirus* genus [1]. Two main clades, I and II, are known to cause human infections, with clade II typically resulting in a self-limiting disease, even though both clades can lead to severe complications, particularly in children and immunocompromised individuals [1].

The first human case of mpox was identified in the Democratic Republic of the Congo (DRC) in 1970 [1]. Until 2022, mpox remained endemic in 11 West and Central African countries [2]. In 2017, Nigeria experienced a significant clade II outbreak after nearly four decades without reported cases [3]. Several other West African nations also reported re-emergent outbreaks between 2010 and 2019 [3]. In March 2022, a global mpox outbreak affecting previously non-endemic countries was reported for the first time [4]. Unlike earlier outbreaks, this clade II outbreak primarily spread through sexual contact, predominantly affecting men who have sex with men (MSM) [5].

Since December 2022, the DRC has faced the largest mpox outbreak to date, with over 35,000 reported cases and 1000 deaths (as of October 20, 2024) [6]. This outbreak, driven by clade I and a divergent lineage now classified as clade Ib, began in South Kivu Province in April 2024 [7]. Clade Ib primarily spread through heterosexual contact, affecting female sex workers and their communities [8]. It has since extended to neighbouring provinces, including North Kivu—home to large internally displaced populations—and crossed borders into Burundi, Kenya, Rwanda and Uganda [6, 9]. The World Health Organization (WHO) declared a Public Health Emergency of International Concern in August 2024 due to the escalating situation [10].

Between 1 January 2024 and 16 March 2025, 23 African countries had reported mpox outbreaks involving clades I and II [6]. Imported cases of the newly emergent clade Ib have also been reported in over 10 non-African countries [6]. The resurgence of mpox in Africa may be linked to increased human-rodent interactions and declining smallpox vaccination immunity [11, 12].

Regional efforts by the Africa CDC, African states and partners, along with the WHO R&D Blueprint through their Collaborative Open Research Consortium (CORC) initiative, have sought to prioritise research through targeted agendas that address critical knowledge gaps, particularly those relating to medical countermeasures [13–15]. The WHO has emphasised the need for a coordinated research response to contain mpox outbreaks and reduce human-to-human transmission [16]. Avoiding duplication and ensuring alignment with established priorities also requires coordination at the research level [17]. Visibility across ongoing research activities and evidence is crucial for identifying gaps and supporting effective decision-making [18].

The Pandemic PACT (Pandemic Preparedness: Analytical Capacity and Funding Tracking) programme supports decision-makers by monitoring global funding and evidence for research on diseases with pandemic potential [19]. Here, we present an analysis of existing mpox research activities and evidence against the local research priorities identified by African countries and global priorities identified by WHO to support decision-making locally, regionally and globally through gap identification.

## Methods

### Research funding tracking

Pandemic PACT research grant data was collected either by direct data provision by funders or by direct scraping from funder websites (using disease-specific keywords and acronyms as search terms). For mpox, search terms included: ‘*Mpox*’, ‘*monkeypox*’, ‘*MPV*’, ‘*MPXV*’ and ‘*hMPXV*’. For the detailed search protocol, inclusion criteria and data sources, see Seminog et al. [20].

All collected data were screened by a team of trained researchers. Grants from 2020 onwards were included. Data was coded using a combination of manual annotation and machine-learning-assisted approaches [21] against a research categorisation framework comprising 12 broad research areas, each with corresponding sub-categories [20] (a detailed description of the scope of each sub-category within the broader research areas is provided in Additional file 1: Table S1). Information on research location was also gathered through a combination of manual review and automated methods, including web scraping from funders’ websites. In cases where the research location was not explicitly stated in the available grant documentation, the location of the research institution receiving the funds was used.

A standardised training process for data coders includes instructional materials and collaborative meetings to ensure consistency and resolve discrepancies. All data underwent peer review by at least two coders, ensuring independent scrutiny with disagreements resolved through group discussion. For the analysis presented in this paper, data was exported from the Pandemic PACT database on 18 October 2024.

### Clinical trials registries

We searched the Pan African Clinical Trial Registry (PACTR), South African National Clinical Trial Register (SANCTR) and the WHO International Clinical Trials Registry Platform (ICTRP) on 21 October 2024 using the following search terms: ‘*mpox*’, ‘*monkey pox*’, ‘*monkeypox*’, ‘*hMPXV*’, ‘*MPXV*’. PACTR collects data on clinical trials conducted across the African continent in real-time and submits these records to the WHO ICTRP for collation, while SANCTR focus on clinical trials conducted in South Africa.

The primary purpose of collecting registry data from this range of sources is to ensure that the dataset is as comprehensive and timely as possible for understanding ongoing research activities. Only studies with a start date from January 2020 onwards were included. The studies identified were screened by the study title, project description, and primary and secondary outcome (where available) for their relevance to mpox using Microsoft Excel.

We classified the studies as interventional or observational and reviewed their recruitment status (i.e. whether actively recruiting or not). For vaccine and therapeutic trials, we extracted the names of the interventions. The locations of the study sites and primary sponsors were also reviewed.

### Rapid research needs appraisal (RRNA)

The RRNA protocol was developed to rapidly and robustly identify existing evidence across seven pre-defined domains (1. Clinical characteristics; 2. Transmission; 3. Pre- and post-prophylaxis; 4. Diagnostics; 5. Treatments, 6. Supportive care; 7. Social sciences) and gaps across demographics and other risk factors for emerging infectious diseases [22].

It is developed in a modular approach to enable targeting of specific domains depending on need. The RRNA utilises a global relay system and review software for efficiency, effectiveness and quality. Data are extracted for each domain by level of study design (e.g. where a systematic review [SR] covers the questions within a domain, we do not extract data from primary articles for that domain). We activated our RRNA protocol in response to the mpox outbreak in DRC on 24 February 2024.

We initially searched for SRs in Ovid Embase, Scopus, Epistemonikos and Cochrane Library from 1 January 1970 to 24 February 2024 to inform targeting of the search for primary studies. We included studies focussed on humans or human samples and at least one of the questions in our protocol. We excluded case studies and case reports with fewer than 25 cases, conference abstracts, pre-prints, narrative and scoping reviews. There were no language restrictions.

We identified a high number of SRs (*n* = 60). Most (*n* = 32) focussed on clinical characteristics (domain 1) and transmission (*n* = 14) (domain 2), including articles published up to December 2023. For the other domains, we identified SRs including studies up to December 2021. We therefore targeted the RRNA, by extracting data from SRs only for domains 1 and 2 and targeted the main RRNA to include primary studies for domains 3–7, identified by searching PubMed, Embase and Scopus from 1 January 2022 to 5 April 2024. Screening of search results by title and abstract, followed by full text screening and data extraction, was undertaken in DistillerSR by two reviewers. Data was extracted using pre-programmed forms to facilitate standardised extractions by one reviewer with a second reviewer checking the extracted data. Data on specific population groups included in the study was extracted as presented by the study authors. Data on the country the study was set in was extracted from the primary studies included in domains 3–7. Disagreements were resolved through consensus or by a third reviewer.

### Mapping to mpox-specific research priorities

We reviewed and extracted both the explicit and implicit research priorities contained in the ‘*United in the fight against mpox in Africa—high level emergency regional meeting communique*’ [15]. We mapped the studies identified from the clinical trial registries, grants captured in the Pandemic PACT Tracker, and evidence identified from the RRNA to these priorities and the immediate research actions outlined in the WHO mpox Coordinated Research Roadmap [13].

### Data analysis

We undertook descriptive analyses of the extracted data to summarise the characteristics of mpox studies registered in the clinical trial registries, mpox grants and published studies. We presented our findings using figures and tables developed in Microsoft Excel, R Studio, Stata, Quantum Geographic Information System (QGIS) and Linearity [23–27].

## Results

### Research funding mapping

As of 1 October 2024, a total of 124 mpox research grants were identified (Additional file 2: Table S2). Funding information was available for 90.3% (*n* = 112/124) of these grants. Of the known financial commitments, the grant amounts awarded ranged from $2,222 to $90 million, with a median value of $418,392 and a combined total investment of at least $215 million invested by 17 funding entities. The National Institutes of Health (NIH) funded most grants, accounting for 45.2% (*n* = 56/124) of the total number, with a total funding amount of $75.5 million (Additional file 3: Fig. S1 details the distribution of grants and financial contributions by funding organisation).

**Fig. 1:**
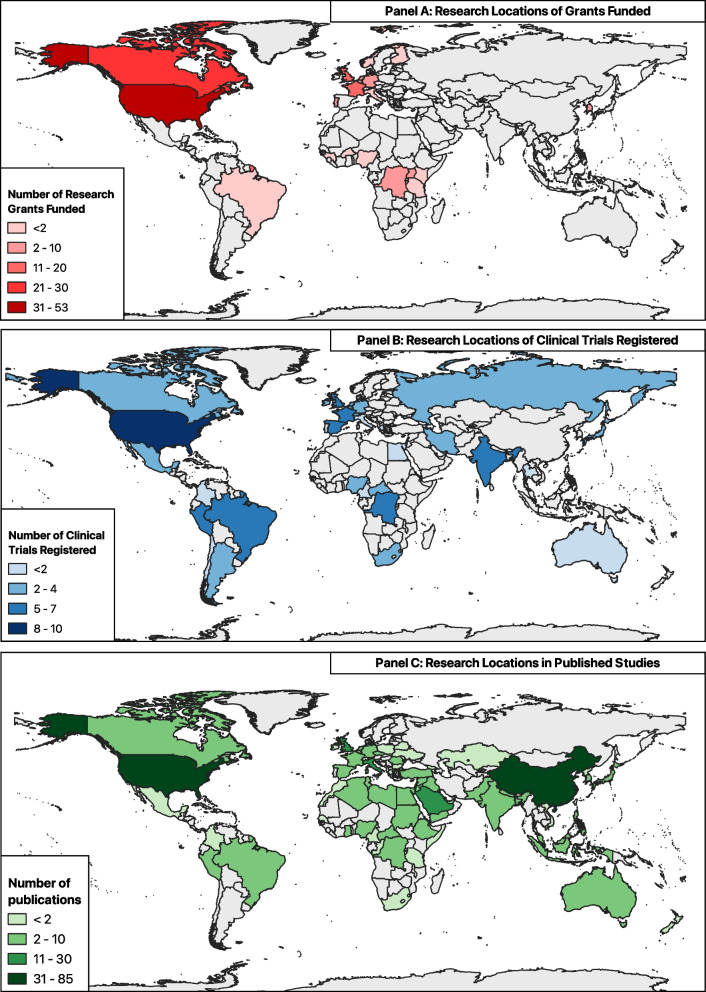
Distribution of mpox research locations. The maps show the past and active mpox research locations identified. **A** Pandemic PACT grant tracker; **B** clinical trial registers; **C** published studies—study setting

Funding was distributed across 18 research location countries, with information on the research locations available for 95.9% (*n* = 119/124) of the grants. Of these, 44.5% (*n* = 53/119) were allocated to research conducted in the USA, amounting to $74 million. In contrast, only 10.5% of the grants (*n* = 13/124) were awarded to research conducted in African countries, with a total investment of $12 million, representing just 5.6% of the overall known funding for mpox research. Among these grants, five were directed to research conducted exclusively in DRC. Other grants were for research across multiple countries (Fig. 1A).

**Fig. 2:**
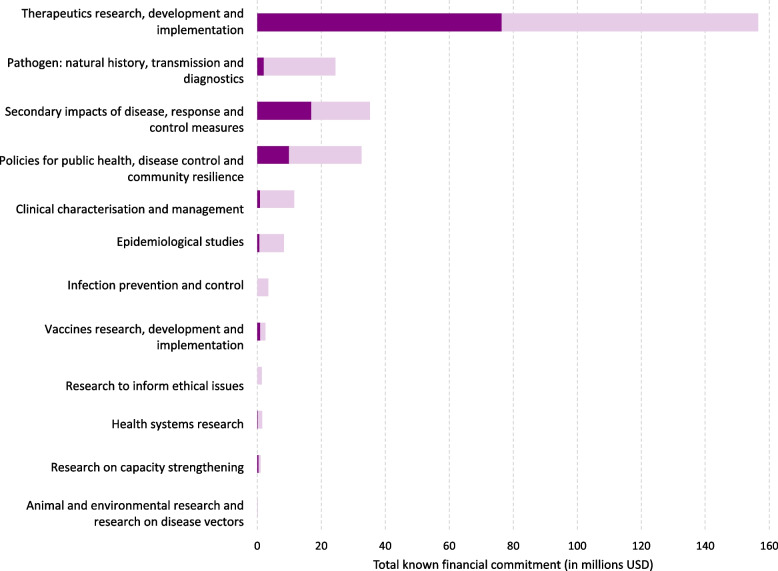
Funding distribution across Pandemic PACT research areas. Dark purple bars represent the minimum known funding amount allocated to grants entirely focussed on a specific research area. The maximum values indicate the potential maximum total funding amount that a particular research area could have received. The light purple bars represent the difference between the minimum known amount and the maximum total funding that could have been allocated to a specific research area if all awards for projects spanning multiple areas had been directed to that specific research area. Approximately 39% (*n* = 48/124) of grants covered more than one research area

When examining funding across research areas, grants focussed on vaccine and therapeutic research were particularly prominent. Vaccine research received at least $118 million, with a potential total financial commitment nearing $134 million. Therapeutics research received at least $32 million, with a potential commitment nearing $55 million. Altogether, these two research areas accounted for at least 69.8% of the total known financial commitment for mpox-related grants. Figure 2 illustrates the distribution of total known financial commitments across research areas, highlighting the minimum known amount, which represents the funding allocated to grants entirely focussed on a specific research area, and the maximum known potential funding these research areas could have received.

Most therapeutics research grants were for pre-clinical studies (48.1%, *n* = 13/27). Clinical trials with unspecified phases comprised 25.9% (*n* = 7/27) of grants, while 3.7% (*n* = 1/27) were phase III trials. No phase I, II or IV trials were identified. One grant (3.7%) focussed on testing repurposed drug treatments, and another (3.7%) on adverse events associated with therapeutic administration. For four grants (14.8%, *n* = 4/27), the specific sub-area of focus could not be determined. The most frequently studied sub-area of vaccine research was the characterisation of vaccine-induced immunity, which was the focus of five grants (16.7%, *n* = 5/30). Two additional grants also addressed this topic, one alongside pre-clinical research and another in combination with vaccine design and administration. Pre-clinical studies alone accounted for four grants (13.3%, *n* = 4/30), while clinical trials with unspecified phases made up another four (13.3%, *n* = 4/30). Three grants (10.0%, *n* = 3/30) were phase II clinical trials, with one additional grant spanning both phase I and II, and another identified as a phase III trial. Five grants focussed on vaccine logistics, including design and administration, design and infrastructure, or both. For five grants (16.7%, *n* = 5/30), the specific sub-area of focus could not be determined.

Of the 124 mpox grants, 40.3% (*n* = 50/124) focussed on investigating pathogen biology, making it the area with the highest number of grants. However, despite this volume, the financial commitments were lower (at least $7 million, with a potential total commitment nearing $37 million) compared to vaccine and therapeutic research. Of 50 grants, 20% (*n* = 10/50) were allocated for the development of mpox diagnostics or diagnostic products.

Only two grants specifically mentioned a focus on a particular mpox clade. One was a grant for a cohort study focussed on the clinical, virologic and immunologic characterisation of mpox virus clade IIb. The other aimed to both strengthen the response and characterise clade Ib, covering its clinical presentation, mode of transmission, at-risk populations and virological evolution.

The study subjects also varied: 29.8% (*n* = 37/124) focussed on human populations; 20.2% (*n* = 25/124) on viruses; 8.1% (*n* = 10/124) on animals; 0.8% (*n* = 1/124) on the environment; and 12.1% (*n* = 15/124) on other subjects, such as chemical compounds and wastewater. Grants involving more than one type of study subject accounted for 17.7% (*n* = 22/124) and 9.7% (*n* = 12/124) did not specify the study subject. Seventeen grants specifically targeted vulnerable groups, with most (71%, *n* = 12/17) focussing on sexual and gender minorities, including MSM or sex workers, while only three (17%, *n* = 3/17) specifically targeted pregnant women.

### Registered studies in clinical trial registries

**Fig. 3:**
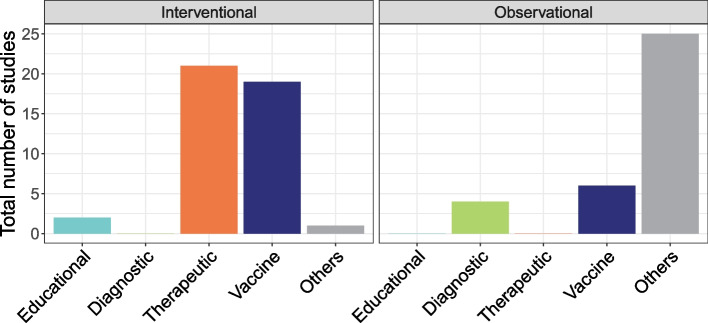
Primary objective of mpox studies registered in clinical trial registries. Note: There was one expanded access study on tecovirimat

We identified 79 studies for which ‘monkeypox’ or ‘mpox’ was the primary condition studied. The study types were interventional (54.4%, *n* = 43/79), observational (44.3%, *n* = 35/79) and expanded access (1.3%, *n* = 1/79) studies (Fig. 3). Of these, 39.2% (*n* = 31/79) indicated that they are actively recruiting patients. None of the studies mentioned a focus on a specific mpox clade.

The studies were set in 33 countries (Fig. 1B), with most located in the USA (12.6%, *n* = 10/79), Brazil (8.9%, *n* = 7/79), India (7.6%, *n* = 6/79) and the DRC (7.6%, *n* = 6/79). In 13.9% (*n* = 11/79) of studies, multiple countries were involved. Other research locations in Africa were Nigeria (*n* = 4/79), South Africa (*n* = 3/79), Central African Republic (*n* = 2/79) and Egypt (*n* = 1/79). Analysis of the geographic locations of the primary sponsors of the studies showed most of studies were sponsored by organisations based in Europe or the USA. Only one study was sponsored by an organisation in Africa (Beni-Suef University, Egypt).

There were 38 clinical trials identified (Additional file 4: Table S3). Of these, 21 were therapeutic trials, with most (71.4%, *n* = 15/21) investigating tecovirimat, followed by Vaccinia immunoglobulin (9.5%, *n* = 2/21). Other therapeutics researched in one study each were cidofovir-probenecid, trifluridine, capsule of edible fungi (*Ganoderma*, *Lentinula edodes*, *Trametes versicolor*, *Grifola frondosa*) and NIOCH-14. There was one expanded treatment access study on tecovirimat.

There were 17 vaccines trials, with the majority (52.9.0%, *n* = 9/17) investigating Modified Vaccinia Ankara—Bavarian Nordic (MVA-BN JYNNEOS or IMVANEX). Other vaccine candidates were LC16 vaccines—LC16, LC16m8 and LC16 KMB (17.6%, *n* = 3/17); VAC∆6 vaccine (11.8%, *n* = 2/17); RNA-based vaccines (11.8%, *n* = 2/17); and dry cell-cultured smallpox vaccine (5.3%; *n* = 1/19). Educational interventions (2.5%; *n* = 2/79) evaluated the impact of educating specific groups in disease prevention and preparedness on mpox control.

### RRNA—mapping of existing published evidence

We identified 415 studies (see Table 1, Additional file 5: Table S4 and Additional file 6: Table S5) comprising SRs (14.5%, *n* = 60/415) and primary studies (85.5%, *n* = 355/415). Only 10.8% (*n* = 45/415) of the studies reported on mpox clade. Of these, 2.2% (*n* = 1/45) reported on clade I, 40.0% (*n* = 18/45) on clade II, and 57.8% (*n* = 26/45) on clade I and II. Most of the SRs (53.3%, *n* = 32/60) focussed on clinical characterisation and transmission (23.3%, *n* = 14/60). One SR (2022), focussed on complications and transmission in pregnant women, reported high rates of maternal death among mpox cases (33.8%, *n* = 375/1109, 95% CI, 28.0–41.0%), intrauterine death (10.4%, *n* = 27/260) and vertical transmission 8.2% (*n* = 4/49) [28].

**Table 1:**
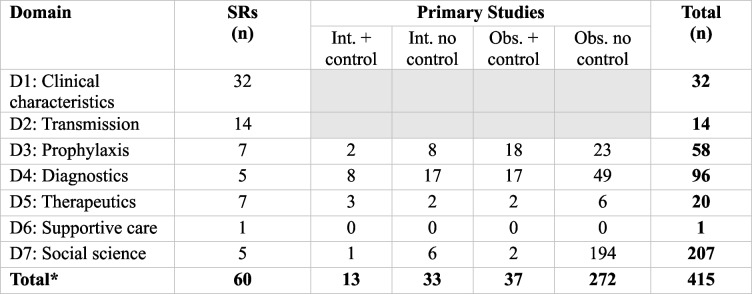
Summary of published studies by study design and domain

*Abbreviations*: *D* domain, *SRs* systematic reviews, *Int.* interventional studies, *Obs*. observational studies; grey shading: data was only extracted from SRs for domains 1 and 2 due to data saturation. *Some studies reported on more than one domain

Most (57.7%, *n* = 205/355) primary studies identified for domains 3 to 7 were set in high-income countries (HICs), 34.4% (*n* = 122/355) in low- and middle-income countries (LMICs), and 9% (*n* = 32/355) had unspecified research setting. Despite regular outbreaks in African countries for decades, most studies were set in the USA (23.1%, *n* = 82/355), China (13.2%, *n* = 47/355), Italy (7.3%, *n* = 26/355), Saudi Arabia (6.5%, *n* = 23/355), UK (3.4%, *n* = 12/355) and the Netherlands (2.8%, *n* = 10/355) (Fig. 1C). Only 6.8% (*n* = 24/355) were set in Africa with 0.8% (*n* = 3/355) in DRC. There was a steep increase in publications following the 2022 multi-country outbreak, most set in HICs (Additional file 7: Fig. S2).

Most (87.0%, *n* = 309/355) were observational and 13.0% (*n* = 46/355) interventional studies. We only identified one randomised controlled trial focussed on MVA-BN vaccination [29]. While most (73.5%, *n* = 261/355) studies included adults, fewer included risk groups such as children under five (3.7%, *n* = 13/355) and 5 to 18 years old (6.2%, *n* = 22/355), older adults > 65 years old (7.9%, *n* = 28/355), people living with HIV (PLHIV) (8.5%, *n* = 30/355) and MSM (17.7%, *n* = 63/355). None reported inclusion of pregnant women.

Most studies focussed on social sciences (56.9%, *n* = 202/355), followed by diagnostics (25.6%, *n* = 91/355), pre- and post-prophylaxis (14.4%, *n* = 51/355) and therapeutics (3.7%, *n* = 13/355). The social science studies were mainly uncontrolled surveys exploring knowledge and attitudes to mpox set in HICs during the 2022 outbreak. There were 13 studies focussed on therapeutics: eight observational, five interventional set in HICs (11 focussed on tecovirimat, two on cidofovir). Of the therapeutic studies, 92.3% (*n* = 12/13) reported inclusion of adults (> 17 years), 7.7% (*n* = 1/13) children (< 18 years), 30.8% (*n* = 4/13) PLHIV, 23.1% (*n* = 3/13) MSM and 7.7% (*n* = 1/13) unspecified. There were no studies identified evaluating effectiveness of supportive care. Fifty-one studies focussed on vaccines, most (86.3%, *n* = 44/51) on the MVA vaccine. Of the vaccine studies, 17.6% (*n* = 9/51) reported inclusion of children (< 18 years), PLHIV 13.7% (*n* = 7/51), and MSM 11.8% (*n* = 6/51). Only one vaccine study was set in DRC in 2022.

### Alignment of recent, ongoing research and published evidence to outbreak priorities

**Fig. 4:**
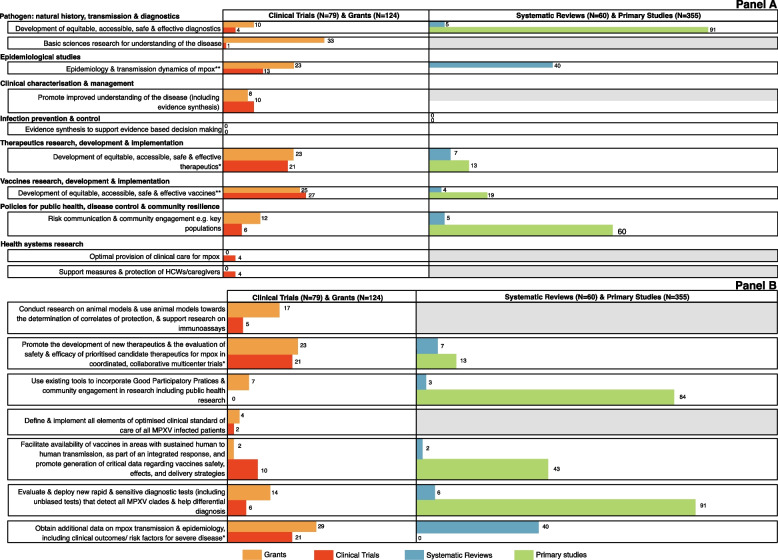
Mpox research grants, registered trials and published studies and mapped to identified from the ‘*United in the fight against mpox in Africa—high level emergency regional meeting communique*’ [15] (**A**) and mapped to actions outlined in the WHO mpox Coordinated R&D Roadmap [13] (**B**). Mpox grants with projects registered in the clinical trials registries (*n* = 6) were not de-duplicated: * represents one overlapping study and ** represents two overlapping studies. Greyed out boxes indicate areas that are out of scope of the RRNA domains. Studies can map to one or more research categories

Figure 4A shows research grants, clinical trial registry data and published studies mapped to mpox priorities identified from the *mpox high-level emergency regional meeting communique*. Research activities were identified from at least one data source for all the priorities except evidence synthesis for supporting infection prevention and control (IPC) measures. Most mpox published systematic reviews focussed on epidemiology and disease transmission (*n* = 40/60). Primary mpox studies were on the development of mpox diagnostics (*n* = 91/355) and risk communication and community engagement (*n* = 60/355).

Most funded mpox research grants focussed on research to improve the understanding of mpox (*n* = 33/124), followed by vaccines R&D (*n* = 25/124), therapeutics R&D (*n* = 123/124), epidemiological research (*n* = 123/124) and therapeutics R&D (*n* = 23/124). The majority of data from the clinical trial registries mapped to priorities under vaccines (*n* = 27/79) and therapeutics (*n* = 21/79) research. Other areas covered were epidemiological studies (*n* = 13/79) and enhancing understanding of mpox (*n* = 10/79).

Few studies in the clinical trial registries focussed on health systems research including optimal care provision for mpox (*n* = 4/79) or support measures for healthcare workers (*n* = 4/79) while none of the mpox grants focussed on these areas. Across the data sources, there was limited focus on engagement approaches in people living with HIV, immunocompromising conditions and other key populations. No grants focussed on identifying mpox IPC measures informed by evidence synthesis.

When mapped to the immediate priorities outlined in the mpox WHO Coordinated Research Roadmap, data included from all three data sources aligned to seven of the ten outbreak response goals (Fig. 4B). The other three actions were implementation actions rather than specific research priorities and were not aligned to our data.

The majority of published primary studies focussed on mpox diagnostics (*n* = 91/355) and community engagement in public health measures (*n* = 84/355). Most systematic reviews were on mpox transmission and epidemiology (*n* = 40/60). Most grants focussed on investigating mpox epidemiology (*n* = 29/124) and development of therapeutics (*n* = 23/124). Most (*n* = 42/79) studies identified from the clinical trial registries similarly focussed on these research areas. Other areas covered by mpox grants were basic science research on animal models for disease characterisation (*n* = 17/124) and development of mpox diagnostics. Mpox grants (*n* = 16) and clinical trials data (*n* = 2), with insufficient project descriptions, could not be mapped to any of the outbreak-specific priorities outlined in either the WHO mpox Roadmap or the *mpox high-level emergency regional meeting communique*.

## Discussion

This study provides a comprehensive analysis of the global health research landscape for mpox, mapping ongoing research activities and scientific evidence from published studies. While mpox has primarily affected West and Central Africa since 1970, most research has been led by institutions in HICs, particularly in the USA and Europe. In contrast, research initiatives in African countries, where mpox outbreaks have had the greatest human impact, remain limited. Although the global response to the 2022 outbreak led to increased research activities, which may support the development of medical countermeasures, most investments were concentrated in Asia, Europe and North America rather than endemic regions.

The resurgence of mpox in the DRC in 2022 underscores these disparities. Despite the increase in research activities, only a third of grants and a quarter of registered trials in 2024 included the DRC as a research location. More broadly, our findings show that only 35% of grants and 39% of registered studies involved African countries, with even fewer focussing specifically on LMICs or the DRC. This lack of locally relevant research poses significant challenges to effective outbreak response. Aligning research with and reorienting future investments to the priorities identified by affected communities is critical to addressing urgent needs, improving national response capacity and preventing further spread.

Our analysis also shows that mpox research has primarily focussed on vaccines, therapeutics and diagnostics, all aligning with global and regional priorities. However, critical gaps remain. For instance, despite progress in developing diagnostic tools, the absence of licensed antigen-based rapid diagnostic tests (RDTs) [30] has hampered case detection and surveillance efforts, especially in resource-limited settings. Additionally, most clinical trials have centred on tecovirimat. Although preliminary findings from the PALM trial indicate tecovirimat did not significantly reduce the duration of mpox lesions in hospitalised patients, there could be potential benefits for early disease and severe mpox cases [31]. Results from PALM 007 extension (which includes paediatric participants) and other ongoing trials such as the UNITY trial will provide important evidence for mpox treatment. Further research will also be needed to evaluate optimal standard of care in vulnerable groups like pregnant women and individuals in immunocompromised states.

Vaccine research has predominantly explored the MVA-BN and LC16 KMB vaccines, both supported by the WHO, while ACAM 2000, which was licensed prior to the ongoing outbreak, are in use for the prevention of mpox in many HICs [32]. Yet access to vaccines in outbreak epicentres remains a challenge, with supply failing to meet the projected demand in Africa [33, 34]. Research into vaccine acceptance, rollout strategies and effectiveness in post-exposure prophylaxis is urgently required to improve prevention measures across affected regions.

Few research grants or publications focussed on specific mpox clades. Studies have largely characterised clade IIb in adult populations while limited investment has been made in understanding clade I, which is driving the current outbreak in the DRC and neighbouring countries. Basic science research on clade I, along with clinical trials targeting at-risk groups, including children and people living with HIV, is essential to inform public health interventions.

A coordinated effort is required to address these and other identified gaps in mpox research including characterising mpox transmission and disease spectrum; evidence synthesis for informing IPC measures, research on optimal forms of support for frontline workers and research focussing on disease stigma, risk behaviour and attitudes. Furthermore, social sciences research specific to the DRC, as well as other endemic LMICs, is necessary to understand effective community and political engagement strategies that can support control of the outbreak. Framing these priorities within a long-term research agenda, not only for emergency response but for sustained capacity-building, will be critical for improving outcomes in the current outbreak and enhancing global preparedness for future threats.

This study acknowledges several limitations. First, a key limitation of the Pandemic PACT dataset is that, while it employs a robust methodology to capture all publicly available research funding data, it may not encompass all global research activity. To mitigate this, we made collaborative efforts to engage a wide range of funders beyond those already contributing to the platform. However, these efforts were constrained by challenges such as limited contact networks, the lack of publicly accessible grant databases and the limited capacity of some funders, particularly those publishing award information in languages other than English, to share data in a standardised format. Despite these constraints, the Pandemic PACT database is designed to be dynamic and continuously updated. As new partnerships are established and additional data sources are integrated, its coverage of the global research funding landscape continues to expand. Yet, while it provides the most comprehensive database of funded mpox research available, it inherently cannot offer an exhaustive view of the global research funding landscape. Capturing a complete picture of this global effort is challenging, particularly due to the lack of publicly available data on commercial research activities, which are often restricted by intellectual property considerations. Nonetheless, the extensive data on public and philanthropic funding for research offers valuable insights that can enhance coordination efforts. Furthermore, due to the large number of identified publications, we had to target data extraction by study design and mapping of the data to identify key gaps in the evidence base. Nonetheless, our data highlights important gaps in the evidence base for mpox, urgently needed to be addressed to protect vulnerable populations from mpox infection, reduce risk of transmission and mitigate impact.

## Conclusions

The recognition of mpox by the Africa CDC and the WHO as a Public Health Emergency of International Concern highlights the urgent need for timely and targeted research to improve outcomes. In this study, we analysed the alignment of ongoing research grants, mpox publications and registered clinical trials to regional and global research priorities. We identified key gaps related to mpox disease characterisation particularly in vulnerable populations, limited containment measures for mpox clade 1 and health systems research in affected countries. Addressing these gaps is critical to improving global preparedness and response. Although a coordinated and inclusive research agenda is already in place, our findings highlight gaps in funding alignment with its priorities. Ensuring that research efforts and investments are better coordinated with this agenda is essential to accelerate the generation of actionable evidence, improve the efficient use of resources and support meaningful outcomes in disease control, population health and the resilience of health systems.

## Supplementary Information

Additional file 1: Table S1 Pandemic PACT research categories and sub-categories expanded from the figure in the supplementary appendix of ‘Improving coherence of global research funding: Pandemic PACT’

Additional file 2: Table S2 Mpox research grants identified from the Pandemic PACT Grant Tracker

Additional file 3: Fig. S1 Distribution of mpox grants and financial contributions by funding organisations

Additional file 4: Table S3 Distribution of interventions investigated in mpox clinical trials in PACTR, SANCTR and WHO ICTRP

Additional file 5: Table S4 Systematic reviews included in the rapid research needs appraisals

Additional file 6: Table S5 Primary studies included in the rapid research needs appraisals

Additional file 7: Fig. S2 Evolution of mpox publications over time

## Data Availability

The datasets on grants and clinical trials generated and analysed during the current study are available in the Figshare (doi: https://figshare.com/s/60caa900191611cff07d?file=54703955 and https://figshare.com/s/58527668245cb63f14f5/articles/26937448, respectively). The datasets on published mpox studies analysed during the current study are available in Additional files 5 and 6 of this paper.

## Abbreviations

CORC: Collaborative Open Research Consortium
DRC: Democratic Republic of the Congo
HIC: High-income countries
ICTRP: WHO International Clinical Trials Registry Platform
IPC: Infection prevention and control
LMIC: Low- and middle-income countries
MSM: Men who have sex with men
PACTR: Pan African Clinical Trial Registry
Pandemic PACT: Pandemic Preparedness Analytical Capacity and Funding Tracking Programme
RDT: Rapid diagnostic tests
RRNA: Rapid research needs appraisal
SANCTR: South African National Clinical Trial Register
WHO: World Health Organization
