# Supplementary material for: Gaps in the global health research landscape for mpox: an analysis of research activities and existing evidence
Source: BMC Med. 2025 Sep 29;23:522. doi: 10.1186/s12916-025-04350-1 (PMC12482760; doi:10.1186/s12916-025-04350-1)
Supplement: Supplementary file 1 — Additional file 1: Table S1 Pandemic PACT research categories and sub-categories expanded from the figure in the supplementary appendix of ‘Improving coherence of global research funding: Pandemic PACT’ [file 12916_2025_4350_MOESM1_ESM.docx]

# **Additional file 1: Table S1**. Pandemic PACT research categories and subcategories expanded from the figure in the supplementary appendix of “Improving coherence of global research funding: Pandemic PACT”^1^

| Broad categories | | Sub-categories | Description |
| --- | --- | --- | --- |
| 1. Pathogen: natural history, transmission and diagnostics | | Diagnostics | Research leading to development of diagnostics and diagnostic products for different pathogen strains and contexts (e.g., point of care tests and lab-based diagnostics: RDTs, PCR, ELISA, etc.). Includes basic research and more advanced stages of diagnostics/ diagnostic product development. |
|  |  | Pathogen morphology, shedding & natural history | Research on pathogen compartments and infectivity, pathogen's features associated with disease transmission, Pathogen structure; Replication and transmissibility; Shedding and pathogenesis. |
|  |  | Pathogen genomics, mutations and adaptations | Research on Pathogen genotyping; New or existing variants or strains; Vaccine resistance; Antimicrobial resistance (AMR). |
|  |  | Immunity | Research on various aspects of immunity including naturally acquired immunity (research investigating immunity over time, re-infection, duration/ strength of immunity, Population immunity/ herd immunity); Vaccine-induced immunity. |
|  |  | Disease models | Research on research models including the development of models e.g., animal models, 3Rs approaches including organoids, explant models etc. and Human challenge models |
|  |  | Environmental stability of pathogen | Research on the viability/infectiousness of pathogen under variable environmental conditions. |
|  |  | N/A | Applied in instances where research grants/ projects fall under the broad category but are outside the established sub-categories under "Pathogen: natural history, transmission and diagnostics". |
|  |  | Unspecified | Applied in instances where there is insufficient information to sub-categorise under "Pathogen: natural history, transmission and diagnostics" but fall under this broad category. |
| 2. Animal and environmental research and research on diseases vectors | | Animal source and routes of transmission | Research on animal source of pathogens, reservoirs, Spillover events; human-animal transmission; animal-animal transmission; vector-human transmission; vector-animal -human transmission. |
|  |  | Vector biology | Research on disease natural history in vector including pathogenesis in disease vector; Impact of environmental change on vector e.g. bite rates, timing of bites etc. |
|  |  | Vector control strategies | Research on: Biological control (e.g., natural enemy conservation, biological larvicides, fungi, botanicals etc.) Chemical control (e.g., Insecticide treated bed nets, indoor residual spraying, chemical repellents etc.) Environmental control (e.g., habitat manipulation, irrigation management and design, waste management etc.) Mechanical control (e.g., House improvements etc.) community participation (community mobilisation and education, community adherence and acceptance of control methods) |
|  |  | N/A | Applied in instances where research grants/ projects fall under the broad category but are outside the established sub-categories under "Animal and environmental research and research on disease vectors". |
|  |  | Unspecified | Applied in instances where there is insufficient information to sub-categorise under "Animal and environmental research and research on disease vectors" but fall under this broad category. |
| 3. Epidemiological studies | | Disease transmission dynamics | Epidemiological studies on human-to-human transmission and environmental transmission; assessments of routes length of infectiousness, incubation period of disease etc. |
|  |  | Disease susceptibility | Epidemiological studies to characterise severe disease and susceptible populations, identifying risk factors for infection. |
|  |  | Impact/ effectiveness of control measures | Epidemiological studies to assess effectiveness of various control measures on disease transmission. |
|  |  | Disease surveillance & mapping | Disease burden studies - incidence and prevalence studies, QALYs, DALYs, Studies on morbidity & mortality. Disease surveillance studies e.g., serosurveillance studies, wastewater surveillance, genome surveillance; disease reporting |
|  |  | N/A | Applied in instances where research grants/ projects fall under the broad category but are outside the established sub-categories under "Epidemiological studies". |
|  |  | Unspecified | Applied in instances where there is insufficient information to sub-categorise under "Epidemiological studies" but fall under this broad category. |
| 4. Clinical characterisation and management | | Prognostic factors for disease severity | Research assessing factors predisposing to severe disease e.g. genetic factors, physical factors co-morbidities, co-infection, biomarkers etc. |
|  |  | Disease pathogenesis | Spectrum of disease - mild, moderate and severe disease |
|  |  | Supportive care, processes of care and management | Research assessing interventions for improving clinical outcomes of disease, disease diagnosis, management and discharge processes |
|  |  | Post acute and long-term health consequences | Research assessing physical health sequelae of infections, burden of disease (incidence, prevalence of post-acute conditions) |
|  |  | Clinical trials for disease management | Clinical trials for assessing interventions for disease management e.g. supportive care, lengths of admission. NB: This does not apply to solely therapeutic trials |
|  |  | N/A | Applied in instances where research grants/ projects fall under the broad category but are outside the established sub-categories under "Clinical characterisation and management". |
|  |  | Unspecified | Applied in instances where there is insufficient information to sub-categorise under "Clinical characterisation and management" but fall under this broad category. |
| 5. Infection prevention and control | | Restriction measures to prevent secondary transmission in communities | Research assessing approaches/ effectiveness of quarantine & other movement restriction measures targeting of unexposed, exposed or uninfected individuals, restriction on animal movement and contact; Effectiveness/development of measures for community settings (population level control measures) including congregate settings e.g., schools, workplaces, farms etc. |
|  |  | Barriers, PPE, environmental, animal and vector control measures | Research assessing barriers between wearer and environment e.g., Masks, goggles Hazmat suits or disinfection of environment e.g., chemical deactivation of pathogens, vector control measures such as bed nets and repellents. |
|  |  | IPC in health care settings | Research assessing effectiveness/development of measures for health care setting including community healthcare facilities, ICUs, care homes, etc and involving various cadres of health care staff |
|  |  | IPC at the human-animal interface | Research assessing effectiveness/development of measures at the human animal interface e.g., environmental modification |
|  |  | N/A | Applied in instances where research grants/ projects fall under the broad category but are outside the established sub-categories under "Infection prevention and control" but fall under this broad category. |
|  |  | Unspecified | Applied in instances where there is insufficient information to sub-categorise under "Infection prevention and control" but fall under this broad category. |
| 6. Therapeutics research, development and implementation | | Pre-clinical studies | Research on early stages of drug development including laboratory- based studies, testing in animal/ cell models |
|  |  | Phase 0 clinical trial | First in human testing for safety and immunogenicity in a few human participants. |
|  |  | Phase 1 clinical trial | Research assessing safety and immunogenicity testing in small group of human participants. |
|  |  | Phase 2 clinical trial | Research assessing safety and immunogenicity testing in a larger group of human participants. |
|  |  | Phase 3 clinical trial | Larger scale testing of therapeutics for efficacy and safety. |
|  |  | Phase 4 clinical trial | Post-marketing surveillance (after licensing) for long term efficacy and detect rare adverse effects |
|  |  | Prophylactic use of treatments & Repurposed drugs | Discovery, testing and evaluation of prophylactic therapeutics or research on licensed drugs which are repurposed for treatment/prevention of infections. |
|  |  | Clinical trial (unspecified trial phase) | Clinical trials with no specific trial phase stated, or randomized control trials. |
|  |  | Therapeutics logistics and supply chains and distribution strategies | Research on therapeutics: stability and storage; Access; Cost; Supply; Regulation of therapeutics; Stabilised manufacturing, i.e., reliable, robust (GMP); Transport of therapeutics |
|  |  | Therapeutic trial design | Research on clinical trial capacity and novel designs for therapeutic clinical trials |
|  |  | Adverse events associated with therapeutic administration | Research assessing adverse events associated with therapeutic use - studies outside of clinical trials e.g., surveillance surveys, prevalence studies etc.) |
|  |  | N/A | Applied in instances where research grants/ projects fall under the broad category but are outside the established sub-categories under "Therapeutics research, development, and implementation". |
|  |  | Unspecified | Applied in instances where there is insufficient information to sub-categorise under "Therapeutics research, development, and implementation" but fall under this broad category. |
| 7. Vaccines research, development and implementation | | Pre-clinical studies | Research on early stages of drug development including lab based, testing in animal/ cell models etc. |
|  |  | Phase 0 clinical trial | First in-human testing for safety (a few human participants) |
|  |  | Phase 1 clinical trial | Safety and efficacy testing in small group of human participants |
|  |  | Phase 2 clinical trial | Safety and efficacy testing in a larger group of human participants |
|  |  | Phase 3 clinical trial | Larger scale testing for efficacy, effectiveness, and safety |
|  |  | Phase 4 clinical trial | Post-marketing surveillance studies (after licensing) for long term efficacy and for detection of rare adverse effects |
|  |  | Clinical trial (unspecified trial phase) | Clinical trials with no specific trial phase stated, or randomized control trials |
|  |  | Vaccine logistics and supply chains and distribution strategies | Research on Vaccines: stability and storage; Access; Cost; Supply; Regulation of vaccines; Stabilised manufacturing, i.e., reliable, robust (GMP); Transport of vaccines |
|  |  | Vaccine design and administration | Research assessing vaccine formulation and mode(s) of delivery |
|  |  | Vaccine trial design and infrastructure | Clinical trial capacity and novel designs for vaccine clinical trials |
|  |  | Adverse events associated with immunization | Research assessing adverse events associated with immunization - e.g., surveys, prevalence studies etc. |
|  |  | Characterisation of vaccine-induced immunity | Research characterising vaccine-mediated immune responses (studies outside of clinical trials e.g., surveillance surveys, prevalence studies etc.) |
|  |  | N/A | Applied in instances where research grants/ projects fall under the broad category but are outside the established sub-categories under "Vaccine research, development and implementation". |
|  |  | Unspecified | Applied in instances where there is insufficient information to sub-categorise under "Vaccine research, development and implementation" but fall under this broad category. |
| 8. Research to inform ethical issues | | Research to inform ethical issues in Research | Research on ethical issues inclusive of research design, conduct of research, funding of research, research priority setting, data and benefit sharing, publication/dissemination of research, use of unproven clinical interventions outside clinical trials during public health emergencies (MEURI), other research ethics issues, etc. |
|  |  | Research to inform ethical issues related to Public Health Measures | Research on ethical issues inclusive of the use of non-pharmaceutical interventions/'restrictive' public health measures, vaccination, surveillance, contact tracing, vaccine mandates/passports, mask policies, etc. |
|  |  | Research to inform ethical issues in Clinical and Health System Decision-Making | Research on clinical / health care decision making including prioritisation of resources in health systems, care protocol/ treatment guidelines for affected individuals etc. |
|  |  | Research to inform ethical issues in the Allocation of Resources | Research on processes for allocation of vaccines, therapeutics, diagnostics, PPE, ventilators, etc. |
|  |  | Research to inform ethical issues in Governance | Research assessing governance processes for infection/outbreak prevention, preparedness, response, recovery, and research efforts. |
|  |  | Research to inform ethical issues related to Social Determinants of Health, Trust, and Inequities | Research assessing social factors involved in avoidable differences in burden of disease or in achieving quality health, access to health care products, diagnostics, healthcare monitoring, and delivery tools, social factors predisposing to severe disease etc. |
|  |  | N/A | Applied in instances where research grants/ projects fall under the broad category but are outside the established sub-categories under "Research to inform ethical issues". |
|  |  | Unspecified | Applied in instances where there is insufficient information to sub-categorise under "Research to inform ethical issues" but fall under this broad category. |
| 9. Policies for public health, disease control, and community resilience | Approaches to public health interventions | | Research on ways to promote acceptance, uptake, and adherence to public health measures, healthcare monitoring, and delivery tools. Research on the perception/ understanding of public health interventions. |
|  | | Community engagement | Research on effective approaches to engagement - patient and community engagement and collective responses. Four Approaches to community engagement (WHO 2020): Community-oriented, Community-based, Community-managed, community-owned |
|  |  | Communication | Research on effective approaches to risk communication, communication of disease, public health interventions etc. Impacts of misinformation on PH measures, public health messaging, infodemic management etc |
|  |  | Vaccine/Therapeutic/ treatment hesitancy | Research assessing trust/ acceptance/uptake of vaccines and therapeutics and other treatments, interventions and diagnostics |
|  |  | Policy research and interventions | Policy research including on public health policy and non-public health policy; economic evaluations to inform policy. |
|  |  | N/A | Applied in instances where research grants/ projects fall under the broad category but are outside the established sub-categories under "Policies for public health, disease control and community resilience". |
|  |  | Unspecified | Applied in instances where there is insufficient information to sub-categorise under "Policies for public health, disease control, and community resilience" but fall under this broad category. |
| 10.Secondary impacts of disease, response & control measures | | Indirect health impacts | Exploring or mitigating Health impacts beyond the infection on mental health, co-morbidities e.g., NCDx, Infectious diseases, etc.  health care services e.g., immunisations, nutrition programmes, impacts on health systems etc. |
|  |  | Social impacts | Research exploring or mitigating impacts of disease on: Education; Social protection services; Justice and judicial system etc. & impacts on social services |
|  |  | Economic impacts | Research exploring or mitigating Impacts of disease on: Economy and work; Macroeconomic policies; Socioeconomic policies etc. |
|  |  | Other secondary impacts | Research exploring or mitigating impacts of disease on: Environment; Food Security; Supply Chains; Infrastructure; or Policy and governance. |
|  |  | N/A | Applied in instances where research grants/ projects fall under the broad category but are outside the established sub-categories under "Secondary impacts of disease, response & control measures". |
|  |  | Unspecified | Applied in instances where there is insufficient information to sub-categorise under "Secondary impacts of disease, response & control measures" but fall under this broad category. |
| 11.Health Systems Research | | Health service delivery | Research on health service delivery including health care coverage, continuity, quality and accessibility of range of care services including in-patient, out-patient care, community-based services, emergency and routine care etc. |
|  |  | Health financing | Research on health financing, including direct and indirect health care costs, Health care payment models - out of pocket payments, health insurance coverage etc. Economic evaluations e.g., cost effectiveness/ cost utility assessments of various interventions etc |
|  |  | Medicines, vaccines & other technologies | Research on: Access (focus on vulnerable, marginalised groups); distribution; storage of products; Supply chains; regulations; standards; guidelines and policies for utilisation/ implementation. |
|  |  | Health information systems | Research on health information systems including utilisation of health information systems and databases; Acquisition of health information management, storage and evaluation of health information including acceptability, confidentiality, privacy etc. |
|  |  | Health leadership and governance | Research on health systems governance including health policy research; Policy implementation; Health management |
|  |  | Health workforce | Research on health workforce including training, management and retention of various cadres of health care staff including staff satisfaction, motivation etc. |
|  |  | N/A | Applied in instances where research grants/ projects fall under the broad category but are outside the established sub-categories under "Health Systems Research". |
|  |  | Unspecified | Applied in instances where there is insufficient information to sub-categorise under "Health Systems Research" but fall under this broad category. |
| 12. Research on Capacity Strengthening | | Individual level capacity strengthening | Research on individual - capabilities and requirements which enable an individual or members of a research team to undertake good quality research. |
|  |  | Institutional level capacity strengthening | Research on/ assessing organizational/institutional - the improved capacity of scientific research organizations and institutions via enhancements to: infrastructure, adequate staff (including financial and management staff), curricula, acquisition of funds, external contacts, etc. |
|  |  | Systemic/environmental components of capacity strengthening | Research on/ assessing environmental/systemic component including national/ sub-national, research networks and global/ international sub-components. This relates to environmental factors including capacity and commitment at the financing and policy level to promote research capacity, to set standards, or to link policy, research and practice at national and international levels etc. |
|  |  | Cross-cutting | Research on/ assessing activities cutting across all levels of capacity strengthening. |
|  |  | N/A | Applied in instances where research grants/ projects fall under the broad category but are outside the established sub-categories under "Research on Capacity Strengthening". |
|  |  | Unspecified | Applied in instances where there is insufficient information to sub-categorise under "Research on Capacity Strengthening" but fall under this broad category. |
| 13. N/A | | Applied in instances where research grants/ projects fall outside all the 13 broad categories. | |
| 14. Unspecified | | Applied in instances where there is insufficient information for any broad category. | |

**^1^** Norton A, Sigfrid L, Antonio E, Bucher A, Ndwandwe D. Improving coherence of global research funding: Pandemic PACT. The Lancet. Elsevier; 2024 Mar 30;403(10433):1233. PMID: 38518795
