# Supplementary material for: Gaps in the global health research landscape for mpox: an analysis of research activities and existing evidence
Source: BMC Med. 2025 Sep 29;23:522. doi: 10.1186/s12916-025-04350-1 (PMC12482760; doi:10.1186/s12916-025-04350-1)
Supplement: Supplementary file 2 — Additional file 2: Table S2 Mpox research grants identified from the Pandemic PACT Grant Tracker [file 12916_2025_4350_MOESM2_ESM.docx]

# **Additional file 2: Table S2**. Mpox Research grants identified from the Pandemic PACT Grant Tracker

| **PACT ID** | **Start Year** | **End Year** | **Title** | **Known Financial Commitment (in US dollars)** | **Funder** | **Research Institution** | **Study Type** | **Research Area** |
| --- | --- | --- | --- | --- | --- | --- | --- | --- |
| P21093 | 2023 | 2028 | COVID-19 Drug-Screening and Resistance Hub (CRUSH) | 446400 | UK Research and Innovation (UKRI) | University of Glasgow | Non-Clinical | Pathogen: natural history, transmission and diagnostics \| Therapeutics research, development and implementation |
| P21096 | 2022 | 2024 | Pre-symptomatic asymptomatic MonkEypox (PRIME) study | 1260039.55 | UK Research and Innovation (UKRI) | UK Health Security Agency | Clinical | Epidemiological studies \| Clinical characterisation and management \| Vaccines research, development and implementation |
| P21097 | 2023 | 2025 | Restriction of DNA viruses by TRIM5a and ZAP / TRIM25 / KHNYN: mechanisms of restriction and viral evasion | 797341.35 | UK Research and Innovation (UKRI) | University of Oxford | Non-Clinical | Pathogen: natural history, transmission and diagnostics |
| P21098 | 2023 | 2026 | MRC Centre for Global Infectious Disease Analysis (MRC GIDA) | 3659554.08 | UK Research and Innovation (UKRI) | Imperial College London | Non-Clinical | Epidemiological studies \| Infection prevention and control \| Therapeutics research, development and implementation \| Vaccines research, development and implementation |
| P21286 | 2023 | 2027 | Antibody discovery for neglected tropical and emerging infectious diseases | Unspecified | UK Research and Innovation (UKRI) | Liverpool School of Tropical Medicine | Non-Clinical | Therapeutics research, development and implementation |
| P21287 | 2023 | 2027 | Antibody discovery for neglected tropical and emerging infectious diseases | Unspecified | UK Research and Innovation (UKRI) | Liverpool School of Tropical Medicine | Non-Clinical | Therapeutics research, development and implementation |
| P21289 | 2022 | 2023 | Monkeypox Rapid Research Response | Unspecified | UK Research and Innovation (UKRI) | University of Glasgow | Unspecified | Unspecified |
| P21290 | 2022 | 2023 | [Monkeypox] Rapid Research Response | Unspecified | UK Research and Innovation (UKRI) | University of Cambridge | Unspecified | Unspecified |
| P21291 | 2022 | 2023 | Social Science issues relating to Monkeypox | 183238.26 | UK Research and Innovation (UKRI) | University College London | Non-Clinical | Policies for public health, disease control & community resilience |
| P21292 | 2022 | 2023 | [Monkey Pox] Rapid Research Response | Unspecified | UK Research and Innovation (UKRI) | N/A | Clinical | Pathogen: natural history, transmission and diagnostics \| Animal and environmental research and research on diseases vectors \| Therapeutics research, development and implementation \| Vaccines research, development and implementation |
| P21293 | 2020 | 2024 | Viral manipulation of DBC1: a novel strategy to promote cell survival and suppress inflammation | Unspecified | UK Research and Innovation (UKRI) | N/A | Non-Clinical | Pathogen: natural history, transmission and diagnostics |
| P21295 | 2022 | 2023 | High-throughput digital microplate microscopy reader for the study of cellular responses to infection and stress | Unspecified | UK Research and Innovation (UKRI) | N/A | Non-Clinical | Pathogen: natural history, transmission and diagnostics |
| P21520 | 2023 | 2028 | The role of mTOR dysregulation in poxvirus infection | 611807 | National Institutes of Health (NIH) | Northwestern University At Chicago | Non-Clinical | Pathogen: natural history, transmission and diagnostics |
| P21521 | 2023 | 2027 | Understanding the Function of F13 as a Matrix Protein for Poxvirus Intracellular Envelopment | 346500 | National Institutes of Health (NIH) | University Of Rochester | Non-Clinical | Pathogen: natural history, transmission and diagnostics |
| P21522 | 2023 | 2028 | Gigapixel Next-Generation-Sequencing: An Ultra-Sensitive Diagnostic for Infections of the CNS | 549398 | National Institutes of Health (NIH) | University Of California, San Francisco | Non-Clinical | Pathogen: natural history, transmission and diagnostics |
| P21523 | 2023 | 2024 | RADX Independent Test Assessment Program for MPOX Lesion Panel - Coordination Center | 153450 | National Institutes of Health (NIH) | MASSACHUSETTS GENERAL HOSPITAL | Non-Clinical | Pathogen: natural history, transmission and diagnostics \| Health Systems Research |
| P21524 | 2023 | 2024 | RADX Independent Test Assessment Program for MPOX Lesion Panel - Validation Center | 186787 | National Institutes of Health (NIH) | Emory University | Non-Clinical | Pathogen: natural history, transmission and diagnostics \| Health Systems Research |
| P21525 | 2023 | 2024 | RADX Independent Test Assessment Program for MPOX Lesion Panel - Commercialization Center | 659763 | National Institutes of Health (NIH) | VENTUREWELL | Non-Clinical | Pathogen: natural history, transmission and diagnostics \| Health Systems Research |
| P21526 | 2022 | 2024 | Trial to Evaluate the Immunogenicity of Dose Reduction Strategies of the MVA-BN Mpox Vaccine | 6645185 | National Institutes of Health (NIH) | LEIDOS BIOMEDICAL RESEARCH, INC. | Clinical | Vaccines research, development and implementation |
| P21527 | 2023 | 2028 | Research Resources and Workforce Development for the Rocky Mountain Regional Biocontainment Laboratory at Colorado State University | 2751527 | National Institutes of Health (NIH) | COLORADO STATE UNIVERSITY | Not applicable | Not applicable |
| P21528 | 2020 | 2024 | Trial to Evaluate the Immunogenicity of Dose Reduction Strategies of the MVA-BN Mpox Vaccine | 429747 | National Institutes of Health (NIH) | LEIDOS BIOMEDICAL RESEARCH, INC. | Clinical | Vaccines research, development and implementation |
| P21529 | 2021 | 2023 | Statistical Data Coordinating Center: Monkeypox Clinical Research Support | 1300000 | National Institutes of Health (NIH) | THE EMMES COMPANY, LLC | Non-Clinical | Vaccines research, development and implementation |
| P21530 | 2023 | 2027 | Understanding the Function of F13 as a Matrix Protein for Poxvirus Intracellular Envelopment | 385000 | National Institutes of Health (NIH) | University Of Rochester | Non-Clinical | Pathogen: natural history, transmission and diagnostics |
| P21531 | 2020 | 2025 | Advancement of poxvirus inhibitor | 656410 | National Institutes of Health (NIH) | Boston University Medical Campus | Non-Clinical | Therapeutics research, development and implementation |
| P21573 | 2024 | 2028 | The B22 family of orthopoxvirus virulence factors: Investigating structure/function of these potent, multifaceted immunoevasins | 668007 | National Institutes of Health (NIH) | CHILDREN'S HOSP OF PHILADELPHIA | Non-Clinical | Pathogen: natural history, transmission and diagnostics |
| P21574 | 2022 | 2024 | Comprehensive genetic dissection of poxvirus membrane assembly and function | 189000 | National Institutes of Health (NIH) | ALBERT EINSTEIN COLLEGE OF MEDICINE | Non-Clinical | Pathogen: natural history, transmission and diagnostics |
| P21575 | 2023 | 2025 | Poxvirus-encoded noncanonical open reading frames | 183220 | National Institutes of Health (NIH) | TEXAS A&M AGRILIFE RESEARCH | Non-Clinical | Pathogen: natural history, transmission and diagnostics |
| P21576 | 2022 | 2027 | RFA-CK-22-001, Investigation of Monkeypox and Other Zoonotic Diseases in the Democratic Republic of the Congo (DRC) - 2022 | 700000 | National Institutes of Health (NIH) | KINSHASA SCHOOL OF PUBLIC HEALTH | Non-Clinical | Pathogen: natural history, transmission and diagnostics \| Animal and environmental research and research on diseases vectors \| Epidemiological studies \| Therapeutics research, development and implementation \| Vaccines research, development and implementation \| Research on Capacity Strengthening |
| P21579 | 2022 | 2024 | Comprehensive genetic dissection of poxvirus membrane assembly and function | 252000 | National Institutes of Health (NIH) | ALBERT EINSTEIN COLLEGE OF MEDICINE | Non-Clinical | Pathogen: natural history, transmission and diagnostics |
| P21581 | 2022 | 2024 | Monkeypox vaccine clinical trial | 11497606 | National Institutes of Health (NIH) | LEIDOS BIOMEDICAL RESEARCH, INC. | Clinical | Vaccines research, development and implementation |
| P21582 | 2022 | 2024 | A5418 study of tecovirimat for human monkeypox virus (STOMP) | 605168 | National Institutes of Health (NIH) | WESTAT, INC. | Clinical | Therapeutics research, development and implementation |
| P21583 | 2021 | 2022 | Characterize and optimize adjuvants to test monkeypox vaccines | 1599006 | National Institutes of Health (NIH) | University Of Montana | Non-Clinical | Vaccines research, development and implementation |
| P21584 | 2021 | 2022 | Statistical Data Coordinating Center: Monkeypox Clinical Research Support | 435519 | National Institutes of Health (NIH) | THE EMMES COMPANY, LLC | Non-Clinical | Vaccines research, development and implementation |
| P21585 | 2022 | 2023 | TASK V17: development of assays and reagents for monkeypox vaccines | 1045131 | National Institutes of Health (NIH) | BATTELLE CENTERS/PUB HLTH RES & EVALUATN | Non-Clinical | Vaccines research, development and implementation |
| P21586 | 2022 | 2023 | Study of Tecovirimat for Human Monkeypox Virus (STOMP) | 5882075 | National Institutes of Health (NIH) | University Of California Los Angeles | Clinical | Therapeutics research, development and implementation |
| P21587 | 2022 | 2027 | Investigation of Monkeypox and Other Zoonotic Diseases in the Democratic Republic of the Congo (DRC) - 2022 | 875000 | National Institutes of Health (NIH) | KINSHASA SCHOOL OF PUBLIC HEALTH | Clinical \| Non-Clinical | Pathogen: natural history, transmission and diagnostics \| Animal and environmental research and research on diseases vectors \| Epidemiological studies \| Therapeutics research, development and implementation \| Vaccines research, development and implementation \| Research on Capacity Strengthening |
| P21588 | 2021 | 2023 | Targeting of RAG-dependent and -independent innate immune responses by the Ectromelia C15 protein | 220000 | National Institutes of Health (NIH) | CHILDREN'S HOSP OF PHILADELPHIA | Non-Clinical | Pathogen: natural history, transmission and diagnostics |
| P21589 | 2021 | 2024 | Unravelling the mechanisms of virus host species jump | 196250 | National Institutes of Health (NIH) | Arizona State University-Tempe Campus | Non-Clinical | Pathogen: natural history, transmission and diagnostics \| Animal and environmental research and research on diseases vectors |
| P21590 | 2020 | 2025 | Advancement of poxvirus inhibitor | 593447 | National Institutes of Health (NIH) | Boston University Medical Campus | Non-Clinical | Therapeutics research, development and implementation |
| P21591 | 2020 | 2025 | Epidemiologic Studies of HMPXV Infection among PWID in the US-Mexico Border Region to Inform Prevention Responses | 379961 | National Institutes of Health (NIH) | University Of California, San Diego | Non-Clinical | Pathogen: natural history, transmission and diagnostics \| Epidemiological studies |
| P21592 | 2020 | 2025 | A randomized, placebo-controlled trial of the safety and efficacy of tecovirimat for the treatment of patients with monkeypox virus disease | 9824009 | National Institutes of Health (NIH) | LEIDOS BIOMEDICAL RESEARCH, INC. | Clinical | Therapeutics research, development and implementation |
| P21593 | 2021 | 2022 | XXIII International Poxvirus, Asfarvirus, and Iridovirus Conference | 3000 | National Institutes of Health (NIH) | University Of Pennsylvania | Not applicable | Not applicable |
| P21594 | 2021 | 2023 | Targeting of RAG-dependent and -independent innate immune responses by the Ectromelia C15 protein | 264000 | National Institutes of Health (NIH) | CHILDREN'S HOSP OF PHILADELPHIA | Non-Clinical | Pathogen: natural history, transmission and diagnostics |
| P21595 | 2021 | 2023 | Unravelling the mechanisms of virus host species jump | 235500 | National Institutes of Health (NIH) | Arizona State University-Tempe Campus | Non-Clinical | Pathogen: natural history, transmission and diagnostics \| Animal and environmental research and research on diseases vectors |
| P21596 | 2020 | 2025 | Advancement of poxvirus inhibitor | 590147 | National Institutes of Health (NIH) | Boston University Medical Campus | Non-Clinical | Therapeutics research, development and implementation |
| P21597 | 2020 | 2022 | The novel innate immune-antagonistic effects of ectromelia virus C15 protein | 33385 | National Institutes of Health (NIH) | University Of Pennsylvania | Non-Clinical | Pathogen: natural history, transmission and diagnostics |
| P21598 | 2020 | 2025 | Advancement of poxvirus inhibitor | 575718 | National Institutes of Health (NIH) | Boston University Medical Campus | Non-Clinical | Therapeutics research, development and implementation |
| P21599 | 2020 | 2022 | The novel innate immune-antagonistic effects of ectromelia virus C15 protein | 32869 | National Institutes of Health (NIH) | University Of Pennsylvania | Non-Clinical | Pathogen: natural history, transmission and diagnostics |
| P22231 | 2021 | 2024 | Mechanism of membrane inactivation method to prepare enveloped virus vaccines | 195501 | National Institutes of Health (NIH) | CORNELL UNIVERSITY | Non-Clinical | Vaccines research, development and implementation |
| P23419 | 2022 | Unspecified | Characterizing transmission dynamics and evaluating medical countermeasures to inform the clinical and public health response to monkeypox | 730000 | International Development Research Centre (IDRC) | Nigerian Institute of Medical Research | Clinical | Epidemiological studies \| Therapeutics research, development and implementation \| Vaccines research, development and implementation \| Policies for public health, disease control & community resilience |
| P23460 | 2022 | 2026 | Sustainable use and integration of enhanced infrastructure into routine WGS-based surveillance and outbreak investigation activities in Portugal | 1583250.57 | European Commission | INSTITUTO NACIONAL DE SAUDE DR. RICARDO JORGE | Not applicable | Epidemiological studies |
| P23546 | 2023 | 2026 | Vax-Action: tackling effectively vaccine hesitancy in Europe | 1511058.28 | European Commission | UNIVERSIDADE NOVA DE LISBOA | Clinical | Policies for public health, disease control & community resilience |
| P23594 | 2022 | 2026 | a clinical research network to improve the management of Monkeypox virus disease | 12239998.98 | European Commission | INSTITUT NATIONAL DE LA SANTE ET DE LA RECHERCHE MEDICALE | Clinical \| Non-Clinical | Pathogen: natural history, transmission and diagnostics \| Clinical characterisation and management \| Therapeutics research, development and implementation \| Policies for public health, disease control & community resilience |
| P24916 | 2022 | 2023 | SE0572: Developing capability for detection of Monkeypox exposure in animals - SE0572 | 119388.6 | Department for Environment, Food and Rural Affairs (DEFRA) | APHA (Animal and Plant Health Agency) | Non-Clinical | Pathogen: natural history, transmission and diagnostics \| Animal and environmental research and research on diseases vectors |
| P24924 | 2023 | Unspecified | BioNTech - mpox | 90000000 | Coalition for Epidemic Preparedness Innovations (CEPI) | BioNtech | Clinical | Vaccines research, development and implementation |
| P25385 | 2022 | 2023 | Placebo-controlled randomised trial of tecovirimat in non-hospitalised Monkeypox patients (PLATINUM) | 1230477.65 | Department of Health and Social Care / National Institute for Health and Care Research (DHSC-NIHR) | University of Oxford | Clinical | Therapeutics research, development and implementation |
| P25754 | 2023 | Unspecified | Investigating Immunogenicity of Mpox and Mpox vaccine Imvamune in people living with HIV | 375198.56 | Canadian Institutes of Health Research (CIHR) | Research Institute of the McGill University Health Centre | Clinical | Pathogen: natural history, transmission and diagnostics \| Vaccines research, development and implementation |
| P25761 | 2023 | Unspecified | Lessons Learned from Engage Montréal: Study End Meeting | 4412.13 | Canadian Institutes of Health Research (CIHR) | Research Institute of the McGill University Health Centre | Not applicable | Policies for public health, disease control & community resilience |
| P25882 | 2023 | Unspecified | Development and Commercialization of a Safe and Effective Mpox Subunit Vaccine with Global Impact | 73558.84 | Canadian Institutes of Health Research (CIHR) | University of British Columbia | Unspecified | Vaccines research, development and implementation |
| P25952 | 2023 | Unspecified | Learning from mpox: Community-Based Mixed Methods Research to Support Intersectional and Stigma-Informed Approaches to Pandemic Preparedness for Gay, Bisexual, Queer, and Other Men who Have Sex with Men in Canada | 375198.56 | Canadian Institutes of Health Research (CIHR) | University of Toronto | Clinical \| Non-Clinical | Infection prevention and control \| Policies for public health, disease control & community resilience |
| P25960 | 2023 | Unspecified | Pre-exposure mpox vaccination campaigns aimed at GBTMSM+ and sex work communities: What worked in 2022 and what can we learn to strengthen future targeted vaccination campaigns to stigmatized communities in infodemic times? | 374950.93 | Canadian Institutes of Health Research (CIHR) | University of British Columbia | Non-Clinical | Infection prevention and control \| Policies for public health, disease control & community resilience |
| P26034 | 2023 | Unspecified | Optimizing Mpox surveillance strategies and preventing epidemic resurgence: a three-province mathematical modeling study | 375198.56 | Canadian Institutes of Health Research (CIHR) | B.C. Centre for Disease Control (Vancouver) | Non-Clinical | Epidemiological studies \| Infection prevention and control \| Policies for public health, disease control & community resilience |
| P26083 | 2022 | Unspecified | Preclinical Models for Monkeypox Virus Infection and Therapeutic Development | 76662.06 | Canadian Institutes of Health Research (CIHR) | University of Saskatchewan | Non-Clinical | Pathogen: natural history, transmission and diagnostics |
| P26085 | 2023 | Unspecified | Investigation of Mpox virus spillover and spillback at the human-animal interface in the Democratic Republic of Congo | 375198.56 | Canadian Institutes of Health Research (CIHR) | University of Saskatchewan | Non-Clinical | Animal and environmental research and research on diseases vectors \| Infection prevention and control |
| P26087 | 2023 | Unspecified | Strengthening global and regional health security through surveillance of emerging Poxviruses in humans, domestic and peri-domestic animals, and wildlife | 375198.56 | Canadian Institutes of Health Research (CIHR) | Dalhousie University (Nova Scotia) | Non-Clinical | Animal and environmental research and research on diseases vectors \| Policies for public health, disease control & community resilience |
| P26108 | 2022 | Unspecified | A prospective and retrospective multi-center, cohort study for clinical, virologic and immunologic characterization of monkeypox virus clade IIb by the International Monkeypox Response Consortium (IMREC) | 1945683.1 | Canadian Institutes of Health Research (CIHR) | University of Manitoba | Not applicable | Epidemiological studies \| Vaccines research, development and implementation |
| P26109 | 2022 | Unspecified | Characterization of monkeypox virus circulation and transmission from wildlife to humans in Africa and identification of wildlife species at elevated risk for infection in Canada | 574965.45 | Canadian Institutes of Health Research (CIHR) | University of Manitoba | Non-Clinical | Animal and environmental research and research on diseases vectors |
| P26126 | 2023 | Unspecified | Assessing mpox virus susceptibility, transmission, host immune responses, and virus evolution in key Canadian livestock species | 373697.77 | Canadian Institutes of Health Research (CIHR) | Vaccine and Infectious Disease Organization (Sask.) | Non-Clinical | Pathogen: natural history, transmission and diagnostics \| Animal and environmental research and research on diseases vectors \| Infection prevention and control |
| P26220 | 2023 | Unspecified | SMART (Smallpox vaccine for Mpox Post-Exposure Prophylaxis: A Cluster Randomized Controlled Trial) | 375198.56 | Canadian Institutes of Health Research (CIHR) | McMaster University | Clinical | Vaccines research, development and implementation |
| P26335 | 2023 | Unspecified | Understanding susceptibility and permissiveness to mpox virus across diverse mammalian species | 346683.47 | Canadian Institutes of Health Research (CIHR) | McMaster University | Non-Clinical | Pathogen: natural history, transmission and diagnostics \| Animal and environmental research and research on diseases vectors |
| P26342 | 2023 | Unspecified | Mpox exposure and transmission at the human-animal interface; a One Health approach to viral ecology | 374453.42 | Canadian Institutes of Health Research (CIHR) | Sunnybrook Research Institute (Toronto, Ontario) | Non-Clinical | Animal and environmental research and research on diseases vectors \| Infection prevention and control |
| P26362 | 2023 | Unspecified | Epidemiological modelling of behavioural impact on Mpox mitigation strategies | 309163.62 | Canadian Institutes of Health Research (CIHR) | Centre de recherche en santé publique (Montreal, Quebec) | Non-Clinical | Epidemiological studies |
| P26384 | 2023 | Unspecified | Using synthetic virology approaches to elucidate novel virus-host interactions and develop targeted therapeutics | 2222.03 | Canadian Institutes of Health Research (CIHR) | University of Alberta | Non-Clinical | Pathogen: natural history, transmission and diagnostics |
| P26507 | 2023 | Unspecified | Impact of host responses on mpox pathogenesis and tecovirimat efficacy in the Collaborative Cross mouse model of genetic diversity | 375198.56 | Canadian Institutes of Health Research (CIHR) | University of Saskatchewan | Non-Clinical | Pathogen: natural history, transmission and diagnostics \| Clinical characterisation and management \| Therapeutics research, development and implementation |
| P26688 | 2022 | Unspecified | Canada-Africa Monkeypox Partnership (CAMP): Characterizing transmission dynamics and evaluating medical countermeasures to inform the clinical and public health response to Mpox | 2299861.81 | Canadian Institutes of Health Research (CIHR) | Unity Health Toronto | Unspecified | Animal and environmental research and research on diseases vectors \| Epidemiological studies \| Infection prevention and control |
| P26828 | 2023 | Unspecified | Modelling, predicting and risk assessment of mpox (monkeypox) and other (re)emerging zoonotic threats to inform decision-making and public health actions: mathematical, geospatial and machine learning approaches | 360190.62 | Canadian Institutes of Health Research (CIHR) | York University (Toronto, Ontario) | Non-Clinical | Epidemiological studies |
| P28329 | 2021 | 2023 | The Tropics. Poverty, forests and diseases. | 81508.65 | Swiss National Science Foundation (SNSF) | Aix-Marseille Université École d'économie AMSR UMR 7316 École Centrale Marseille | Non-Clinical | Animal and environmental research and research on diseases vectors \| Secondary impacts of disease, response & control measures |
| P29279 | 2020 | 2023 | AFRIPOX - A One Health approach of monkeypox in Central African Republic | Unspecified | Institut Pasteur International Network (IPIN) | N/A | Non-Clinical | Animal and environmental research and research on diseases vectors \| Epidemiological studies |
| P29292 | 2024 | 2026 | Immunogenicity of fractional subcutaneous booster vaccination against mpox: a non-inferiority study | Unspecified | Netherlands Organisation for Health Research and Development (ZonMW) | Leids Universitair Medisch Centrum | Unspecified | Vaccines research, development and implementation |
| P29293 | 2024 | 2025 | Longevity of orthopoxvirus-specific immune responses induced by infection or vaccinationinfectie | Unspecified | Netherlands Organisation for Health Research and Development (ZonMW) | Erasmus Medisch Centrum | Non-Clinical | Pathogen: natural history, transmission and diagnostics \| Vaccines research, development and implementation |
| P29691 | 2024 | 2024 | Data preparedness of the Amsterdam Cohort Studies | Unspecified | Netherlands Organisation for Health Research and Development (ZonMW) | GGD Amsterdam | Non-Clinical | Epidemiological studies |
| P29784 | 2024 | Unspecified | Strengthening global and regional health security through surveillance of emerging Poxviruses in humans, domestic and peri-domestic animals, and wildlife | 850000 | African Institute for Mathematical Sciences \| Canadian Institutes of Health Research (CIHR) \| Research Nova Scotia | Dalhousie University | Non-Clinical | Animal and environmental research and research on diseases vectors \| Epidemiological studies |
| P29940 | 2022 | Unspecified | Study of the persistence of the Monkeypox virus over time on different surfaces or materials, under different environmental conditions and validation of decontamination methods | 154492.97 | Agence nationale de recherche sur le sida et les hépatites virale [National Agency for AIDS Research] (ANRS) | CEA | Unspecified | Pathogen: natural history, transmission and diagnostics \| Infection prevention and control |
| P29941 | 2022 | Unspecified | Pathophysiology of Monkeypox and antiviral screening models | 195749.5 | Agence nationale de recherche sur le sida et les hépatites virale [National Agency for AIDS Research] (ANRS) | Université de Poitiers (UR155560 LITEC) | Unspecified | Clinical characterisation and management \| Therapeutics research, development and implementation |
| P29942 | 2022 | Unspecified | Characterizing the spreading dynamics of 2022 monkeypox outbreak in France to aid public health policies | 245033.71 | Agence nationale de recherche sur le sida et les hépatites virale [National Agency for AIDS Research] (ANRS) | Institut Pierre Louis d'Epidémiologie et de Santé Publique (UMR1136) | Unspecified | Epidemiological studies |
| P29943 | 2022 | Unspecified | Infection by Monkey pox (MPXV) and male genital tract | 331634.73 | Agence nationale de recherche sur le sida et les hépatites virale [National Agency for AIDS Research] (ANRS) | IRSET (U1085) | Unspecified | Clinical characterisation and management |
| P29944 | 2022 | Unspecified | Monkeypox social sciences: perception of risks, health measures and vaccination | 214425.55 | Agence nationale de recherche sur le sida et les hépatites virale [National Agency for AIDS Research] (ANRS) | Aix-Marseille Université (SESSTIM) | Non-Clinical | Policies for public health, disease control & community resilience |
| P29945 | 2023 | Unspecified | Monkeypox virus distribution, transmission, dynamics and immune response (MOVIDA): a substudy of the MOSAIC European Monkeypox observational cohort | 582101.71 | Agence nationale de recherche sur le sida et les hépatites virale [National Agency for AIDS Research] (ANRS) | Hôpital Pitié-Salpêtrière (U1136 iPLESP) | Clinical | Epidemiological studies \| Pathogen: natural history, transmission and diagnostics |
| P29946 | 2022 | Unspecified | One Health approach to document the extent of monkey pox viruses (MPXV) infections to evaluate frequency and risk for cross-species transmissions to humans in Africa | 407036.55 | Agence nationale de recherche sur le sida et les hépatites virale [National Agency for AIDS Research] (ANRS) | Universite de Montpellier (TransVIHMI) | Unspecified | Animal and environmental research and research on diseases vectors \| Epidemiological studies |
| P29947 | 2022 | Unspecified | Seroprevalence of Monkeypox infection in HIV-infected patients and PrEP users | Unspecified | Agence nationale de recherche sur le sida et les hépatites virale [National Agency for AIDS Research] (ANRS) | Hopital Pitié-Salpêtrière | Non-Clinical | Epidemiological studies |
| P29948 | 2022 | Unspecified | Preclinical evaluation of MVA as vaccine against monkeypox virus (MKPXV) experimental infection in cynomolgus macaques | 309699.91 | Agence nationale de recherche sur le sida et les hépatites virale [National Agency for AIDS Research] (ANRS) | Institut de Recherche Biomédical des Armées | Non-Clinical | Vaccines research, development and implementation |
| P29949 | 2022 | Unspecified | Is MonkeyPox virus in domestic animal population from North of West-Africa endemic? first statements | 155333.94 | Agence nationale de recherche sur le sida et les hépatites virale [National Agency for AIDS Research] (ANRS) | Institut Pasteur | Non-Clinical | Animal and environmental research and research on diseases vectors |
| P29956 | 2023 | Unspecified | UNITY Monkeypox Trial | 169984 | Agence nationale de recherche sur le sida et les hépatites virale [National Agency for AIDS Research] (ANRS) | Instituto Nacional de Infectologia Evandro Chagas | Unspecified | Unspecified |
| P30068 | 2024 | 2024 | International scientific conference on strategies for the prevention and management of Mpox in Africa and the Democratic Republic of the Congo (DRC) | 100052.84 | Wellcome Trust | African Field Epidemiology Network | Non-Clinical | Policies for public health, disease control & community resilience \| Health Systems Research |
| P30155 | 2023 | 2023 | Mpox virus (MPV) asymptomatic transmission study during the outbreak in 2022 | 26936.17 | State Secretariat for Education, Research, and Innovation SERI (Staatssekretariat für Bildung, Forschung und Innovation) | N/A | Unspecified | Epidemiological studies \| Clinical characterisation and management |
| P30243 | 2023 | 2025 | Clinical development of DIOS-HFVac3: A pre-clinically effective multivalent vaccine for Lassa fever virus, Marburg virus, and Sudan ebolavirus | 2223341.25 | UK Research and Innovation (UKRI) | DIOSYNVAX LTD. | Non-Clinical | Vaccines research, development and implementation |
| P32717 | 2024 | 2027 | Tackling and investigating the South-Kivu mpox outbreak (MBOTE-SK) | 2255759.24 | European & Developing Countries Clinical Trials Partnership (EDCTP) | INSTITUUT VOOR TROPISCHE GENEESKUNDE | Clinical \| Non-Clinical | Pathogen: natural history, transmission and diagnostics \| Epidemiological studies \| Clinical characterisation and management \| Vaccines research, development and implementation \| Policies for public health, disease control & community resilience |
| P32718 | 2024 | 2026 | Implementing wastewater and environmental surveillance for Mpox in Sub-Saharan Africa (ODIN-MPox) | 1504660.09 | European & Developing Countries Clinical Trials Partnership (EDCTP) | LUNDS UNIVERSITET | Non-Clinical | Pathogen: natural history, transmission and diagnostics \| Animal and environmental research and research on diseases vectors \| Epidemiological studies |
| P32719 | 2024 | 2027 | Impact of MPXV infection on pregnancy outcome and newborn health (PREGMPOX) | 1364625 | European & Developing Countries Clinical Trials Partnership (EDCTP) | UNIVERSITEIT ANTWERPEN | Non-Clinical | Pathogen: natural history, transmission and diagnostics \| Epidemiological studies \| Clinical characterisation and management \| Therapeutics research, development and implementation \| Vaccines research, development and implementation |
| P32720 | 2024 | 2026 | Deciphering host genetics and viral determinants of MPOX epidemiology in the Democratic Republic of Congo (DECIPHER-MPOX) | 1375542 | European & Developing Countries Clinical Trials Partnership (EDCTP) | National Health Laboratory Services | Non-Clinical | Pathogen: natural history, transmission and diagnostics \| Epidemiological studies \| Clinical characterisation and management |
| P32721 | 2023 | 2025 | Development of multi-valent COVID-19 and mpox mRNA vaccine candidates | 167669.17 | Ministry of health and welfare (Korea) | National Institute of Health (Korea) | Non-Clinical | Vaccines research, development and implementation |
| P32722 | 2023 | 2024 | Evaluation of protective effect of smallpox vaccines against monkeypox virus in monkey model | 1492436.09 | Ministry of health and welfare (Korea) | HK inno.N Corporation | Non-Clinical | Vaccines research, development and implementation |
| P32723 | 2022 | 2023 | Evaluation of cross-immunogenicity between smallpox vaccine strain and monkeypox virus | 230769.23 | Ministry of health and welfare (Korea) | National Institute of Health (Korea) | Non-Clinical | Pathogen: natural history, transmission and diagnostics \| Vaccines research, development and implementation |
| P32781 | 2021 | 2026 | Mechanism-based Targeting of the RNA Processing Machinery of SARS-CoV-2 | 584674 | National Institutes of Health (NIH) | UNIVERSITY OF TEXAS HLTH SCIENCE CENTER | Non-Clinical | Pathogen: natural history, transmission and diagnostics \| Therapeutics research, development and implementation |
| P32835 | 2020 | 2025 | Advancement of poxvirus inhibitor | 745122 | National Institutes of Health (NIH) | BOSTON UNIVERSITY MEDICAL CAMPUS | Non-Clinical | Therapeutics research, development and implementation |
| P32837 | 2024 | 2028 | Emerging and Re-Emerging Pathogens Research Training Program in DRC (EREP-RTP-DRC) | 251215 | National Institutes of Health (NIH) | INSTITUT NATIONAL / RECHERCHE BIOMEDICAL | Not applicable | Not applicable |
| P32843 | 2020 | 2025 | A randomized, placebo-controlled trial of the safety and efficacy of tecovirimat for the treatment of patients with monkeypox virus disease | 9824009 | National Institutes of Health (NIH) | LEIDOS BIOMEDICAL RESEARCH, INC. | Clinical | Therapeutics research, development and implementation |
| P33021 | 2024 | 2028 | Vertical transmission, pregnancy outcomes and treatment of Mpox virus infection in a translational pregnant macaque model | 741916 | National Institutes of Health (NIH) | UNIVERSITY OF WISCONSIN-MADISON | Non-Clinical | Pathogen: natural history, transmission and diagnostics \| Clinical characterisation and management \| Therapeutics research, development and implementation |
| P33195 | 2024 | 2025 | HIV and (Re)Emerging Viruses: Aligning Lessons Across Pandemics | 17700 | National Institutes of Health (NIH) | KEYSTONE SYMPOSIA | Not applicable | Not applicable |
| P33202 | 2024 | 2029 | New mechanisms governing skin tissue residency memory T cells | 757027 | National Institutes of Health (NIH) | WEILL MEDICAL COLL OF CORNELL UNIV | Non-Clinical | Pathogen: natural history, transmission and diagnostics |
| P33223 | 2023 | 2028 | The role of mTOR dysregulation in poxvirus infection | 678119 | National Institutes of Health (NIH) | NORTHWESTERN UNIVERSITY AT CHICAGO | Non-Clinical | Pathogen: natural history, transmission and diagnostics |
| P33227 | 2024 | 2029 | The Bacterial and Viral Bioinformatics Resource Center (BV-BRC) | 3600000 | National Institutes of Health (NIH) | UNIVERSITY OF CHICAGO | Not applicable | Pathogen: natural history, transmission and diagnostics \| Epidemiological studies |
| P33229 | 2023 | 2028 | Facility Management, Maintenance and Operation Core | 1493669 | National Institutes of Health (NIH) | COLORADO STATE UNIVERSITY | Not applicable | Infection prevention and control |
| P33231 | 2024 | 2029 | Development of novel antivirals against mpox (monkeypox) virus | 783602 | National Institutes of Health (NIH) | UNIVERSITY OF MINNESOTA | Non-Clinical | Therapeutics research, development and implementation |
| P33249 | 2023 | 2028 | Facility Management, Maintenance and Operation Core | 1338439 | National Institutes of Health (NIH) | COLORADO STATE UNIVERSITY | Not applicable | Infection prevention and control |
| P33296 | 2024 | 2029 | Discovery of Antivirals Targeting Mpox Virus | 782142 | National Institutes of Health (NIH) | EMORY UNIVERSITY | Non-Clinical | Therapeutics research, development and implementation |
| P33297 | 2023 | 2025 | Poxvirus-encoded noncanonical open reading frames | 221364 | National Institutes of Health (NIH) | TEXAS A&M AGRILIFE RESEARCH | Non-Clinical | Pathogen: natural history, transmission and diagnostics |
| P33299 | 2024 | 2026 | Epidemiological factors related to human monkeypox virus (MPOX) in men who have sex with men (MSM) in the United States | 48974 | National Institutes of Health (NIH) | JOHNS HOPKINS UNIVERSITY | Non-Clinical | Epidemiological studies \| Policies for public health, disease control & community resilience |
| P33300 | 2024 | 2025 | CLINICAL RESEARCH OPERATIONS AND MANAGEMENT SUPPORT (CROMS) FOR MPOX | 750000 | National Institutes of Health (NIH) | TECHNICAL RESOURCES INTERNATIONAL, INC. | Not applicable | Therapeutics research, development and implementation \| Vaccines research, development and implementation |
| P33301 | 2023 | 2027 | Understanding the Function of F13 as a Matrix Protein for Poxvirus Intracellular Envelopment | 385000 | National Institutes of Health (NIH) | UNIVERSITY OF ROCHESTER | Non-Clinical | Pathogen: natural history, transmission and diagnostics |
| P33303 | 2024 | 2025 | Uncovering the molecular underpinnings of C15, a potent orthopoxvirus virulence factor | 36803 | National Institutes of Health (NIH) | UNIVERSITY OF PENNSYLVANIA | Non-Clinical | Pathogen: natural history, transmission and diagnostics |
| P33304 | 2024 | 2028 | The B22 family of orthopoxvirus virulence factors: Investigating structure/function of these potent, multifaceted immunoevasins | 742230 | National Institutes of Health (NIH) | CHILDREN'S HOSP OF PHILADELPHIA | Non-Clinical | Pathogen: natural history, transmission and diagnostics |
| P33320 | 2024 | Unspecified | Panafpox-2: Provide new insights on human-to-human transmissions of Mpox in the Democratic Republic of Congo | 228329.94 | Agence nationale de recherche sur le sida et les hépatites virale [National Agency for AIDS Research] (ANRS) | Universite de Montpellier (TransVIHMI) | Non-Clinical | Epidemiological studies |
| P33321 | 2024 | Unspecified | MATERNAL AND INFANT SAFETY AND IMMUNOGENICITY IN A PHASE 3, OPEN-LABEL, RANDOMISED, VACCINE TRIAL OF A TWO-DOSE MPOX VACCINE (PregInPoxVac) | 1786688.72 | European & Developing Countries Clinical Trials Partnership (EDCTP) | UNIVERSITEIT ANTWERPEN - UANTWERPEN | Clinical | Vaccines research, development and implementation \| Policies for public health, disease control & community resilience |

*Note: Grants for which there was insufficient information to determine the study type were classified as ‘Unspecified’. Grants that did not support a specific research study but instead focused on implementation, infrastructure, or capacity-strengthening activities were classified as ‘Not applicable (N/A)’. More details can be found on*
