# Supplementary material for: Gaps in the global health research landscape for mpox: an analysis of research activities and existing evidence
Source: BMC Med. 2025 Sep 29;23:522. doi: 10.1186/s12916-025-04350-1 (PMC12482760; doi:10.1186/s12916-025-04350-1)
Supplement: Supplementary file 3 — Additional file 3: Fig. S1 Distribution of mpox grants and financial contributions by funding organisations [file 12916_2025_4350_MOESM3_ESM.docx]

# **Additional file 3: Fig. S1**. Distribution of mpox grants and financial contributions by funding organisations

**
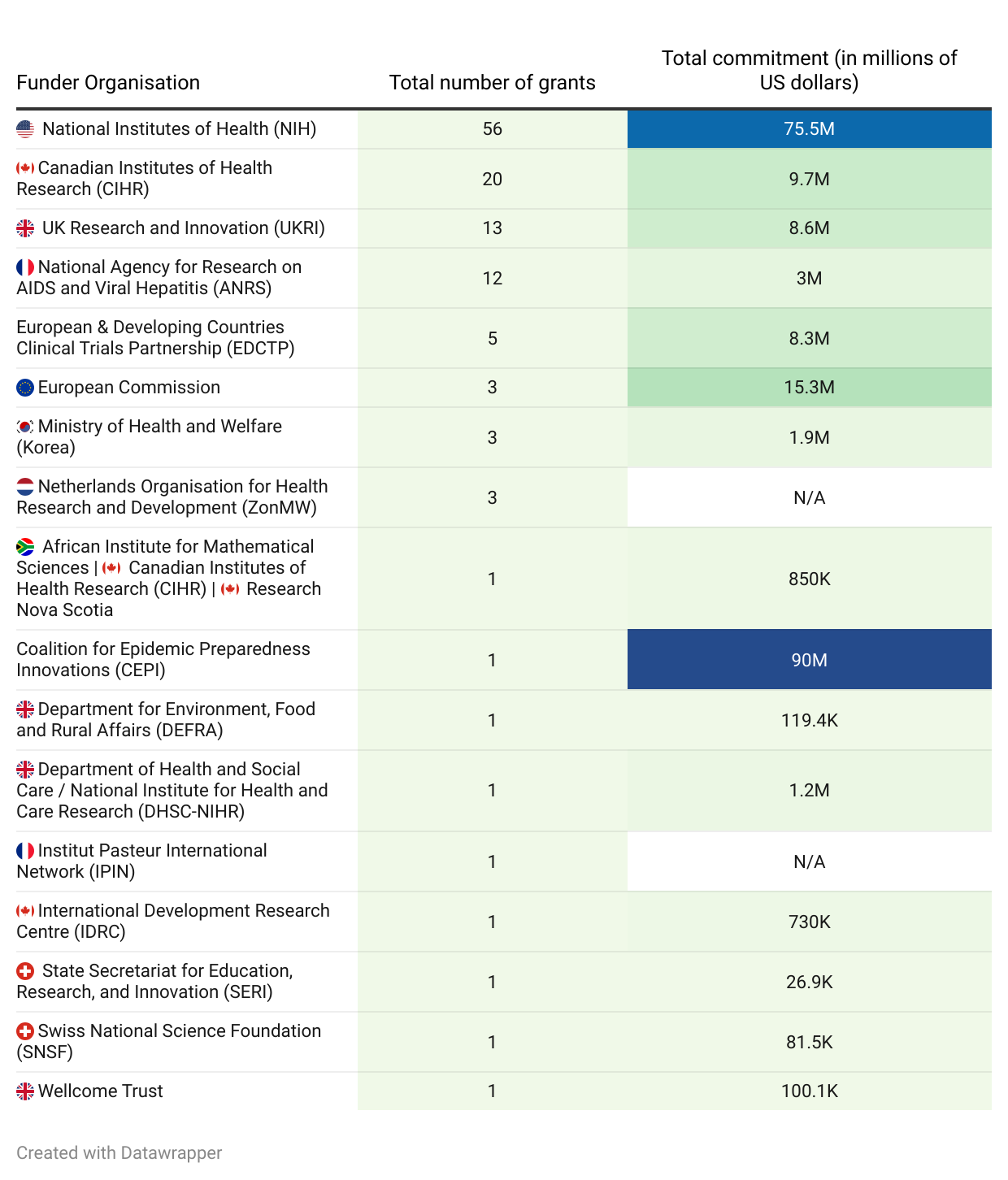
**

**Supplementary Table 2. Total number of mpox grants and known financial commitments by funding organisation**

Flags next to funder names represent the location where the funder is based. No flags are used for multinational organisations. Known funding amounts are shown in USD, and "N/A" indicates cases where funding information is unavailable. Many research funding organisations operate independently. However, funding mechanisms, sources, and flows vary across funders and can be interconnected. The analyses in this paper do not account for relationships between funding organisations unless they are explicitly listed as co-funders. As a result, contributions from some funders to other entities or funding schemes (when not explicitly stated) were not included in the total funding calculations, potentially leading to an underestimation of their contributions in some cases.
