# Supplementary material for: Gaps in the global health research landscape for mpox: an analysis of research activities and existing evidence
Source: BMC Med. 2025 Sep 29;23:522. doi: 10.1186/s12916-025-04350-1 (PMC12482760; doi:10.1186/s12916-025-04350-1)
Supplement: Supplementary file 4 — Additional file 4: Table S3 Distribution of interventions investigated in mpox clinical trials in PACTR, SANCTR and WHO ICTRP [file 12916_2025_4350_MOESM4_ESM.docx]

# **Additional file 4: Table S3**. Distribution of interventions investigated in mpox clinical trials in PACTR, SANCTR and WHO ICTRP

| **Clinical trial intervention type** | **Intervention** | **% of trials**  (N=21) | **Phases** | **Study population** | **Year registered** |
| --- | --- | --- | --- | --- | --- |
| **Therapeutics (n=21)** | Tecovirimat | 66.7% (n=14) | II (n= 4)  III (n= 7)  IV (n= 3) | Adults & children | 2021-2024 |
|  | VIG vaccinia immunoglobulin intravenous formulation | 9.5% (n=2) | II (n=2) | Adults | 2023-2024 |
|  | Cidofovir IV + Oral Probenecid | 4.8% (n=1) | II | Adults | 2024 |
|  | JBP-TOPOXX 3 capsules | 4.8% (n=1) | I | Adults | 2023 |
|  | Capsule 4 of edible fungi based on Ganoderma, Lentinula edodes, Trametes versicolor, Grifola frondosa | 4.8% (n=1) | II | Adults | 2022 |
|  | NIOCH-14 | 4.8% (n=1) | I | Adults | 2023 |
|  | Trifluridine | 4.8% (n=1) | II | Adults and children | 2024 |
| **Clinical trial intervention type** | **Intervention** | **% of trials**  (N=17) | **Phases** | **Study populations** | **Year registered** |
| **Vaccines (n=17)** | Modified Vaccinia Ankara-Bavarian Nordic (MVA-BN) Vaccine (JYNNEOS and or IMVANEX) | 52.9% (n= 9) | I (n= 1)  II (n=3)  IV (n=5) | Adults & children | 2022-2024 |
|  | LC16m8 Mpox Vaccine | 17.6% (n=3) | I  III  IV | Adults & children | 2022-2023 |
|  | VAC∆6 Vaccine | 11.8% (n=2) | I  II/III | Adults & children | 2023 |
|  | RNA-based multivalent vaccine candidate BNT166a | 5.9% (n=1) | I/II | Adults | 2023 |
|  | Smallpox vaccine (Dry cell cultured smallpox vaccine) | 5.9% (n=1) | II | Adults & children | 2022 |
|  | mRNA-1769 | 5.9% (n=1) | I/II | Adults | 2023 |
