# Supplementary material for: Gaps in the global health research landscape for mpox: an analysis of research activities and existing evidence
Source: BMC Med. 2025 Sep 29;23:522. doi: 10.1186/s12916-025-04350-1 (PMC12482760; doi:10.1186/s12916-025-04350-1)
Supplement: Supplementary file 5 — Additional file 5: Table S4 Systematic reviews included in the rapid research needs appraisals [file 12916_2025_4350_MOESM5_ESM.docx]

# **Additional file 5: Table S4.** Systematic reviews included in the Rapid Research Needs Appraisals

| **Bibliography** | **No. studies** | **Region** | **Clade** | **Domain** |
| --- | --- | --- | --- | --- |
| Abu-Hammad, O. et al (2023). Factors associated with geographic variations in the 2022 monkeypox outbreak; A systematic review New Microbes New Infect, 51, 101078 | 24 | Asia, Europe, North Americas | NR | 2. Transmission |
| Akter, F. et al (2023). Effect of prior immunisation with smallpox vaccine for protection against human Mpox: A systematic review Rev Med Virol, 33(4), e2444 | 6 | Africa, Europe, North Americas | NR | 3. Prophylaxis |
| Asaad, S. et al (2023). Cutaneous eruptions associated with monkeypox virus infection: A systematic review JAAD Int, 12, 179-181 | 33 | Australia, Europe, Latin Americas & the Caribbean, Middle East, North Americas | NR | 1. Clinical characterisation |
| Badenoch, J. B. et al (2022). Neurological and psychiatric presentations associated with human monkeypox virus infection: A systematic review and meta-analysis EClinicalMedicine, 52, 101644 | 19 | Europe, North Americas | II | 1. Clinical characterisation |
| Barboza, J. J. et al (2023). Virus Identification for Monkeypox in Human Seminal Fluid Samples: A Systematic Review Trop Med Infect Dis, 8(3), | 14 | Australia, Europe, Latin Americas & the Caribbean, Middle East, North Americas | NR | 2. Transmission |
| Benites-Zapata, V. A. et al (2022). Clinical features, hospitalisation and deaths associated with monkeypox: a systematic review and meta-analysis Ann Clin Microbiol Antimicrob, 21(1), 36 | 12 | Africa, Europe, Middle East, North Americas | NR | 1. Clinical characterisation |
| Chadaga, K. et al (2023). Application of Artificial Intelligence Techniques for Monkeypox: A Systematic Review Diagnostics (Basel), 13(5), | 34 | NR | NR | 4. Diagnostics |
| Chenchula, S. et al (2023). A systematic review to identify novel clinical characteristics of monkeypox virus infection and therapeutic and preventive strategies to combat the virus Arch Virol, 168(7), 195 | 21 | Europe, North Americas | NR | 1. Clinical characterisation, 5. Therapeutics |
| Deb, N. et al (2023). Neurological Manifestations of Coronavirus Disease 2019 and Mpox in Pediatric Patients and Their Management: A State-of-the-Art Systematic Review Pediatr Neurol, 146, 65-78 | 3 on mpox | Africa, Europe, North Americas | NR | 1. Clinical characterisation |
| Della Valle, L. et al (2023). Maternal and perinatal outcomes of pregnancies complicated by poxviruses infection: a systematic review and meta-analysis Italian Journal of Gynaecology and Obstetrics, 35(Supplement 1), 57 | 4 | Africa, Asia, Australia, Europe, Latin Americas & the Caribbean, North Americas | NR | 1. Clinical characterisation, 2. Transmission |
| DeWitt, M. E. et al (2022). Global monkeypox case hospitalisation rates: A rapid systematic review and meta-analysis EClinicalMedicine, 54, 101710 | 19 | Europe, North Americas | I, II | 1. Clinical characterisation |
| Du, M. et al (2023). Global Epidemiological Features of Human Monkeypox Cases and Their Associations With Social-Economic Level and International Travel Arrivals: A Systematic Review and Ecological Study Int J Public Health, 68, 1605426 | 78 | Europe, Latin Americas & the Caribbean, North Americas | I, II | 1. Clinical characterisation |
| El-Qushayri, A. E. et al (2023). Cardiovascular manifestations of monkeypox virus outbreak: An overview of the reported cases Heart and Lung, 59, 67-72 | 7 | Europe, North Americas | NR | 1. Clinical characterisation |
| Eslami, A. et al (2023). Mpox vaccination and treatment: a systematic review Journal of chemotherapy (Florence, Italy), , 1-25 | 13 | NR | NR | 3. Prophylaxis, 5. Therapeutics |
| Fox, T. et al (2023). Therapeutics for treating mpox in humans Cochrane Database Syst Rev, 3(3), Cd015769 | 3 | Africa, Europe, Latin Americas & the Caribbean, North Americas | NR | 5. Therapeutics |
| Gandhi, A. P. et al (2023). Ophthalmic Manifestations of the Monkeypox Virus: A Systematic Review and Meta-Analysis Pathogens, 12(3), | 12 | Africa, Australia, Europe, Middle East, North Americas | NR | 1. Clinical characterisation |
| Gandhi, A. P. et al (2023). Monkeypox Patients Living with HIV: A Systematic Review and Meta-Analysis of Geographic and Temporal Variations Epidemiologia (Basel), 4(3), 352-369 | 32 | Africa, Europe, North Americas | I, II | 1. Clinical characterisation, 2. Transmission |
| Gandhi, P. A. et al (2023). Oral manifestation of the monkeypox virus: a systematic review and meta-analysis EClinicalMedicine, 56, 101817 | 19 | Europe, North Americas | NR | 1. Clinical characterisation |
| Ghazy, R. M. et al (2023). Systematic Review on the Efficacy, Effectiveness, Safety, and Immunogenicity of Monkeypox Vaccine Vaccines, 11(11), 1708 | 41 | Africa, Asia, Europe, Latin Americas & the Caribbean, North Americas | NR | 3. Prophylaxis |
| Hallo-Carrasco, A. et al (2023). Pain Associated With Monkeypox Virus: A Rapid Review Cureus, 15(2), e34697 | 15 | NR | NR | 1. Clinical characterisation, 5. Therapeutics, 6. Supportive care |
| Hatami, H. et al (2023). Demographic, Epidemiologic, and Clinical Characteristics of Human Monkeypox Disease Pre- and Post-2022 Outbreaks: A Systematic Review and Meta-Analysis Biomedicines, 11(3), | 98 | Asia, Europe, North Americas | II | 1. Clinical characterisation |
| Islam, M. A. et al (2022). An Estimation of Five-decade Long Monkeypox Case Fatality Rate: Systematic Review and Meta-analysis Journal of Pure and Applied Microbiology, 16, 3036-3047 | 17 | Africa, Europe | NR | 1. Clinical characterisation |
| Jahromi, A. S. et al (2023). Global knowledge and attitudes towards mpox (monkeypox) among healthcare workers: a systematic review and meta-analysis Int Health, , | 22 | Africa, Asia, Europe, North Americas | NR | 7. Social sciences |
| Kandeel, M. et al (2023). Efficacy of the modified vaccinia Ankara virus vaccine and the replication-competent vaccine ACAM2000 in monkeypox prevention International immunopharmacology, 119, 110206 | 8 | NR | NR | 3. Prophylaxis |
| Kim, H. et al (2023). Viral load dynamics and shedding kinetics of mpox infection: a systematic review and meta-analysis J Travel Med, 30(5), | 19 | Asia, Australia, Europe, Middle East, North Americas | NR | 4. Diagnostics |
| Kumar, R. et al (2022). A Systematic Review of 5110 Cases of Monkeypox: What Has Changed Between 1970 and 2022? Cureus, 14(10), e30841 | 63 | Asia, Australia, Europe, Latin Americas & the Caribbean, Middle East, North Americas | NR | 1. Clinical characterisation, 2. Transmission |
| León-Figueroa, D. A. et al (2022). Detection of Monkeypox Virus according to The Collection Site of Samples from Confirmed Cases: A Systematic Review Trop Med Infect Dis, 8(1), | 65 | Africa, Europe, Middle East, North Americas | NR | 4. Diagnostics |
| León-Figueroa, D. A. et al (2024). Prevalence of intentions to receive monkeypox vaccine. A systematic review and meta-analysis BMC Public Health, 24(1), 35 | 29 | Africa, Asia, Australia, Europe, Latin Americas & the Caribbean, Middle East, North Americas | NR | 7. Social sciences |
| Li, P. et al (2023). Clinical Features, Antiviral Treatment, and Patient Outcomes: A Systematic Review and Comparative Analysis of the Previous and the 2022 Mpox Outbreaks J Infect Dis, 228(4), 391-401 | 73 | Africa | NR | 1. Clinical characterisation, 5. Therapeutics |
| Liu, Q. et al (2023). Clinical Characteristics of Human Mpox (Monkeypox) in 2022: A Systematic Review and Meta-Analysis Pathogens, 12(1), | 77 | Asia, Australia, Europe, North Americas | NR | 1. Clinical characterisation |
| Malone, S. M. et al (2023). Safety and Efficacy of Post-Eradication Smallpox Vaccine as an Mpox Vaccine: A Systematic Review with Meta-Analysis Int J Environ Res Public Health, 20(4), | 10 | Europe, Latin Americas & the Caribbean, North Americas | NR | 3. Prophylaxis |
| Martins-Filho, P. R. et al (2022). Polymerase chain reaction positivity and cycle threshold values in biological samples from patients with monkeypox: A meta-analysis Travel Med Infect Dis, 50, 102448 | 8 | Europe, Middle East | NR | 4. Diagnostics |
| Mektebi, A. et al (2024). Mpox vaccine acceptance among healthcare workers: a systematic review and meta-analysis BMC Public Health, 24(1), 4 | 10 | Africa, Asia, Europe, Middle East, North Americas | NR | 7. Social sciences |
| Moawad, M. H. et al (2023). Attitudes towards Receiving Monkeypox Vaccination: A Systematic Review and Meta-Analysis Vaccines (Basel), 11(12), | 30 | Africa, Asia, Europe, Middle East, North Americas | NR | 7. Social sciences |
| Nave, L. et al (2023). Immunogenicity and Safety of Modified Vaccinia Ankara (MVA) Vaccine-A Systematic Review and Meta-Analysis of Randomized Controlled Trials Vaccines (Basel), 11(9), | 8 | NR | NR | 3. Prophylaxis |
| Nilasari, H. et al (2024). The evolving monkeypox outbreak amongst homosexual and bisexual transmission: A systematic review Journal of Pakistan Association of Dermatologists, 34(1), 243-254 | 10 | Asia, Australia, Europe | NR | 2. Transmission |
| Núñez-Cortés, R. et al (2023). Risk profile and mode of transmission of Mpox: A rapid review and individual patient data meta-analysis of case studies Rev Med Virol, 33(2), e2410 | 62 | Asia, Australia, Europe, Middle East, North Americas | NR | 2. Transmission |
| Okoli, G. N. et al (2023). A global systematic evidence review with meta-analysis of the epidemiological characteristics of the 2022 Mpox outbreaks Infection | 66 | Africa, Asia, Europe, Latin Americas & the Caribbean, Middle East | NR | 1. Clinical characterisation, 2. Transmission |
| Okoli, G. N. et al (2023). Comparative evaluation of the clinical presentation and epidemiology of the 2022 and previous Mpox outbreaks: a rapid review and meta-analysis Infect Dis (Lond), 55(7), 490-508 | 79 | Asia, Australia, Europe, Latin Americas & the Caribbean, Middle East, North Americas | II | 1. Clinical characterisation |
| Ortiz-Saavedra, B. et al (2023). Epidemiologic Situation of HIV and Monkeypox Coinfection: A Systematic Review Vaccines (Basel), 11(2), | 53 | Asia, Australia, Europe, Middle East, North Americas | NR | 1. Clinical characterisation |
| Pinto, P. et al (2023). Mpox Person-to-Person Transmission-Where Have We Got So Far? A Systematic Review Viruses, 15(5), | 15 | Asia, Europe, North Americas | II | 2. Transmission |
| Pourriyahi, H. et al (2023). A systematic review and clinical atlas on mucocutaneous presentations of the current monkeypox outbreak: With a comprehensive approach to all dermatologic and nondermatologic aspects of the new and previous monkeypox outbreaks J Med Virol, 95(2), e28230 | 46 | Asia, Australia, Europe, North Americas | II | 1. Clinical characterisation, 2. Transmission |
| Ramakrishnan, R. et al (2024). Mpox gastrointestinal manifestations: a systematic review BMJ Open Gastroenterol, 11(1), | 33 | Africa, Europe, Middle East, North Americas | NR | 1. Clinical characterisation |
| Rani, I. et al (2023). Prevalence of mpox viral DNA in cutaneous specimens of monkeypox-infected patients: a systematic review and meta-analysis Front Cell Infect Microbiol, 13, 1179885 | 14 | Europe, North Americas | NR | 4. Diagnostics |
| Rani, I. et al (2023). Viral Loads in Skin Samples of Patients with Monkeypox Virus Infection: A Systematic Review and Meta-Analysis Viruses, 15(6), | 9 | Europe, North Americas | NR | 4. Diagnostics |
| Reda, A. et al (2023). Monkeypox viral detection in semen specimens of confirmed cases: A systematic review and meta-analysis J Med Virol, 95(1), e28250 | 14 | Europe, North Americas | NR | 2. Transmission |
| Rojas-Carabali, W. et al (2023). Spectrum of ophthalmic manifestations in monkeypox virus infection worldwide: Systematic review and meta-analysis Heliyon, 9(8), e18561 | 60 | Africa, Australia, Europe, Latin Americas & the Caribbean, North Americas | I,IIa, IIb | 1. Clinical characterisation, 5. Therapeutics |
| Satapathy, P. et al (2022). Potentially Asymptomatic Infection of Monkeypox Virus: A Systematic Review and Meta-Analysis Vaccines (Basel), 10(12), | 14 | Europe | NR | 1. Clinical characterisation |
| Sayad, R. et al (2023). Can the current monkeypox affect the heart? A systematic review of case series and case report BMC Cardiovasc Disord, 23(1), 328 | 9 | Europe, Latin Americas & the Caribbean, North Americas | NR | 1. Clinical characterisation |
| Shah, J. et al (2023). Otolaryngologic manifestations among MPOX patients: A systematic review and meta-analysis Am J Otolaryngol, 44(6), 103991 | 38 | Africa, Asia, Europe, North Americas | NR | 1. Clinical characterisation |
| Shamim, M. A. et al (2023). The use of antivirals in the treatment of human monkeypox outbreaks: a systematic review Int J Infect Dis, 127, 150-161 | 18 | Africa, Europe, North Americas | NR | 5. Therapeutics |
| Sharif, N. et al (2023). Molecular epidemiology, transmission and clinical features of 2022-mpox outbreak: A systematic review Health Sci Rep, 6(10), e1603 | 45 | Europe, Latin Americas & the Caribbean, North Americas | NR | 2. Transmission |
| Sharma, A. et al (2023). Monkeypox epidemiology, clinical presentation, and transmission: a systematic review Int J Emerg Med, 16(1), 20 | 26 | Asia, Australia, Europe, Latin Americas & the Caribbean, North Americas | NR | 2. Transmission |
| Shin, H. et al (2023). Comparison of clinical manifestations in mpox patients living with HIV versus without HIV: A systematic review and meta-analysis Journal of Medical Virology, 95(4), e28713 | 99 | Asia, Australia, Europe, Latin Americas & the Caribbean, North Americas | NR | 1. Clinical characterisation, 2. Transmission |
| Simadibrata, D. M. et al (2023). Gastrointestinal Symptoms of Monkeypox Infection: A systematic review and meta-analysis J Med Virol, 95(4), e28709 | 31 | Africa, Australia, Europe, Latin Americas & the Caribbean, Middle East | I, IIa, IIb | 1. Clinical characterisation |
| Su, S. et al (2024). Integrated Network Analysis of Symptom Clusters Across Monkeypox Epidemics From 1970 to 2023: Systematic Review and Meta-Analysis JMIR public health and surveillance, 10, e49285 | 51 | Africa, Europe, Latin Americas & the Caribbean, Middle East | I, IIa, IIb | 1. Clinical characterisation |
| Suvvari, T. K. et al (2023). A meta-analysis and mapping of global mpox infection among children and adolescents Rev Med Virol, 33(5), e2472 | 25 | Europe, North Americas | NR | 1. Clinical characterisation |
| Ulloque-Badaracco, J. R. et al (2022). Acceptance towards Monkeypox Vaccination: A Systematic Review and Meta-Analysis Pathogens, 11(11), | 22 | Africa, Asia, Europe, Middle East, North Americas | NR | 7. Social sciences |
| Xu, M. et al (2023). Real-world effectiveness of monkeypox vaccines: a systematic review J Travel Med, 30(5), | 9 | Europe, Middle East, North Americas | NR | 3. Prophylaxis |
| Yon, H. et al (2023). Clinical manifestations of human Mpox infection: A systematic review and meta-analysis Rev Med Virol, 33(4), e2446 | 26 | Africa, Australia, Europe, Latin Americas & the Caribbean, Middle East, North Americas | NR | 1. Clinical characterisation |

Abbreviations: NR=not reported
