# Supplementary material for: Gaps in the global health research landscape for mpox: an analysis of research activities and existing evidence
Source: BMC Med. 2025 Sep 29;23:522. doi: 10.1186/s12916-025-04350-1 (PMC12482760; doi:10.1186/s12916-025-04350-1)
Supplement: Supplementary file 6 — Additional file 6: Table S5 Primary studies included in the rapid research needs appraisals [file 12916_2025_4350_MOESM6_ESM.docx]

# **Additional file 6: Table S5**. Primary studies included in the Rapid Research Needs Appraisals

| **Bibliography** | **Setting** | **Study design** | **Control group** | **Clade** | **Domain** | **Populations*** |
| --- | --- | --- | --- | --- | --- | --- |
| Abd ElHafeez, S. et al (2023). Assessing disparities in medical students' knowledge and attitude about monkeypox: a cross-sectional study of 27 countries across three continents Frontiers in public health, 11, 1192542 | Algeria, Bahrain, Bangladesh, Brazil, Bulgaria, China, Egypt, Ethiopia, Ghana, Greece, India, Iraq, Ireland, Italy, Jordan, Mexico, Morocco, Nepal, New Zealand, Pakistan, Palestine, Philippines, Poland, Qatar, Romania, Saudi Arabia, Senegal, South Africa, Sudan, Syria, Tanzania, Tunisia, Ukraine, UK, USA, Yemen | Obs | No | NR | 7. Social sciences | Adults 18-65, Students |
| Abu-Farha, R. K. et al (2023). Public Knowledge and Perceptions about the Emerging Human Mpox in Jordan: A Cross-Sectional Study Tropical Medicine and Infectious Disease, 8(1), 41 | Jordan | Obs | No | NR | 7. Social sciences | Adults, Older adults |
| Aden, T. A. et al (2022). Rapid Diagnostic Testing for Response to the Monkeypox Outbreak - Laboratory Response Network, United States, May 17-June 30, 2022 MMWR Morb Mortal Wkly Rep, 71(28), 904-907 | USA | Obs | No | NR | 4. Diagnostics | NR |
| Agroia, H. et al (2023). Monkeypox (Mpox) Vaccine Hesitancy Among Mpox Cases: A Qualitative Study Health promotion practice, 15248399231215054 | USA | Obs | No | NR | 7. Social sciences | Adults, Mpox patients |
| Ahmed, S. K. et al (2023). Knowledge, Attitude and Worry in the Kurdistan Region of Iraq during the Mpox (Monkeypox) Outbreak in 2022: An Online Cross-Sectional Study Vaccines (Basel), 11(3), | Iraq | Obs | No | NR | 7. Social sciences | Adults |
| Ahmed, S. K. et al (2023). Knowledge, Attitudes, and Willingness of Healthcare Workers in Iraq's Kurdistan Region to Vaccinate against Human Monkeypox: A Nationwide Cross-Sectional Study Vaccines, 11(12), 1734 | Iraq | Obs | No | NR | 7. Social sciences | Adults, Healthcare workers |
| Ahmed, S. et al (2022). The Mpox Disease: Awareness and Apprehensions among the Medical Undergraduates and Nursing Staff Journal of Pure and Applied Microbiology, 16, 3155-3167 | India | Obs | No | NR | 7. Social sciences | Adults, Healthcare workers, Students |
| Ajayi, P. O. et al (2023). Knowledge and awareness of human mpox infection among healthcare workers: A cross-sectional study in southwestern Nigeria Asian Pacific Journal of Tropical Medicine, 16(6), 245-252 | Nigeria | Obs | No | NR | 7. Social sciences | Adults |
| Alarifi, A. M. et al (2023). Are Saudi Healthcare Workers Willing to Receive the Monkeypox Virus Vaccine? Evidence from a Descriptive-Baseline Survey Tropical Medicine and Infectious Disease, 8(8), 396 | Saudi Arabia | Obs | No | NR | 7. Social sciences | Adults, Healthcare workers |
| Aldred, B. et al (2024). Early Tecovirimat Treatment for Mpox Disease Among People With HIV JAMA Internal Medicine, 184(3), 275-279 | USA | Int | Yes | NR | 5. Therapeutics | Adults, PLHIV |
| Algarate, S. et al (2023). Usefulness of Non-Skin Samples in the PCR Diagnosis of Mpox (Monkeypox) Viruses, 15(5), 1107 | Spain | Obs | No | NR | 4. Diagnostics | Adolescents, Adults |
| Alhasan, K. et al (2023). Mpox Perceptions and Vaccine Advocacy among the Healthcare Workers of Solid Organ Transplant Centers: A Multicenter, Cross-Sectional Survey in Saudi Arabia Healthcare (Switzerland), 11(4), | Saudi Arabia | Obs | No | NR | 7. Social sciences | Adults, Healthcare workers |
| Alhummayani, N. M. et al (2023). Awareness and Knowledge of the General Population About Monkeypox Disease in Riyadh, Saudi Arabia Cureus, 15(12), e50171 | Saudi Arabia | Obs | No | NR | 7. Social sciences | Adults |
| Aljahdali, S. H. et al (2023). Knowledge and Attitude of Human Monkeypox Viral Infection Among Healthcare Practitioners and Students in Saudi Arabia: A Cross-Sectional Study Cureus, 15(9), e45092 | Saudi Arabia | Obs | No | NR | 7. Social sciences | Adults |
| Alkalash, S. H. et al (2023). Evaluation of human monkeypox knowledge and beliefs regarding emerging viral infections among healthcare workers Int J Emerg Med, 16(1), 75 | Egypt | Obs | No | NR | 7. Social sciences | Adults, Healthcare workers |
| Allan-Blitz, L. T. et al (2023). Laboratory validation and clinical performance of a saliva-based test for monkeypox virus Journal of Medical Virology, 95(1), e28191 | USA | Obs | No | I, II | 4. Diagnostics | Adults |
| Allan-Blitz, L. T. et al (2023). Addressing mpox at a Frontline Community Health Center: Lessons for the Next Outbreak Public Health Reports, | USA | Obs | No | NR | 7. Social sciences | NR, at risk group |
| Allard, R. et al (2024). Breakthrough cases of mpox: One-dose vaccination is associated with milder clinical manifestations Journal of Infection and Public Health, 17(4), 676-680 | Canada | Obs | No | NR | 3. Prophylaxis | Young Children (1-5 years), Children (5-13 years), Adolescents, Adults, Older adults, PLHIV |
| Al-Mustapha, A. I. et al (2023). A cross-sectional survey of public knowledge of the monkeypox disease in Nigeria BMC public health, 23(1), 591 | Nigeria | Obs | No | NR | 7. Social sciences | Adults |
| Alqthami, O. A. et al (2023). Assessing the Anxiety and Knowledge about Monkeypox Virus: A Cross-Sectional Cohort Study Cureus, 15(10), e47806 | Saudi Arabia | Obs | No | NR | 7. Social sciences | Adults, Older adults |
| Alrasheedy, A. A. et al (2023). Knowledge of Community Pharmacists in Saudi Arabia Regarding Human Monkeypox, Its Management, Prevention, and Vaccination: Findings and Implications Vaccines, 11(4), 878 | Saudi Arabia | Obs | No | NR | 7. Social sciences | Adults, Healthcare workers |
| Alshahrani, N. Z. et al (2022). Knowledge and Attitude Regarding Monkeypox Virus among Physicians in Saudi Arabia: A Cross-Sectional Study Vaccines, 10(12), 2099 | Saudi Arabia | Obs | No | NR | 7. Social sciences | Adults, Healthcare workers |
| Alshahrani, N. Z. et al (2022). Assessment of Knowledge of Monkeypox Viral Infection among the General Population in Saudi Arabia Pathogens, 11(8), 904 | Saudi Arabia | Obs | No | NR | 7. Social sciences | Adults |
| Alshehri, S. et al (2023). Vaccine conspiracy association with higher COVID-19 vaccination side effects and negative attitude towards booster COVID-19, influenza and monkeypox vaccines: A pilot study in Saudi Universities Human Vaccines and Immunotherapeutics, 19(3), 2275962 | Saudi Arabia | Obs | No | NR | 7. Social sciences | Adults, Students, academics |
| Amer, F. A. et al (2024). Grasping knowledge, attitude, and perception towards monkeypox among healthcare workers and medical students: an Egyptian cross-sectional study Frontiers in Cellular and Infection Microbiology, 14, 1339352 | Egypt | Obs | No | NR | 7. Social sciences | Adults, Students |
| Andersen, E. W. et al (2024). Mpox Awareness, Risk Reduction, and Vaccine Acceptance among People with HIV in Washington, DC Pathogens, 13(2), 124 | USA | Obs | No | NR | 7. Social sciences | Adults, PLHIV |
| Anderson, M. et al (2024). Development and Validation of Three Automated High-Throughput Molecular Tests to Detect Monkeypox Virus Infections Journal of Infectious Diseases, 229(Supplement 2), S137-S143 | NR | Obs | No | NR | 4. Diagnostics | NR |
| Anoop, V. S. et al (2023). Public discourse and sentiment during Mpox outbreak: an analysis using natural language processing Public Health, 218, 114-120 | NR | Obs | No | NR | 7. Social sciences | NR |
| Araoz-Salinas, J. M. et al (2023). Perceptions and Intention to Get Vaccinated against Mpox among the LGBTIQ+ Community during the 2022 Outbreak: A Cross-Sectional Study in Peru Vaccines, 11(5), 1008 | Peru | Obs | No | NR | 7. Social sciences | NR |
| Arguelles, M. E. A. et al (2023). Detecting, Quantifying, and Isolating Monkeypox Virus in Suspected Cases, Spain Emerging Infectious Diseases, 29(7), 1465-1469 | Spain | Obs | No | NR | 4. Diagnostics | Adults, MSM, People with other specific comorbidities |
| Asif, S. et al (2024). CGO-ensemble: Chaos game optimization algorithm-based fusion of deep neural networks for accurate Mpox detection Neural Networks, 173, 106183 | NR | Obs | No | NR | 4. Diagnostics | NR |
| Asquith, W. et al (2024). Characterizing the acute antibody response of monkeypox and MVA-BN vaccine following an Australian outbreak Journal of Medical Virology, 96(1), e29407 | Australia | Obs | No | NR | 3. Prophylaxis | Adults, Older adults, Healthcare workers |
| Awoyomi, O. J. et al (2023). Mpox in Nigeria: Perceptions and knowledge of the disease among critical stakeholders-Global public health consequences PLoS One, 18(3), e0283571 | Nigeria | Obs | No | NR | 7. Social sciences | Adults, Healthcare workers, Students, Academics |
| Aynalem, Z. B. et al (2024). Knowledge, Attitude and Associated Factors of Monkeypox Infection Among Healthcare Workers in Injibara General Hospital, Northwest Ethiopia Journal of Multidisciplinary Healthcare, 17, 1159-1173 | Ethiopia | Obs | No | NR | 7. Social sciences | Adults, Healthcare workers |
| Bakshi, A. et al (2024). Pivoting COVID-19 Resources for an Equitable Mpox Vaccine Response in Louisiana AJPM Focus, 3(3), | USA | Obs | No | NR | 7. Social sciences | Adults, Healthcare workers |
| Bates, B. R. et al (2022). Knowledge, attitudes, and practices towards monkeypox during the 2022 outbreak: An online cross-sectional survey among clinicians in Ohio, USA Journal of Infection and Public Health, 15(12), 1459-1465 | USA | Obs | No | NR | 7. Social sciences | Adults, Healthcare workers |
| Batty, M. et al (2023). Laboratory assessment of a multi-target assay for the rapid detection of viruses causing vesicular diseases Journal of Clinical Virology, 165, 105525 | Australia | Obs | No | II | 4. Diagnostics | NR |
| Bautista, G. J. et al (2024). Reducing Vaccination Disparities During a National Emergency Response: The US Mpox Vaccine Equity Pilot Program Journal of public health management and practice: JPHMP, 30(1), 122-129 | USA | Obs | No | NR | 7. Social sciences | Adults, minorities |
| Bengesi, S. et al (2023). A Machine Learning-Sentiment Analysis on Monkeypox Outbreak: An Extensive Dataset to Show the Polarity of Public Opinion From Twitter Tweets IEEE Access, 11, 11811-11826 | NR | Obs | No | NR | 7. Social sciences | NR |
| Berdida, D. J. E. et al (2023). Human monkeypox infection knowledge in the Philippines: An online cross-sectional survey Heliyon, e13864 | Philippines | Obs | No | NR | 7. Social sciences | Adults |
| Berdida, D. J. E. et al (2023). Population-based survey of human monkeypox disease knowledge in the Philippines: An online cross-sectional study Journal of advanced nursing, 79(7), 2684-2694 | Philippines | Obs | No | NR | 7. Social sciences | Adults |
| Bert, F. et al (2024). Assessment of Italian Population Awareness on One-Health, Zoonoses and the Mpox Vaccine: A Nationwide Cross-Sectional Study Vaccines, 12(3), | Italy | Obs | No | NR | 7. Social sciences | NR |
| Bertran, M. et al (2023). Effectiveness of one dose of MVA-BN smallpox vaccine against mpox in England using the case-coverage method: an observational study The Lancet Infectious Diseases, 23(7), 828-835 | UK | Obs | No | NR | 3. Prophylaxis | Adults |
| Bhadra, A. et al (2023). Monkeypox, a global health concern: A study on awareness and adherence to preventive measures among medical students in a tertiary care hospital, Kolkata National Journal of Physiology, Pharmacy and Pharmacology, 13(2), 354-358 | India | Obs | No | NR | 7. Social sciences | Adults, Students |
| Bilgin, S. et al (2022). Evaluation of Knowledge and Attitudes of Anesthesiology Specialists and Residents Toward Patients Infected with the Monkeypox Disease: A National Survey Study Surgical Infections, 23(10), 880-886 | Turkey | Obs | No | NR | 7. Social sciences | Adults, Healthcare workers |
| Brighton, K. et al (2024). Targeted community wastewater surveillance for SARS-CoV-2 and Mpox virus during a festival mass-gathering event Science of the Total Environment, 906, 167443 | USA | Obs | No | NR | 4. Diagnostics | NR |
| Brihn, A. et al (2023). Countering Mpox Vaccination Disparities in Los Angeles County, California, May-December 2022 American journal of public health, 113(12), 1258-1262 | USA | Int | No | NR | 7. Social sciences | Adults, MSM |
| Brousseau, N. et al (2024). Single-dose Effectiveness of Mpox Vaccine in Quebec, Canada: Test-negative Design With and Without Adjustment for Self-reported Exposure Risk Clinical Infectious Diseases, 78(2), 461-469 | Canada | Obs | Yes | NR | 3. Prophylaxis | Adults |
| Cannon, C. A. et al (2024). Clinical Diagnosis is Highly Predictive of Laboratory-Confirmed Mpox in a Sexual Health Clinic Sexually transmitted diseases, | USA | Obs | Yes | NR | 4. Diagnostics | Adults |
| Carrico, S. et al (2023). Mpox Vaccination and the Role of Social Vulnerability in Durham County, North Carolina, USA Journal of racial and ethnic health disparities, | USA | Obs | No | NR | 7. Social sciences | Adults, Older adults, MSM |
| Caycho-Rodríguez, T. et al (2023). Intention to receive the monkeypox vaccine and its psychological and sociodemographic predictors: a cross-sectional survey in the general population of Peru Psicologia: Reflexao e Critica, 36(1), | Peru | Obs | No | NR | 7. Social sciences | Adults |
| Chan, J. et al (2023). Implementation and early outcomes of a telehealth visit model to deliver tecovirimat for mpox infection in New York City Journal of telemedicine and telecare, 1357633X231194796 | USA | Obs | No | NR | 5. Therapeutics | Adults, Transgender |
| Chelsky, Z. L. et al (2022). Validation Study of a Direct Real-Time PCR Protocol for Detection of Monkeypox Virus Journal of Molecular Diagnostics, 24(11), 1155-1159 | USA | Obs | No | NR | 4. Diagnostics | NR |
| Chen, Q. et al (2023). CRISPR-Cas12-based field-deployable system for rapid detection of synthetic DNA sequence of the monkeypox virus genome Journal of Medical Virology, 95(1), e28385 | NR | Int | No | NR | 4. Diagnostics | NR |
| Chen, Y. et al (2023). Ultrasensitive and Specific Clustered Regularly Interspaced Short Palindromic Repeats Empowered a Plasmonic Fiber Tip System for Amplification-Free Monkeypox Virus Detection and Genotyping ACS nano, 17(13), 12903-12914 | China | Int | No | NR | 4. Diagnostics | NR |
| Chen, Y. et al (2023). Knowledge of Human Mpox (Monkeypox) and Attitude towards Mpox Vaccination among Male Sex Workers in China: A Cross-Sectional Study Vaccines, 11(2), 285 | China | Obs | No | NR | 7. Social sciences | Adolescents, Adults, MSM, MSM |
| Cheung, D. H. et al (2024). Influences of mpox disease perceptions, sources and contents of information exposure on mpox vaccine uptake among gay, bisexual, and other men who have sex with men in Hong Kong, China Vaccine, | China | Obs | No | NR | 7. Social sciences | Adults, MSM |
| Choi, Y. et al (2023). Communities at Risk for Mpox and Stigmatizing Policies: A Randomized Survey, Republic of Korea, 2022 American journal of public health, 113(10), 1120-1127 | South Korea | Obs | No | NR | 7. Social sciences | Adults, MSM |
| Chow, E. P. F. et al (2023). Accessing first doses of mpox vaccine made available in Victoria, Australia The Lancet Regional Health - Western Pacific, 31, 100712 | Australia | Obs | No | NR | 7. Social sciences | Adults, MSM |
| Chow, E. P. F. et al (2023). Mpox knowledge, vaccination and intention to reduce sexual risk practices among men who have sex with men and transgender people in response to the 2022 mpox outbreak: A cross-sectional study in Victoria, Australia Sexual Health, 20(5), 390-402 | Australia | Obs | No | NR | 7. Social sciences | Adults, Transgender |
| Chu, H. et al (2023). Psychological Distance, Construal Level, and Parental Vaccine Hesitancy for COVID-19, HPV, and Monkey Pox Vaccines Science Communication, 45(3), 279-309 | USA | Obs | No | NR | 7. Social sciences | Adults |
| Clark, A. E. et al (2023). Validating Tools to Detect and Inactivate Monkeypox Virus in Human Milk Breastfeeding Medicine, 18(10), 785-789 | USA | Int | No | NR | 4. Diagnostics | NR |
| Copen, C. E. et al (2024). Modifications to Sexual Behaviors Associated With Mpox (Monkeypox) Virus Transmission Among Persons Presenting for mpox Vaccination, Washington, DC, August-October, 2022 Sexually Transmitted Diseases, 51(1), 54-60 | USA | Obs | No | NR | 7. Social sciences | Adults, MSM, PLHIV |
| Coppens, J. et al (2022). Alternative sampling specimens for the molecular detection of mpox (formerly monkeypox) virus Journal of clinical virology: the official publication of the Pan American Society for Clinical Virology, 159, 105372 | Belgium | Int | Yes | NR | 4. Diagnostics | NR |
| Crosato, V. et al (2024). Perception and Awareness about Monkeypox and Vaccination Acceptance in an At-Risk Population in Brescia, Italy: An Investigative Survey AIDS and behavior, | Italy | Obs | No | NR | 7. Social sciences | Adults, Sexual health clinic patients |
| Cui, X. et al (2023). Rapid detection of mpox virus using recombinase aided amplification assay Frontiers in Cellular and Infection Microbiology, 13, 1008783 | China | Int | Yes | I, II | 4. Diagnostics | NR |
| Dalton, A. F. et al (2023). Estimated Effectiveness of JYNNEOS Vaccine in Preventing Mpox: A Multijurisdictional Case-Control Study - United States, August 19, 2022-March 31, 2023 MMWR. Morbidity and mortality weekly report, 72(20), 553-558 | USA | Obs | Yes | NR | 3. Prophylaxis | Adults, MSM |
| Damhorst, G. L. et al (2024). Performance of the XpertTM Mpox PCR assay with oropharyngeal, anorectal, and cutaneous lesion swab specimens Journal of Clinical Virology, 171, 105659 | USA | Obs | No | NR | 4. Diagnostics | NR |
| Das, S. K. et al (2023). Socio-demographic determinants of the knowledge and attitude of Nepalese healthcare workers toward human monkeypox: a cross-sectional study Frontiers in public health, 11, 1161234 | Nepal | Obs | No | NR | 7. Social sciences | Adults, Healthcare workers |
| Das, S. K. et al (2024). Socio-demographic determinants of the knowledge of Monkeypox Virus among the general public: a cross-sectional study in a Tertiary Care Center in Nepal BMC Infectious Diseases, 24(1), 295 | Saudi Arabia | Obs | No | NR | 7. Social sciences | Adults |
| Del Duca, G. et al (2023). Risk Awareness as a Key Determinant of Early Vaccine Uptake in the Mpox Vaccination Campaign in an Italian Region: A Cross-Sectional Analysis Vaccines, 11(12), 1761 | Italy | Obs | No | NR | 7. Social sciences | Adults, those who received mpox vaccine |
| Deng, L. et al (2023). Short-term Adverse Events Following Immunization With Modified Vaccinia Ankara-Bavarian Nordic (MVA-BN) Vaccine for Mpox JAMA, 329(23), 2091-2094 | Australia | Obs | No | NR | 3. Prophylaxis | Adolescents, Adults |
| Denninger, J. et al (2023). The rapid implementation of a pharmacy student vaccination program with the Department of Health to increase the delivery of intradermal monkeypox vaccinations JACCP Journal of the American College of Clinical Pharmacy, 6(1), 49-52 | USA | Int | No | NR | 7. Social sciences | Adults, Students |
| Deputy, N. P. et al (2023). Vaccine Effectiveness of JYNNEOS against Mpox Disease in the United States New England Journal of Medicine, 388(26), 2434-2443 | USA | Obs | Yes | NR | 3. Prophylaxis | Adolescents, Adults, Older adults, PLHIV |
| Dong, C. et al (2023). Knowledge and vaccination intention of monkeypox in China's general population: A cross-sectional online survey Travel Medicine and Infectious Disease, 52, 102533 | China | Obs | No | NR | 7. Social sciences | Adults |
| Dsouza, V. S. et al (2023). A sentiment and content analysis of tweets on monkeypox stigma among the LGBTQ+ community: A cue to risk communication plan Dialogues Health, 2, 100095 | NR | Obs | No | NR | 7. Social sciences | Adults, LGBQTI |
| Duffy, J. et al (2022). Safety Monitoring of JYNNEOS Vaccine During the 2022 Mpox Outbreak — United States, May 22–October 21, 2022 Morbidity and Mortality Weekly Report, 71(49), 1555-1559 | USA | Obs | Yes | NR | 3. Prophylaxis | NR |
| Dukers-Muijrers, N. H. T. M. et al (2022). Mpox vaccination willingness, determinants, and communication needs in gay, bisexual, and other men who have sex with men, in the context of limited vaccine availability in the Netherlands (Dutch Mpox-survey) Frontiers in public health, 10, 1058807 | Netherlands | Obs | No | NR | 7. Social sciences | Adolescents, Adults, MSM |
| Elbaz, M. et al (2023). Diagnosis of Monkeypox infection: Validation of two diagnostic kits for viral detection using RT-PCR Journal of Virological Methods, 312, 114653 | Israel | Obs | Yes | West Africa clade | 4. Diagnostics | NR |
| Eliwa, E. H. I. et al (2023). Utilizing convolutional neural networks to classify monkeypox skin lesions Scientific Reports, 13(1), | Saudi Arabia | Int | No | NR | 4. Diagnostics | NR |
| Elsayed, A. A. et al (2023). The preparedness and knowledge of pharmacists and general practitioners in managing human monkeypox: a highly spreading infectious disease Journal of Pharmaceutical Policy and Practice, 16(1), 125 | Egypt | Obs | No | NR | 7. Social sciences | Adults, Healthcare workers |
| Erster, O. et al (2023). A Multi-Laboratory Evaluation of Commercial Monkeypox Virus Molecular Tests Microbiology Spectrum, 11(3), e00225-23 | USA | Obs | Yes | NR | 4. Diagnostics | NR |
| Farahat, R. A. et al (2022). Public perspectives of monkeypox in Twitter: A social media analysis using machine learning New Microbes and New Infections, 49-50, 101053 | NR | Obs | No | NR | 7. Social sciences | NR |
| Farrar, J. L. et al (2022). Demographic and Clinical Characteristics of Mpox in Persons Who Had Previously Received 1 Dose of JYNNEOS Vaccine and in Unvaccinated Persons - 29 U.S. Jurisdictions, May 22-September 3, 2022 MMWR. Morbidity and mortality weekly report, 71(5152), 1610-1615 | USA | Obs | Yes | NR | 3. Prophylaxis | Adults, Exposed contacts |
| Fattouh, R. et al (2024). Evaluation of 5 Polymerase Chain Reaction Assays for the Detection of Mpox Virus Journal of Infectious Diseases, 229(Supplement 2), S156-S162 | Canada | Obs | No | NR | 4. Diagnostics | NR |
| Feng, J. et al (2022). Development of a Loop-Mediated Isothermal Amplification Method for Rapid and Visual Detection of Monkeypox Virus Microbiology Spectrum, 10(5), | NR | Int | No | NR | 4. Diagnostics | NR |
| Ferré, V. M. et al (2022). Detection of Monkeypox Virus in Anorectal Swabs From Asymptomatic Men Who Have Sex With Men in a Sexually Transmitted Infection Screening Program in Paris, France Ann Intern Med, 175(10), 1491-1492 | France | Obs | No | NR | 4. Diagnostics | Adults, MSM |
| Filardo, T. D. et al (2023). Mpox vaccine acceptability among people experiencing homelessness in San Francisco - October-November 2022 Vaccine, 41(39), 5673-5677 | USA | Obs | No | NR | 7. Social sciences | Adults, Older adults, People experiencing homelessness (includes 18% MSM) |
| Fontan-Vela, M. et al (2024). Effectiveness of Modified Vaccinia Ankara-Bavaria Nordic Vaccination in a Population at High Risk of Mpox: A Spanish Cohort Study Clinical Infectious Diseases, 78(2), 476-483 | Spain | Obs | Yes | NR | 3. Prophylaxis | Adults, HIV risk group |
| Fresse, A. et al (2024). Adverse drug reaction profile of third-generation smallpox vaccines used in France during the 2022 monkeypox epidemic AIDS, 38(5), 768-771 | France | Obs | No | NR | 3. Prophylaxis | NR |
| Fu, L. et al (2023). Perception of and Vaccine Readiness towards Mpox among Men Who Have Sex with Men Living with HIV in China: A Cross-Sectional Study Vaccines, 11(3), 528 | China | Obs | No | NR | 7. Social sciences | Adults, MSM |
| Gagneux-Brunon, A. et al (2022). Attitudes towards monkeypox vaccination among healthcare workers in France and Belgium: an element of complacency? Journal of Hospital Infection, 130, 144-145 | Belgium, France | Obs | No | NR | 7. Social sciences | Adults, Healthcare workers |
| Galle, F. et al (2022). "Monkeypox: What Do You Know about That?" Italian Adults' Awareness of a New Epidemic Pathogens, 11(11), 1285 | Italy | Obs | No | NR | 7. Social sciences | Adults |
| Garba-Ouangole, S. et al (2023). Laboratory Diagnosis of Mpox, Central African Republic, 2016-2022 Emerging Infectious Diseases, 29(9), 1846-1849 | NR | Obs | Yes | I | 4. Diagnostics | Adolescents, Adults |
| Garrigues, J. M. et al (2023). Community spread of a human monkeypox virus variant with a tecovirimat resistance-associated mutation Antimicrobial Agents and Chemotherapy, 67(11), | USA | Obs | No | II | 5. Therapeutics | Adults |
| Garrigues, J. M. et al (2023). Identification of Tecovirimat Resistance-Associated Mutations in Human Monkeypox Virus - Los Angeles County Antimicrob Agents Chemother, 67(7), e0056823 | USA | Obs | No | II | 5. Therapeutics | NR |
| Garrigues, J. M. et al (2022). Identification of Human Monkeypox Virus Genome Deletions That Impact Diagnostic Assays Journal of Clinical Microbiology, 60(12), | USA | Obs | No | II | 4. Diagnostics | NR |
| Ghazy, R. M. et al (2022). Psychological Antecedents of Healthcare Workers towards Monkeypox Vaccination in Nigeria Vaccines, 10(12), 2151 | Nigeria | Obs | No | NR | 7. Social sciences | Adults, Healthcare workers |
| Ghazy, R. M. et al (2023). Monkeypox Vaccine Acceptance among Ghanaians: A Call for Action Vaccines, 11(2), 240 | Ghana | Obs | No | NR | 7. Social sciences | Adults |
| Gilbert, M. et al (2023). Uptake of Mpox vaccination among transgender people and gay, bisexual and other men who have sex with men among sexually-transmitted infection clinic clients in Vancouver, British Columbia Vaccine, 41(15), 2485-2494 | Canada | Obs | No | NR | 7. Social sciences | Adults, MSM, transgender |
| Gim, H. et al (2023). How do people tweet about gay and bisexual people surrounding the 2022 monkeypox outbreak? An NLP-based text analysis of tweets in the U.S Communication Research Reports, 40(5), 238-249 | USA | Obs | No | NR | 7. Social sciences | NR |
| Golden, M. R. et al (2024). Asymptomatic and Subclinical Mpox: An Association with Modified Vaccinia Ankara (MVA) Vaccine Sexually transmitted diseases, | USA | Obs | Yes | NR | 3. Prophylaxis, 4. Diagnostics | Adults, Sexual health clinic patients |
| Gong, L. et al (2023). Rapid, sensitive, and highly specific detection of monkeypox virus by CRISPR-based diagnostic platform Frontiers in public health, 11, 1137968 | China | Int | Yes | II | 4. Diagnostics | NR |
| Gonzales-Zamora, J. A. et al (2023). Level of Knowledge Regarding Mpox among Peruvian Physicians during the 2022 Outbreak: A Cross-Sectional Study Vaccines, 11(1), | Peru | Obs | No | NR | 7. Social sciences | Adults, Healthcare workers |
| Grov, C. et al (2024). Sexual and Gender Minorities' Vaccine Uptake and Behavioral Change in Response to the Mpox Outbreak in the United States: August 2022 Through November 2022 Clin Infect Dis, | USA | Obs | No | NR | 7. Social sciences | Adults, MSM |
| Groves, J. A. et al (2023). Nucleic acid testing for monkeypox in United States blood donor specimens Transfusion, 63(10), 1797-1802 | USA | Int | No | NR | 4. Diagnostics | Adults, PLHIV |
| Gu, Y. et al (2024). Knowledge, Attitude, and Practice Towards Mpox and Associated Factors Among HIV-Infected Individuals - Beijing Municipality, China, 2023 China CDC Wkly, 6(7), 109-117 | China | Obs | No | NR | 7. Social sciences | Adults, PLHIV |
| Halboup, A. M. et al (2023). Perceptions and Knowledge of Public Towards Emerging Human Monkeypox in Yemen: A Cross-Sectional Study Journal of Multidisciplinary Healthcare, 16, 3689-3701 | Yemen | Obs | No | NR | 7. Social sciences | Adults |
| Hasan, M. et al (2023). Human monkeypox and preparedness of Bangladesh: A knowledge and attitude assessment study among medical doctors Journal of Infection and Public Health, 16(1), 90-95 | Bangladesh | Obs | No | NR | 7. Social sciences | Adults, Healthcare workers |
| Hassan, R. et al (2024). Mpox Knowledge, Attitudes, and Practices Among Persons Presenting for JYNNEOS Vaccination - District of Columbia, August to October 2022 Sexually Transmitted Diseases, 51(1), 47-53 | Colombia | Obs | No | NR | 7. Social sciences | Adults |
| Hayes, R. et al (2024). Cross-sectional survey of sexual health professionals' experiences and perceptions of the 2022 mpox outbreak in the UK BMJ Open, 14(1), e080250 | UK | Obs | No | NR | 7. Social sciences | Adults, Healthcare workers |
| Hazra, A. et al (2022). Human Monkeypox Virus Infection in the Immediate Period after Receiving Modified Vaccinia Ankara Vaccine JAMA, 328(20), 2064-2067 | USA | Obs | Yes | NR | 3. Prophylaxis | Adults |
| Hong, C. et al (2023). Mpox on Reddit: a Thematic Analysis of Online Posts on Mpox on a Social Media Platform among Key Populations Journal of urban health: bulletin of the New York Academy of Medicine, 100(6), 1264-1273 | NR | Obs | No | NR | 7. Social sciences | NR |
| Hori, D. et al (2023). Sexual orientation was associated with intention to be vaccinated with a smallpox vaccine against mpox: A cross-sectional preliminary survey in Japan Vaccine, 41(27), 3954-3959 | Japan | Obs | No | NR | 7. Social sciences | Adults |
| Howard, B. J. et al (2023). Mpox Vaccine Interest Survey Prioritization and Data Flow: Maricopa County, Arizona, July-August 2022 American journal of public health, 113(5), 504-508 | USA | Obs | No | NR | 7. Social sciences | Adults |
| Huang, M. F. et al (2024). Factors Related to Mpox-Vaccine Uptake among Men Who Have Sex with Men in Taiwan: Roles of Information Sources and Emotional Problems Vaccines, 12(3), 332 | Taiwan | Obs | No | NR | 7. Social sciences | Adults, MSM |
| Huang, P. et al (2023). A visual assay panel for the identification of monkeypox virus DNA belonging to the clades I and II Virol Sin, 38(4), 635-638 | China | Obs | No | I, II | 4. Diagnostics | NR |
| Huang, X. et al (2024). Willingness to accept monkeypox vaccine and its correlates among men who have sex with men in Southern China: a web-based online cross-sectional study Frontiers in public health, 12, 1289918 | China | Obs | No | NR | 7. Social sciences | Adults, MSM |
| Huang, X. et al (2023). Loop-mediated isothermal amplification combined with lateral flow biosensor for rapid and sensitive detection of monkeypox virus Frontiers in public health, 11, 1132896 | China | Int | No | I, II | 4. Diagnostics | NR |
| Huang, Y. et al (2024). Residual Immunity from Smallpox Vaccination and Possible Protection from Mpox, China Emerging Infectious Diseases, 30(2), 321-324 | China | Obs | No | NR | 3. Prophylaxis | Infants (<12 months old), Young Children (1-5 years), Children (5-13 years), Adolescents, Adults, Older adults |
| Hubert, M. et al (2023). Complement-dependent mpox-virus-neutralizing antibodies in infected and vaccinated individuals Cell Host and Microbe, 31(6), 937-948.e4 | France | Obs | Yes | NR | 3. Prophylaxis, 4. Diagnostics | NR |
| Huo, S. et al (2022). Development of two multiplex real-time PCR assays for simultaneous detection and differentiation of monkeypox virus IIa, IIb, and I clades and the B.1 lineage Biosaf Health, 4(6), 392-398 | NR | Obs | No | I, IIa, IIb | 4. Diagnostics | NR |
| Hurley, S. et al (2024). Mpox detection in clinical specimens by three commercial real-time PCR assays demonstrates comparable results Pathology, | Australia | Obs | No | NR | 4. Diagnostics | NR |
| Ibrahim, A. M. et al (2023). Knowledge and attitudes towards mpox and effect of intervention among College of Applied Medical Sciences students Libyan Journal of Medicine, 18(1), 2222448 | Saudi Arabia | Int | No | NR | 7. Social sciences | Adults, Students |
| Ilchmann, H. et al (2023). One-and Two-Dose Vaccinations with Modified Vaccinia Ankara-Bavarian Nordic Induce Durable B-Cell Memory Responses Comparable to Replicating Smallpox Vaccines Journal of Infectious Diseases, 227(10), 1203-1213 | NR | Int | Yes | NR | 3. Prophylaxis | Adults |
| Irshad, U. et al (2023). Implementation of an Mpox Vaccination Program at a Large Sexual Health Clinic in the Bronx-Lessons in Vaccine Equity Open Forum Infectious Diseases, 10(11), ofad544 | USA | Int | No | NR | 7. Social sciences | Adults, Older adults, PLHIV, People with other specific comorbidities |
| Islam, M. A. et al (2023). Knowledge, attitude, and practice of university students towards monkeypox in Bangladesh PLoS ONE, 18(10 October), e0287407 | Bangladesh | Obs | No | NR | 7. Social sciences | Adults, Students |
| Islam, M. R. et al (2023). Assessment of vaccine perception and vaccination intention of Mpox infection among the adult males in Bangladesh: A cross-sectional study findings PLoS ONE, 18(6 June), e0286322 | Bangladesh | Obs | No | NR | 7. Social sciences | Adults |
| Israeli, O. et al (2022). Rapid Amplicon Nanopore Sequencing (RANS) for the Differential Diagnosis of Monkeypox Virus and Other Vesicle-Forming Pathogens Viruses, 14(8), 1817 | Israel | Int | No | I, II | 4. Diagnostics | NR |
| Jamaleddine, Y. et al (2023). Knowledge and attitude towards monkeypox among the Lebanese population and their attitude towards vaccination Journal of Preventive Medicine and Hygiene, 64(1), E13-E26 | Lebanon | Obs | No | NR | 7. Social sciences | Adults |
| Jamil, H. et al (2023). Socio-demographic determinants of Monkeypox virus preventive behavior: A cross-sectional study in Pakistan PLoS ONE, 18(8 August), e0279952 | Pakistan | Obs | No | NR | 7. Social sciences | Adults |
| Jiang, T. et al (2023). Creating an ultra-sensitive detection platform for monkeypox virus DNA based on CRISPR technology Journal of Medical Virology, 95(7), e28905 | China | Int | Yes | NR | 4. Diagnostics | NR |
| Jiang, W. et al (2024). A cross-sectional investigation of factors influencing mpox vaccine hesitancy for students in Southwest China Human Vaccines and Immunotherapeutics, 20(1), 2309704 | China | Obs | No | NR | 7. Social sciences | Adolescents, Adults, Students |
| Jiao, K. et al (2023). Mpox risk perception and associated factors among Chinese young men who have sex with men: Results from a large cross-sectional survey Journal of Medical Virology, 95(8), e29057 | China | Obs | No | NR | 7. Social sciences | Adults, MSM |
| Jin, B. et al (2024). Point-of-care detection of Monkeypox virus clades using high-performance upconversion nanoparticle-based lateral flow assay Mikrochimica acta, 191(4), 177 | China | Obs | No | II | 4. Diagnostics | NR |
| Jongen, V. W. et al (2024). What determines mpox vaccination uptake? Assessing the effect of intent-to-vaccinate versus other determinants among men who have sex with men Vaccine, 42(2), 186-193 | Netherlands | Obs | No | NR | 7. Social sciences | Adolescents, Adults, MSM |
| Kahn, P. A. et al (2023). Availability and Accessibility of Live Nonreplicating Smallpox/Mpox Vaccine JAMA Network Open, 6(4), E237873 | USA | Obs | No | NR | 7. Social sciences | NR |
| Karapinar, A. et al (2023). Awareness and acceptability of monkeypox vaccine in men who have sex with men Turkish Journal of Medical Sciences, 53(5), 1136-1143 | Turkey | Obs | No | NR | 7. Social sciences | Adults, MSM |
| Karmarkar, E. N. et al (2024). Association of Tecovirimat Therapy With Mpox Symptom Improvement: A Cross-sectional Study-King County, Washington, May-October 2022 Open Forum Infectious Diseases, 11(3), ofae029 | USA | Obs | No | NR | 5. Therapeutics | Adults |
| Kato, M. et al (2023). Trends in Media Coverage During the Monkeypox Outbreak: Content Analysis J Med Internet Res, 25, e45787 | Japan | Obs | No | NR | 7. Social sciences | Adults 18-65, general population |
| Kaur, A. et al (2022). Identifying monkeypox: do dental professionals have adequate knowledge and awareness? Roczniki Panstwowego Zakladu Higieny, 73(3), 365-371 | India | Obs | No | NR | 7. Social sciences | Adults, Healthcare workers |
| Kinganda-Lusamaki, E. et al (2023). Use of Mpox Multiplex Serology in the Identification of Cases and Outbreak Investigations in the Democratic Republic of the Congo (DRC) Pathogens, 12(7), 916 | Democratic Republic of the Congo | Obs | Yes | NR | 4. Diagnostics | Children (5-13 years), Adolescents, Adults |
| Kingsley, C. O. et al (2023). KNOWLEDGE OF MPOX AMONG PRIMARY HEALTH CARE (PHC) WORKERS IN JOS NORTH LOCAL GOVERNMENT AREA, PLATEAU STATE. NIGERIA West Afr J Med, 40(11 Suppl 1), S17 | Nigeria | Obs | No | NR | 7. Social sciences | Adults, Healthcare workers |
| Kota, K. K. et al (2023). Progress Toward Equitable Mpox Vaccination Coverage: A Shortfall Analysis - United States, May 2022-April 2023 MMWR. Morbidity and mortality weekly report, 72(23), 627-632 | USA | Obs | No | NR | 7. Social sciences | Adolescents, Adults, MSM, PLHIV |
| Kota, K. K. et al (2023). Racial and Ethnic Disparities in Mpox Cases and Vaccination Among Adult Males - United States, May-December 2022 MMWR. Morbidity and mortality weekly report, 72(15), 398-403 | USA | Obs | No | NR | 7. Social sciences | Adults |
| Kottkamp, A. C. et al (2023). Antibody Titers against Mpox Virus after Vaccination New England Journal of Medicine, 389(24), 2299-2301 | USA | Obs | No | NR | 3. Prophylaxis | NR |
| Kriss, J. L. et al (2022). Receipt of First and Second Doses of JYNNEOS Vaccine for Prevention of Monkeypox - United States, May 22-October 10, 2022 MMWR. Morbidity and mortality weekly report, 71(43), 1374-1378 | USA | Obs | No | NR | 7. Social sciences | Infants (<12 months old), Young children (1 -5 years old), Children (5-13 years), Adolescents (13-17 years), Older adults (>65 years) |
| Kumar, N. et al (2023). Monkeypox Cross-Sectional Survey of Knowledge, Attitudes, Practices, and Willingness to Vaccinate among University Students in Pakistan Vaccines, 11(1), 97 | Pakistan | Obs | No | NR | 7. Social sciences | Adults, Students |
| Ladhani, S. N. et al (2023). Early evaluation of the safety, reactogenicity, and immune response after a single dose of modified vaccinia Ankara-Bavaria Nordic vaccine against mpox in children: a national outbreak response The Lancet Infectious Diseases, 23(9), 1042-1050 | UK | Obs | No | IIb | 3. Prophylaxis | Young children (1 -5 years old), Children (5-13 years), Adolescents (13-17 years) |
| Lakshmi, M. et al (2023). Classification of Monkeypox Images Using LIME-Enabled Investigation of Deep Convolutional Neural Network Diagnostics, 13(9), 1639 | India | Obs | No | NR | 4. Diagnostics | NR |
| Lash, M. K. et al (2023). Racial and Socioeconomic Equity of Tecovirimat Treatment during the 2022 Mpox Emergency, New York, New York, USA Emerging Infectious Diseases, 29(11), 2353-2357 | USA | Obs | No | NR | 7. Social sciences | Adolescents, Adults, Older adults |
| Lazarus, J. E. et al (2024). Separating the rash from the chaff: novel clinical decision support deployed during the mpox outbreak Infect Control Hosp Epidemiol, 1-3 | USA | Int | Yes | NR | 4. Diagnostics | NR |
| Le Forestier, J. M. et al (2024). Identity Concealment May Discourage Health-Seeking Behaviors: Evidence From Sexual-Minority Men During the 2022 Global Mpox Outbreak Psychological science, 35(2), 126-136 | Australia, Canada, UK, USA | Obs | No | NR | 7. Social sciences | Adults, MSM |
| Lee, J. et al (2023). JYNNEOS vaccine safety monitoring in the Republic of Korea, 2022: a cross-sectional study Osong Public Health and Research Perspectives, 14(5), 433-438 | North Korea, South Korea | Obs | No | NR | 3. Prophylaxis | Adults |
| Li, E. et al (2023). Duration of humoral immunity from smallpox vaccination and its cross-reaction with Mpox virus Signal Transduction and Targeted Therapy, 8(1), 350 | China | Obs | No | NR | 3. Prophylaxis | Adults |
| Li, L. et al (2024). Survey on monkeypox knowledge awareness, risk perception and vaccination intention in men who have sex with men in five cities in northeast China Chinese Journal of Epidemiology, 45(1), 128-133 | China | Obs | No | NR | 7. Social sciences | Adults, MSM |
| Li, Y. et al (2023). Monkeypox awareness and low vaccination hesitancy among men who have sex with men in China Journal of Medical Virology, 95(2), e28567 | China | Obs | No | NR | 7. Social sciences | Adults, MSM |
| Li, Z. et al (2023). Extraction-free LAMP assays for generic detection of Old World Orthopoxviruses and specific detection of Mpox virus Sci Rep, 13(1), 21093 | USA | Int | No | I, II, IIb | 4. Diagnostics | NR |
| Lim, S. Y. et al (2024). Adverse Reactions After Intradermal Vaccination With JYNNEOS for Mpox in Korea Journal of Korean medical science, 39(8), e100 | South Korea | Obs | No | NR | 3. Prophylaxis | Adults, at risk group |
| Lima, A. et al (2024). Validation of a new extraction-free real-time PCR test to detect MPOX virus Diagnostic Microbiology and Infectious Disease, 109(2), 116265 | USA | Obs | No | NR | 4. Diagnostics | NR |
| Lin, G. et al (2022). Monkeypox awareness, knowledge, and attitude among undergraduate preclinical and clinical students at a Malaysian dental school: An emerging outbreak during the COVID-19 era Asian Pacific Journal of Tropical Medicine, 15(10), 461-467 | Malaysia | Obs | No | NR | 7. Social sciences | Adults, Students |
| Liotti, F. M. et al (2023). Performance of a Novel Real-Time PCR-Based Assay for Rapid Monkeypox Virus Detection in Human Samples Microorganisms, 11(10), | Italy | Obs | No | II | 4. Diagnostics | Adults, PLHIV |
| Lounis, M. et al (2023). Knowledge and awareness of Algerian healthcare workers about human monkeypox and their attitude toward its vaccination: An online cross-sectional survey Vacunas, 24(2), 122-127 | Algeria | Obs | No | NR | 7. Social sciences | Adults, Healthcare workers |
| Lounis, M. et al (2024). Assessment of Monkeypox (MPOX) Knowledge and Vaccination Intention among Health and Life Sciences Students in Algeria: A Cross-Sectional Study Infect Dis Rep, 16(2), 170-180 | Algeria | Obs | No | NR | 7. Social sciences | Adults, Students |
| Low, S. J. et al (2023). Rapid detection of monkeypox virus using a CRISPR-Cas12a mediated assay: a laboratory validation and evaluation study The Lancet Microbe, 4(10), e800-e810 | Italy | Obs | Yes | IIb | 4. Diagnostics | NR |
| Luo, S. et al (2024). Behavioral Intention of Receiving Monkeypox Vaccination and Undergoing Monkeypox Testing and the Associated Factors Among Young Men Who Have Sex With Men in China: Large Cross-Sectional Study JMIR public health and surveillance, 10, e47165 | China | Obs | No | NR | 7. Social sciences | Adults |
| Macgibbon, J. et al (2023). Mpox (monkeypox) knowledge, concern, willingness to change behaviour, and seek vaccination: Results of a national cross-sectional survey Sexual Health, 20(5), 403-410 | Australia | Obs | No | NR | 7. Social sciences | Adults, MSM, Non-binary |
| Mahameed, H. et al (2023). Previous Vaccination History and Psychological Factors as Significant Predictors of Willingness to Receive Mpox Vaccination and a Favorable Attitude towards Compulsory Vaccination Vaccines, 11(5), 897 | Jordan | Obs | No | NR | 7. Social sciences | Adults, Healthcare workers |
| Malaeb, D. et al (2023). Knowledge, Attitude and Conspiracy Beliefs of Healthcare Workers in Lebanon towards Monkeypox Tropical Medicine and Infectious Disease, 8(2), 81 | Lebanon | Obs | No | NR | 7. Social sciences | Adults, Healthcare workers |
| Mancon, A. et al (2024). Evaluation of Analytical Performance of the STANDARD(TM) M10 MPX/OPX Assay for the Simultaneous DNA Detection and Clade Attribution of Monkeypox virus Emerg Microbes Infect, 2337666 | Italy | Obs | Yes | NR | 4. Diagnostics | NR |
| Mao, L. et al (2022). Development and Characterization of Recombinase-Based Isothermal Amplification Assays (RPA/RAA) for the Rapid Detection of Monkeypox Virus Viruses, 14(10), 2112 | Central African Republic | Obs | No | West Africa clade | 4. Diagnostics | NR |
| Martins-Filho, P. R. et al (2023). Differences in cycle threshold values in RT-PCR tests between children and adults with monkeypox: Results from a community-based cross-sectional study Travel Med Infect Dis, 52, 102560 | Brazil | Obs | Yes | NR | 4. Diagnostics | Young Children (1-5 years), Children (5-13 years), Adolescents, Adults |
| Masarwa, R. et al (2024). Knowledge of the Mpox virus and conspiracy beliefs and their association with self-confidence in managing the virus among Israeli orthopedic surgeons Pathogens and Global Health, 118(1), 33-39 | Israel | Obs | No | NR | 7. Social sciences | Adults, Healthcare workers |
| Masood, S. et al (2023). Knowledge of Human Monkeypox Infection among Final Year Medical, Pharmacy, and Nursing Students: A Multicenter, Cross-Sectional Analysis from Pakistan Healthcare (Basel), 11(20), | Pakistan | Obs | No | NR | 7. Social sciences | Adults, Students |
| May, T. et al (2023). Mpox knowledge, behaviours and barriers to public health measures among gay, bisexual and other men who have sex with men in the UK: a qualitative study to inform public health guidance and messaging BMC public health, 23(1), 2265 | UK | Obs | No | NR | 7. Social sciences | Adults |
| Mazzotta, V. et al (2023). Effect of tecovirimat on healing time and viral clearance by emulation of a target trial in patients hospitalized for mpox Journal of Medical Virology, 95(6), e28868 | Italy | Obs | Yes | NR | 5. Therapeutics | Adults, MSM, In-hospital patients |
| Mazzotta, V. et al (2024). Immunogenicity and reactogenicity of modified vaccinia Ankara pre-exposure vaccination against mpox according to previous smallpox vaccine exposure and HIV infection: prospective cohort study eClinicalMedicine, 68, 102420 | Italy | Obs | Yes | NR | 3. Prophylaxis | Adults, MSM |
| McKinley, C. J. et al (2024). Expressing Uncertainty and Risk About the Mpox Outbreak: A Textual Analysis of Twitter Messaging Communication Studies, | NR | Obs | No | NR | 7. Social sciences | Adults, general population |
| McLean, J. et al (2023). Tecovirimat Treatment of People with HIV during the 2022 Mpox Outbreak; A Retrospective Cohort Study Annals of Internal Medicine, 176(5), 642-648 | USA | Obs | Yes | NR | 5. Therapeutics | NR |
| Meena, G. et al (2024). Monkeypox recognition and prediction from visuals using deep transfer learning-based neural networks Multimedia Tools and Applications, | NR | Obs | No | NR | 4. Diagnostics | NR |
| Meo, S. A. et al (2022). Public Perceptions of the Emerging Human Monkeypox Disease and Vaccination in Riyadh, Saudi Arabia: A Cross-Sectional Study Vaccines, 10(9), 1534 | Saudi Arabia | Obs | No | NR | 7. Social sciences | Adults |
| Merad, Y. et al (2022). Outcomes of post-exposure vaccination by modified vaccinia Ankara to prevent mpox (formerly monkeypox): a retrospective observational study in Lyon, France, June to August 2022 Eurosurveillance, 27(50), 2200882 | France | Obs | No | NR | 3. Prophylaxis | Adults, PLHIV |
| Michel, J. et al (2022). Evaluation of 11 commercially available PCR kits for the detection of monkeypox virus DNA, Berlin, July to September 2022 Eurosurveillance, 27(45), | Germany | Obs | No | I, II | 4. Diagnostics | NR |
| Millman, A. J. et al (2022). A Health Equity Approach for Implementation of JYNNEOS Vaccination at Large, Community-Based LGBTQIA+ Events - Georgia, August 27-September 5, 2022 MMWR. Morbidity and mortality weekly report, 71(43), 1382-1883 | USA | Obs | No | NR | 7. Social sciences | Neonates (<28 days), Infants (<12 months old), Young Children (1-5 years), Children (5-13 years), Adolescents, Adults, Older adults, MSM, LGBTQIA+ communities of color |
| Mills, M. G. et al (2022). Evaluation and clinical validation of monkeypox (mpox) virus real-time PCR assays Journal of clinical virology: the official publication of the Pan American Society for Clinical Virology, 159, 105373 | USA | Obs | Yes | NR | 4. Diagnostics | NR |
| Miraglia Del Giudice, G. et al (2023). Knowledge and attitudes of health care workers about monkeypox virus infection in Southern Italy Frontiers in public health, 11, 1091267 | Italy | Obs | No | NR | 7. Social sciences | Adults, Healthcare workers |
| Montalti, M. et al (2023). Safety of Monkeypox Vaccine Using Active Surveillance, Two-Center Observational Study in Italy Vaccines, 11(7), 1163 | Italy | Int | No | NR | 3. Prophylaxis | Adults, MSM, People with other specific comorbidities |
| Morales, L. M. et al (2023). Post-exposure vaccine effectiveness and contact management in the mpox outbreak, Madrid, Spain, May to August 2022 Eurosurveillance, 28(24), | Spain | Int | Yes | NR | 3. Prophylaxis | Adults, MSM |
| Moretti, M. et al (2023). Diagnostic approach to monkeypox outbreak, a case-control study International Journal of STD and AIDS, 34(5), 338-345 | Belgium | Obs | Yes | NR | 4. Diagnostics | Adults, MSM, PLHIV, having multiple sexual partners |
| Morino, E. et al (2024). Mpox Neutralizing Antibody Response to LC16m8 Vaccine in Healthy Adults NEJM Evid, 3(3), EVIDoa2300290 | Japan | Int | No | I, IIa | 3. Prophylaxis | Adults, Healthcare workers |
| Moschese, D. et al (2023). Neutralizing Antibody Titers Induced by JYNNEOS Vaccine in Unrecognized Previous Mpox Virus-Exposed Individuals Clinical Infectious Diseases, 77(10), 1484-1485 | Italy | Int | No | NR | 3. Prophylaxis | Adults |
| Moschetta, N. et al (2023). Mpox neutralising antibodies at 6 months from mpox infection or MVA-BN vaccination: a comparative analysis The Lancet Infectious Diseases, 23(11), e455-e456 | Italy | Obs | No | NR | 3. Prophylaxis | Adults |
| Muller, M. P. et al (2024). Prospective monitoring of adverse events following vaccination with Modified vaccinia Ankara - Bavarian Nordic (MVA-BN) administered to a Canadian population at risk of Mpox: A Canadian Immunization Research Network study Vaccine, 42(3), 535-540 | Canada | Obs | No | NR | 3. Prophylaxis | Adults |
| Navarrete-Mejía, P. J. et al (2022). Knowledge about monkeypox among health professionals, Lima-Peru Revista del Cuerpo Medico Hospital Nacional Almanzor Aguinaga Asenjo, 15(2), 252-255 | Peru | Obs | No | NR | 7. Social sciences | Adults |
| Nazmunnahar et al (2023). Assessment of risk perception and subjective norms related to Mpox (monkeypox) among adult males in Bangladesh: A cross-sectional study Health Science Reports, 6(6), e1352 | Bangladesh | Obs | No | NR | 7. Social sciences | Adults |
| Ng, Q. X. et al (2022). Public sentiment on the global outbreak of monkeypox: an unsupervised machine learning analysis of 352,182 twitter posts Public Health, 213, 1-4 | NR | Obs | No | NR | 7. Social sciences | NR |
| Nimbi, F. M. et al (2023). Who Is Afraid of Monkeypox? Analysis of Psychosocial Factors Associated with the First Reactions of Fear of Monkeypox in the Italian Population Behavioral Sciences, 13(3), | Italy | Obs | No | NR | 7. Social sciences | Adults, Older adults |
| Nimbi, F. M. et al (2023). Monkeypox: New epidemic or fake news? Study of psychological and social factors associated with fake news attitudes of monkeypox in Italy Frontiers in Psychology, 14, | Italy | Obs | No | NR | 7. Social sciences | Adults, Older adults |
| Nimbi, F. M. et al (2023). Monkeypox spread among men who have sex with men: how do people explain this relationship? A quali-quantitative study of beliefs among heterosexual and non-heterosexual Italian individuals Psychology and Sexuality, | Italy | Obs | No | NR | 7. Social sciences | Adults, Older adults, MSM |
| Nka, A. D. et al (2024). Current knowledge of human Mpox viral infection among healthcare workers in Cameroon calls for capacity-strengthening for pandemic preparedness Frontiers in Public Health, 12, | Cameroon | Obs | No | NR | 7. Social sciences | Adults, Healthcare workers |
| Norz, D. et al (2022). Rapid Adaptation of Established High-Throughput Molecular Testing Infrastructure for Monkeypox Virus Detection Emerging Infectious Diseases, 28(9), 1765-1769 | Germany | Int | No | NR | 4. Diagnostics | NR |
| Nusynowitz, J. et al (2023). Medical Students' Knowledge and Perceptions of Mpox in a High Incidence Region: Implications for Clinical Preparedness PRiMER, 7, 37 | USA | Obs | No | NR | 7. Social sciences | Adults, Students |
| O’Neal, J. et al (2023). Reversing Inequity in Mpox Vaccine Distribution, Fulton County, Georgia, June–September 2022 American Journal of Public Health, 113(12), 1263-1266 | Georgia | Int | No | NR | 7. Social sciences | Adults, MSM |
| Ogaz, D. et al (2024). Mpox Diagnosis, Behavioral Risk Modification, and Vaccination Uptake among Gay, Bisexual, and Other Men Who Have Sex with Men, United Kingdom, 2022 Emerg Infect Dis, 30(5), | UK | Obs | No | NR | 7. Social sciences | Adults, MSM, bisexuals |
| Ogunbajo, A. et al (2023). Demographics and Health Beliefs of Black Gay, Bisexual, and Other Sexual Minority Men Receiving a Mpox Vaccination in the United States Journal of Urban Health, 100(1), 204-211 | USA | Obs | No | NR | 7. Social sciences | Adults, MSM, Black sexual minority men |
| O'Laughlin, K. et al (2022). Clinical Use of Tecovirimat (Tpoxx) for Treatment of Monkeypox Under an Investigational New Drug Protocol - United States, May-August 2022 MMWR Morb Mortal Wkly Rep, 71(37), 1190-1195 | USA | Obs | No | II | 5. Therapeutics | Neonates (<28 days), Infants (<12 months old), Young Children (1-5 years), Children (5-13 years), Adolescents, Adults, Older adults, PLHIV |
| Ortiz-Martínez, Y. et al (2022). Monkeypox goes viral: measuring the misinformation outbreak on Twitter J Infect Dev Ctries, 16(7), 1218-1220 | NR | Obs | No | NR | 7. Social sciences | NR |
| Ouafi, M. et al (2023). Oropharyngeal samples versus lesion specimens at diagnosis in patients infected with monkeypox virus in Northern France Journal of Medical Virology, 95(1), e28276 | France | Obs | No | NR | 4. Diagnostics | Adults |
| Owens, C. et al (2023). An Exploratory Study of the Mpox Media Consumption, Attitudes, and Preferences of Sexual and Gender Minority People Assigned Male at Birth in the United States LGBT health, 10(5), 401-407 | USA | Obs | No | NR | 7. Social sciences | Adults, PLHIV, sexual and gender minorities |
| Owens, C. et al (2023). Rural-urban differences in monkeypox behaviors and attitudes among men who have sex with men in the United States The Journal of rural health: official journal of the American Rural Health Association and the National Rural Health Care Association, 39(2), 508-515 | USA | Obs | No | NR | 7. Social sciences | Adults, MSM |
| Oztel, I. et al (2023). Deep Learning-Based Skin Diseases Classification using Smartphones Advanced Intelligent Systems, 5(12), | Turkey | Obs | Yes | NR | 4. Diagnostics | NR |
| Paniz-Mondolfi, A. et al (2023). Evaluation and validation of an RT-PCR assay for specific detection of monkeypox virus (MPXV) J Med Virol, 95(1), e28247 | USA | Obs | No | NR | 4. Diagnostics | Adults |
| Papadakis, G. et al (2023). Evaluation of 16 molecular assays for the detection of orthopox and mpox viruses Journal of Clinical Virology, 161, 105424 | Australia | Int | No | NR | 4. Diagnostics | NR |
| Paparini, S. et al (2023). Public understanding and awareness of and response to monkeypox virus outbreak: A cross-sectional survey of the most affected communities in the United Kingdom during the 2022 public health emergency HIV Med, 24(5), 544-557 | UK | Obs | No | NR | 7. Social sciences | Adults, at risk group |
| Park, S. Y. et al (2024). Detection of Mpox Virus Using Microbial Cell-Free DNA: The Potential of Pathogen-Agnostic Sequencing for Rapid Identification of Emerging Pathogens Journal of Infectious Diseases, 229(Supplement 2), S144-S155 | NR | Obs | No | NR | 4. Diagnostics | Adults, MSM, PLHIV |
| Passini, F. et al (2023). Mpox Outbreak 2022: A Comparative Analysis of the Characteristics of Individuals Receiving MVA-BN Vaccination and People Diagnosed with Mpox Infection in Milan, Italy Pathogens, 12(9), 1079 | Italy | Obs | Yes | NR | 7. Social sciences | Adults, MSM |
| Paudel, K. et al (2023). Uncovering the knowledge gaps: A survey on human monkeypox infection among men who have sex with men in Nepal Health Prospect, 22(1), 1-6 | Nepal | Obs | No | NR | 7. Social sciences | Adults, MSM |
| Payne, A. B. et al (2022). Reduced Risk for Mpox After Receipt of 1 or 2 Doses of JYNNEOS Vaccine Compared with Risk Among Unvaccinated Persons - 43 U.S. Jurisdictions, July 31-October 1, 2022 MMWR. Morbidity and mortality weekly report, 71(49), 1560-1564 | USA | Int | No | NR | 3. Prophylaxis | Adults, MSM, Gay and bisexuals |
| Payne, A. B. et al (2022). Incidence of Monkeypox Among Unvaccinated Persons Compared with Persons Receiving ≥1 JYNNEOS Vaccine Dose - 32 U.S. Jurisdictions, July 31-September 3, 2022 MMWR Morb Mortal Wkly Rep, 71(40), 1278-1282 | USA | Obs | Yes | NR | 3. Prophylaxis | Adults |
| Peng, X. et al (2023). Perceptions and worries about monkeypox, and attitudes towards monkeypox vaccination among medical workers in China: A cross-sectional survey Journal of Infection and Public Health, 16(3), 346-353 | China | Obs | No | NR | 7. Social sciences | Adults, Healthcare workers |
| Peptan, C. et al (2022). Study on the Vaccination of the Population of Romania against Monkeypox in Terms of Medical Security Vaccines, 10(11), 1834 | Romania | Obs | No | NR | 7. Social sciences | Adults |
| Phillips, G. et al (2023). Changes in Sexual Behaviors Due to Mpox: a Cross-Sectional Study of Sexual and Gender Minority Individuals in Illinois Prevention science: the official journal of the Society for Prevention Research, | USA | Obs | No | NR | 7. Social sciences | Adolescents, Adults |
| Pomari, E. et al (2023). Evaluation of a ddPCR Commercial Assay for the Absolute Quantification of the Monkeypox Virus West Africa in Clinical Samples Diagnostics, 13(7), 1349 | NR | Obs | Yes | I, IIa, IIb | 4. Diagnostics | NR |
| Pond, M. J. et al (2023). Mpox infection investigation using multiplexed syndromic diagnostics: Evaluation of an AusDiagnostics multiplexed tandem PCR (MT-PCR) syndromic panel Journal of Clinical Virology Plus, 3(2), 100142 | UK | Obs | No | NR | 4. Diagnostics | NR, People with other specific comorbidities |
| Porzucek, A. J. et al (2023). Development of an Accessible and Scalable Quantitative Polymerase Chain Reaction Assay for Monkeypox Virus Detection Journal of Infectious Diseases, 227(9), 1084-1087 | USA | Obs | No | NR | 4. Diagnostics | NR |
| Priyamvada, L. et al (2022). Serological responses to the MVA-based JYNNEOS monkeypox vaccine in a cohort of participants from the Democratic Republic of Congo Vaccine, 40(50), 7321-7327 | Democratic Republic of the Congo | Int | No | NR | 3. Prophylaxis | Adults, Healthcare workers |
| Qadah, T. et al (2023). Assessment of knowledge and attitude among healthcare workers towards monkeypox disease: a cross-sectional study from Saudi Arabia Italian Journal of Medicine, 17(2), 1645 | Saudi Arabia | Obs | No | NR | 7. Social sciences | Adults, Healthcare workers |
| Raccagni, A. R. et al (2023). Viral blips and virologic failures following mpox vaccination with MVA-BN among people with HIV AIDS, 37(15), 2365-2369 | Italy | Obs | No | NR | 3. Prophylaxis | Adults, PLHIV |
| Raccagni, A. R. et al (2023). Late positivization of oropharyngeal, plasma, anal, semen, and urine specimens which tested negative at the time of mpox diagnosis Clinical Microbiology and Infection, 29(8), 1096-1097 | Italy | Obs | No | NR | 4. Diagnostics | Adults, MSM |
| Rajkhowa, P. et al (2023). Factors Influencing Monkeypox Vaccination: A Cue to Policy Implementation Journal of Epidemiology and Global Health, 13(2), 226-238 | NR | Obs | No | NR | 7. Social sciences | NR |
| Raman, H. et al (2023). Knowledge of Medical Students Towards the Re-emergence of Human Monkeypox Virus Cureus, 15(10), e46761 | Malaysia | Obs | No | NR | 7. Social sciences | Adults, Students |
| Ramchandani, M. S. et al (2023). Effectiveness of the Modified Vaccinia Ankara Vaccine Against Mpox in Men Who Have Sex With Men: A Retrospective Cohort Analysis, Seattle, Washington Open Forum Infectious Diseases, 10(11), ofad528 | USA | Obs | Yes | NR | 3. Prophylaxis | Adults, MSM |
| Rawat, R. S. et al (2023). A Cross-Sectional Study To Assess Knowledge of Monkeypox Among Medical Students and Graduates in India Cureus, 15(11), e49744 | India | Obs | No | NR | 7. Social sciences | Adults, Students |
| Ren, F. et al (2023). Public awareness, specific knowledge, and worry about mpox (monkeypox): A preliminary community-based study in Shenzhen, China Frontiers in public health, 11, 1077564 | China | Obs | No | NR | 7. Social sciences | Adults |
| Ren, F. et al (2023). Willingness to receive and recommend hypothetical mpox vaccination and associated factors in Chinese adults: a community-based survey in Shenzhen, China Public Health, 225, 267-276 | China | Obs | No | NR | 7. Social sciences | Adults |
| Riad, A. et al (2022). Monkeypox Knowledge and Vaccine Hesitancy of Czech Healthcare Workers: A Health Belief Model (HBM)-Based Study Vaccines, 10(12), 2022 | Czech Republic | Obs | No | NR | 7. Social sciences | Adults, Healthcare workers |
| Riad, A. et al (2023). Monkeypox (mpox)-related knowledge and vaccine hesitancy among czech healthcare professionals Population Medicine, 5, 571-571 | Czech Republic | Obs | No | NR | 7. Social sciences | Adults, Healthcare workers |
| Riad, A. et al (2023). Belarusian Healthcare Professionals' Views on Monkeypox and Vaccine Hesitancy Vaccines, 11(8), 1368 | Belarus | Obs | No | NR | 7. Social sciences | Adults, Healthcare workers |
| Ricco, M. et al (2022). When a Neglected Tropical Disease Goes Global: Knowledge, Attitudes and Practices of Italian Physicians towards Monkeypox, Preliminary Results Tropical Medicine and Infectious Disease, 7(7), 135 | Italy | Obs | No | NR | 7. Social sciences | Adults, Healthcare workers |
| Rony, M. K. K. et al (2023). Knowledge and Attitude Regarding Human Monkeypox Virus Infection among Nurses: A Cross-Sectional Study SAGE Open Nursing, 9, | Bangladesh | Obs | No | NR | 7. Social sciences | Adults, Healthcare workers |
| Rosen, J. B. et al (2024). JYNNEOSTM effectiveness as post-exposure prophylaxis against mpox: Challenges using real-world outbreak data Vaccine, 42(3), 548-555 | USA | Obs | Yes | NR | 3. Prophylaxis | Adults, Older adults, Case contacts |
| Rosenberg, E. S. et al (2023). Effectiveness of JYNNEOS Vaccine Against Diagnosed Mpox Infection - New York, 2022 MMWR Morb Mortal Wkly Rep, 72(20), 559-563 | USA | Obs | Yes | NR | 3. Prophylaxis | Adults, Older adults |
| Rubio-Muniz, C. A. et al (2023). Monkeypox: is topical cidofovir a good idea? Clinical and Experimental Dermatology, 48(2), 132-134 | Spain | Int | No | NR | 5. Therapeutics | Adults, MSM |
| Sahin, T. K. et al (2023). Knowledge and Attitudes of Turkish Physicians towards Human Monkeypox Disease and Related Vaccination: A Cross-Sectional Study Vaccines, 11(1), 19 | Turkey | Obs | No | NR | 7. Social sciences | Adults, Healthcare workers |
| Saleh, A. I. et al (2023). Human monkeypox diagnose (HMD) strategy based on data mining and artificial intelligence techniques Computers in Biology and Medicine, 152, | Egypt | Obs | No | NR | 4. Diagnostics | NR |
| Salih, T. et al (2023). Demographic Disparities in Mpox Vaccination Series Completion, by Route of Vaccine Administration - California, August 9, 2022-March 31, 2023 MMWR. Morbidity and mortality weekly report, 72(30), 827-832 | USA | Obs | No | NR | 7. Social sciences | Adults, Older adults |
| Sallam, M. et al (2022). Assessing Healthcare Workers’ Knowledge and Their Confidence in the Diagnosis and Management of Human Monkeypox: A Cross-Sectional Study in a Middle Eastern Country Healthcare (Switzerland), 10(9), | Jordan | Obs | No | NR | 7. Social sciences | Adults, Healthcare workers |
| Sallam, M. et al (2022). Knowledge of Human Monkeypox and Its Relation to Conspiracy Beliefs among Students in Jordanian Health Schools: Filling the Knowledge Gap on Emerging Zoonotic Viruses Medicina (Kaunas), 58(7), | Jordan | Obs | No | NR | 7. Social sciences | Adults, Students |
| Sallam, M. et al (2022). Conspiratorial Attitude of the General Public in Jordan towards Emerging Virus Infections: A Cross-Sectional Study Amid the 2022 Monkeypox Outbreak Tropical Medicine and Infectious Disease, 7(12), 411 | Jordan | Obs | No | NR | 7. Social sciences | Adults |
| Sammartino, J. C. et al (2023). Characterization of immune response against monkeypox virus in cohorts of infected patients, historic and newly vaccinated subjects Journal of Medical Virology, 95(5), e28778 | Italy | Obs | No | NR | 3. Prophylaxis | Adults, Healthcare workers |
| Santos, G. R. S. et al (2024). Chemsex among men who have sex with men during the Mpox health crisis in Brazil: A nationwide web survey Public health nursing (Boston, Mass.), | Brazil | Obs | No | NR | 7. Social sciences | Adults, MSM |
| Scarinci, S. et al (2023). Evaluation of Smallpox Vaccination Coverage and Attitude towards Monkeypox Vaccination among Healthcare Workers in an Italian University Hospital Vaccines, 11(12), 1741 | Italy | Obs | No | NR | 7. Social sciences | Adults, Healthcare workers |
| Schildhauer, S. et al (2023). Reduced Odds of Mpox-Associated Hospitalization Among Persons Who Received JYNNEOS Vaccine - California, May 2022-May 2023 MMWR Morb Mortal Wkly Rep, 72(36), 992-996 | USA | Obs | Yes | NR | 3. Prophylaxis | Adults |
| Schubert, S. L. et al (2024). Understanding the Impact of Mpox on Sexual Health Clinical Services: A National Knowledge, Attitudes, and Practices Survey - United States, 2022 Sexually Transmitted Diseases, 51(1), 38-46 | USA | Obs | No | NR | 7. Social sciences | Adults, Healthcare workers |
| Seitkamal, K. et al (2023). Proof of principle for a sensitive, real time and label-free detection of poxviruses using optical fiber biosensors Optik, 288, | Kazakhstan | Obs | No | NR | 4. Diagnostics | NR |
| Shafei, A. M. et al (2023). Resident Physicians' Knowledge and Preparedness Regarding Human Monkeypox: A Cross-Sectional Study from Saudi Arabia Pathogens, 12(7), 872 | Saudi Arabia | Obs | No | NR | 7. Social sciences | Adults, Healthcare workers |
| Shan, H. et al (2023). Educational Intervention on the Knowledge of Monkeypox Virus among Undergraduate Students in Rawalpindi; a Single Institution Quasi-Experimental Study Journal of University Medical and Dental College, 14(2), 584-589 | Pakistan | Int | No | NR | 7. Social sciences | Adults, Students |
| Sharff, K. A. et al (2023). Cardiac events following JYNNEOS vaccination for prevention of Mpox Vaccine, 41(22), 3410-3412 | USA | Obs | No | NR | 3. Prophylaxis | Children (5-13 years), Adults, Older adults |
| Sharma, S. K. et al (2023). An Observational Study of Indian Medical Students: Are We Truly Aware of Monkeypox? Cureus, 15(8), e43952 | India | Obs | No | NR | 7. Social sciences | Adults, Students |
| Shean, R. C. et al (2024). Repeat testing of mpox specimens with late CTs improves detection of potential false positive cases Journal of Clinical Microbiology, 62(2), | NR | Obs | No | NR | 4. Diagnostics | Adults |
| Siegenbeek van Heukelom, M. L. et al (2023). Characteristics of mpox positive, versus mpox negative, and mpox unsuspected clients from the Centre of Sexual Health, Public Health Service of Amsterdam, 20 May to 15 September 2022 J Eur Acad Dermatol Venereol, 37(9), 1891-1896 | Netherlands | Obs | No | NR | 4. Diagnostics | Adults, MSM, PLHIV |
| Sistere-Oro, M. et al (2024). Pan-pox-specific T-cell responses in HIV-1-infected individuals after JYNNEOS vaccination Journal of Medical Virology, 96(1), e29317 | NR | Obs | No | NR | 3. Prophylaxis | Adults, PLHIV |
| Sklenovská, N. et al (2023). Design and validation of a laboratory-developed diagnostic assay for monkeypox virus Virus Genes, 59(6), 795-800 | Belgium | Int | Yes | I, II | 4. Diagnostics | NR |
| Smith, L. E. et al (2023). Did mpox knowledge, attitudes and beliefs affect intended behaviour in the general population and men who are gay, bisexual and who have sex with men? An online cross-sectional survey in the UK BMJ Open, 13(10), bmjopen-2022-070882 | UK | Obs | No | NR | 7. Social sciences | Adults, Older adults, MSM |
| Sobaikhi, N. H. et al (2023). Health Workers’ Knowledge and Attitude towards Monkeypox in Southwestern Saudi Arabia: A Cross-Sectional Study Diseases, 11(2), | Saudi Arabia | Obs | No | NR | 7. Social sciences | Adults, Healthcare workers |
| Sobral-Costas, T. G. et al (2023). Human monkeypox outbreak: Epidemiological data and therapeutic potential of topical cidofovir in a prospective cohort study Journal of the American Academy of Dermatology, 88(5), 1074-1082 | Spain | Int | Yes | NR | 5. Therapeutics | Adults, MSM |
| Soelaeman, R. H. et al (2022). Characteristics of JYNNEOS Vaccine Recipients Before and During a Large Multiday LGBTQIA+ Festival - Louisiana, August 9-September 5, 2022 MMWR. Morbidity and mortality weekly report, 71(43), 1379-1381 | USA | Obs | No | NR | 7. Social sciences | NR |
| Sorayaie Azar, A. et al (2023). Monkeypox detection using deep neural networks BMC Infect Dis, 23(1), 438 | NR | Obs | No | NR | 4. Diagnostics | NR |
| Specchiarello, E. et al (2023). Development and validation of a nanoplate-based digital PCR assay for absolute MPXV quantification J Virol Methods, 321, 114802 | Italy | Obs | No | NR | 4. Diagnostics | Adults, PLHIV |
| Starks, T. J. et al (2023). Correlates of Mpox Vaccination among Sexual Minority Men in the United States: Sexual Behavior, Substance Use, and Main Partner Relationships Journal of sex research, 60(5), 634-644 | USA | Obs | No | NR | 7. Social sciences | Adults, MSM |
| Stefanie, S. et al (2024). T Cell Responses against Orthopoxviruses in HIV-Positive Patients Vaccines, 12(2), 131 | Germany | Int | No | NR | 3. Prophylaxis | Adults, PLHIV |
| Svartstein, A. S. W. et al (2023). Mpox Incidence and Vaccine Uptake in Men Who Have Sex with Men and Are Living with HIV in Denmark Vaccines, 11(7), 1167 | Denmark | Obs | No | NR | 7. Social sciences | Adults, MSM, MSM |
| Swed, S. et al (2023). Monkeypox in Syria: Highlighting an awareness issue IJID Regions, 7, 271-276 | Syria | Obs | No | NR | 7. Social sciences | Adults, Healthcare workers, Students |
| Swed, S. et al (2023). A multinational cross-sectional study on the awareness and concerns of healthcare providers toward monkeypox and the promotion of the monkeypox vaccination Frontiers in public health, 11, 1153136.  Countries: Algeria, Egypt, Iraq, Jordan, Libya, Palestine, Saudi Arabia, Sudan, Syria, Tunisia, Yemen | see left | Obs | No | NR | 7. Social sciences | Adults, Healthcare workers |
| Swed, S. et al (2023). Monkeypox Post-COVID-19: Knowledge, Worrying, and Vaccine Adoption in the Arabic General Population Vaccines, 11(4), 759 | Algeria, Iraq, Qatar, Sudan, Syria, Yemen | Obs | No | NR | 7. Social sciences | Adults |
| Swed, S. et al (2023). Knowledge of mpox and its determinants among the healthcare personnel in Arabic regions: A multi-country cross-sectional study New Microbes and New Infections, 54, 101146.  Countries: Algeria, Egypt, Iraq, Jordan, Kuwait, Lebanon, Libya, Palestine, Qatar, Saudi Arabia, Somalia, Sudan, Syria, United Arab Emirates, Yemen | see left | Obs | No | NR | 7. Social sciences | Adults, Healthcare workers, Students |
| Swift, M. D. et al (2023). Early Experience With an Occupational JYNNEOS (Orthopoxvirus) Vaccination Program Journal of Occupational and Environmental Medicine, 65(6), 477-480 | USA | Int | No | NR | 7. Social sciences | Adults, Healthcare workers |
| Taha, T. Y. et al (2023). Design and Optimization of a Monkeypox virus Specific Serological Assay Pathogens, 12(3), | Democratic Republic of the Congo, Ghana | Obs | No | NR | 4. Diagnostics | NR |
| Tempestilli, M. et al (2024). Pharmacokinetics of tecovirimat in subjects with Mpox International Journal of Antimicrobial Agents, 63(2), 107068 | Italy | Int | No | NR | 5. Therapeutics | Adults, PLHIV |
| Tempestilli, M. et al (2023). Tecovirimat concentrations and viral suppression in seminal fluid from patients with mpox The Lancet Infectious Diseases, 23(5), 531-532 | Italy | Int | Yes | NR | 5. Therapeutics | Adults |
| Temsah, M. H. et al (2022). Monkeypox Disease (MPOX) Perceptions among Healthcare Workers versus General Population during the First Month of the WHO Alert: Cross-Sectional Survey in Saudi Arabia Vaccines, 10(12), 2071 | Saudi Arabia | Obs | No | NR | 7. Social sciences | Adults, Older adults, Healthcare workers |
| Temsah, M. H. et al (2022). Monkeypox caused less worry than COVID-19 among the general population during the first month of the WHO Monkeypox alert: Experience from Saudi Arabia Travel Medicine and Infectious Disease, 49, 102426 | Saudi Arabia | Obs | No | NR | 7. Social sciences | Adults, Older adults |
| Theban, A. A. et al (2024). Knowledge and attitude regarding monkeypox among Saudi MOH primary healthcare nurses in Jeddah: A cross-sectional study J Family Med Prim Care, 13(1), 175-181 | Saudi Arabia | Obs | No | NR | 7. Social sciences | Adults, Healthcare workers |
| Therrien, C. et al (2023). Development and validation of a highly specific in-house chemiluminescent-based serological assay for the detection of antibodies directed against the human monkeypox virus Journal of Virological Methods, 322, 114836 | Canada | Obs | No | NR | 4. Diagnostics | Adults, MSM |
| Thieme, A. H. et al (2023). A deep-learning algorithm to classify skin lesions from mpox virus infection Nature Medicine, 29(3), 738-747 | NR | Obs | Yes | NR | 4. Diagnostics | NR |
| Thomas, M. et al (2023). Knowledge and Attitude Regarding Human Monkeypox Infection Among Health Care Professionals: A Cross-Sectional Study in A Tertiary Care Hospital in Northern Uttar Pradesh International Journal of Pharmaceutical and Clinical Research, 15(6), 869-880 | India | Obs | No | NR | 7. Social sciences | Adults, Healthcare workers |
| Thy, M. et al (2022). Breakthrough Infections after Postexposure Vaccination against Mpox New England Journal of Medicine, 387(26), 2477-2479 | France | Obs | No | NR | 3. Prophylaxis | NR |
| Titanji, B. K. et al (2023). Effectiveness of Smallpox Vaccination to Prevent Mpox in Military Personnel New England Journal of Medicine, 389(12), 1147-1148 | USA | Obs | Yes | NR | 3. Prophylaxis | Adults, Older adults, Military personnel |
| Tomita, N. et al (2023). An open-label, non-randomized study investigating the safety and efficacy of smallpox vaccine, LC16, as post-exposure prophylaxis for mpox Human Vaccines and Immunotherapeutics, 19(2), 2242219 | Japan | Int | No | NR | 3. Prophylaxis | NR |
| Torres, T. S. et al (2023). Evaluation of Mpox Knowledge, Stigma, and Willingness to Vaccinate for Mpox: Cross-Sectional Web-Based Survey Among Sexual and Gender Minorities JMIR public health and surveillance, 9, e46489 | Brazil | Obs | No | NR | 7. Social sciences | Adults |
| Tran, P. T. et al (2023). Knowledge, attitude, and practice of medical students on human Monkeypox in Southern Vietnam Journal of Medical Pharmaceutical and Allied Sciences, 12(6), 6164-6169 | Vietnam | Obs | No | NR | 7. Social sciences | Adults, Students |
| Turner Overton, E. et al (2023). A randomized phase 3 trial to assess the immunogenicity and safety of 3 consecutively produced lots of freeze-dried MVA-BN vaccine in healthy adults Vaccine, 41(2), 397-406 | NR | Int | No | NR | 3. Prophylaxis | Adults |
| Turpin, R. E. et al (2023). Monkeypox-Related Stigma and Vaccine Challenges as a Barrier to HIV Pre-Exposure Prophylaxis among Black Sexual Minority Men International Journal of Environmental Research and Public Health, 20(14), 6324 | USA | Obs | No | NR | 7. Social sciences | Adults, MSM, Black sexual minority men |
| Tweed, M. et al (2023). Delivering a community-based monkeypox vaccination programme in partnership International Journal of STD and AIDS, 34(6), 427-429 | UK | Obs | No | NR | 7. Social sciences | Adults, MSM |
| Ubals, M. et al (2023). Evaluating the Accuracy of Self-Collected Swabs for the Diagnosis of Mpox Clinical Infectious Diseases, 76(7), 1311-1314 | Spain | Obs | No | NR | 4. Diagnostics | Adults |
| Ugwu, S. et al (2023). Knowledge, attitude, and perception of monkeypox among medical/health students across media space in Nigeria Population Medicine, 5, | Nigeria | Obs | No | NR | 7. Social sciences | Adults, Students, Other are <18 but range not specified |
| Uhteg, K. et al (2023). Validation and implementation of an orthopoxvirus qualitative real-time PCR for the diagnosis of monkeypox in the clinical laboratory Journal of Clinical Virology, 158, 105327 | USA | Obs | No | NR | 4. Diagnostics | NR |
| Uzun Ozsahin, D. et al (2023). Computer-Aided Detection and Classification of Monkeypox and Chickenpox Lesion in Human Subjects Using Deep Learning Framework Diagnostics, 13(2), 292 | NR | Obs | Yes | NR | 4. Diagnostics | NR |
| van der Boom, M. et al (2023). Adverse reactions following MPox (monkeypox) vaccination: An overview from the Dutch and global adverse event reporting systems British Journal of Clinical Pharmacology, 89(11), 3302-3310 | Netherlands | Obs | No | NR | 3. Prophylaxis | Infants (<12 months old), Young Children (1-5 years), Children (5-13 years), Adolescents, Adults, Older adults |
| van Ewijk, C. E. et al (2023). Acceptance and timeliness of post-exposure vaccination against mpox in high-risk contacts, Amsterdam, the Netherlands, May-July 2022 Vaccine, 41(47), 6952-6959 | Netherlands | Obs | Yes | NR | 3. Prophylaxis | Children (5-13 years), Adolescents, Adults, Older adults |
| Vellappally, S. et al (2024). Knowledge of the monkeypox 2022 outbreak among dental hygienists and students in Saudi Arabia: A cross-sectional study International journal of dental hygiene, 22(1), 187-193 | Saudi Arabia | Obs | No | NR | 7. Social sciences | Adults, Healthcare workers, Students |
| Velu, P. D. et al (2023). Evaluation of a Zoonotic Orthopoxvirus PCR Assay for the Detection of Mpox Virus Infection Journal of Molecular Diagnostics, 25(10), 740-747 | USA | Obs | Yes | I, II | 4. Diagnostics | Infants (<12 months old), Young Children (1-5 years), Children (5-13 years), Adolescents, Adults |
| Villa, R. D. et al (2024). A Laboratory-Developed Assay for Clade II Human Mpox Virus on the Panther Fusion Open Access System Journal of Infectious Diseases, 229(Supplement 2), S132-S136 | USA | Obs | No | II | 4. Diagnostics | NR |
| Vo, C. et al (2023). Clinical Characteristics and Outcomes of Patients With Mpox Who Received Tecovirimat in a New York City Health System Open Forum Infectious Diseases, 10(11), ofad552 | USA | Obs | No | NR | 5. Therapeutics | Adults, PLHIV |
| Von Tokarski, F. et al (2023). Smallpox vaccine acceptability among French men having sex with men living with HIV in settings of monkeypox outbreak AIDS, 37(5), 855-856 | France | Obs | No | NR | 7. Social sciences | Adults, MSM, PLHIV |
| Walsh-Buhi, E. R. et al (2024). Mpox knowledge in the U.S.: Results from a nationally representative survey Journal of Infection and Public Health, 17(2), 359-361 | USA | Obs | No | NR | 7. Social sciences | Adults |
| Walsh-Buhi, M. L. et al (2024). A theory-based assessment of mpox: Findings from a nationally representative survey of U.S. adults PLoS ONE, 19(3 MARCH), e0299599 | USA | Obs | No | NR | 7. Social sciences | Adults |
| Wang, B. et al (2023). Perceptions, precautions, and vaccine acceptance related to monkeypox in the public in China: A cross-sectional survey Journal of Infection and Public Health, 16(2), 163-170 | China | Obs | No | NR | 7. Social sciences | Adults |
| Wang, C. et al (2023). Colorimetric–fluorescent dual-signal enhancement immunochromatographic assay based on molybdenum disulfide-supported quantum dot nanosheets for the point-of-care testing of monkeypox virus Chemical Engineering Journal, 472, | NR | Obs | No | NR | 4. Diagnostics | NR |
| Wang, H. et al (2022). Monkeypox self-diagnosis abilities, determinants of vaccination and self-isolation intention after diagnosis among MSM, the Netherlands, July 2022 Eurosurveillance, 27(33), | Netherlands | Obs | No | NR | 7. Social sciences | Adults, MSM, NR |
| Wang, H. et al (2022). Perceived Monkeypox Concern and Risk among Men Who Have Sex with Men: Evidence and Perspectives from The Netherlands Tropical Medicine and Infectious Disease, 7(10), 293 | Netherlands | Obs | No | NR | 7. Social sciences | Adults, MSM, PLHIV |
| Wang, H. et al (2024). Brief Report: Determinants of Potential Sexual Activity Reduction in the Face of the Mpox Epidemic International journal of behavioral medicine, | Netherlands | Obs | No | NR | 7. Social sciences | Adults, MSM, PLHIV, Students |
| Wang, H. et al (2023). Monkeypox Knowledge Investigation and Knowledge Gap Analysis: An Online Cross-Sectional Survey in China Risk Management and Healthcare Policy, 16, 2741-2756 | China | Obs | No | NR | 7. Social sciences | Adults, Veterinarians/Farmers/Animal workers, Students |
| Wang, L. et al (2023). Rapid and ultrasensitive detection of mpox virus using CRISPR/Cas12b-empowered graphene field-effect transistors Applied Physics Reviews, 10(3), | NR | Int | No | NR | 4. Diagnostics | NR |
| Wang, Y. et al (2024). Ultrasensitive one-pot detection of monkeypox virus with RPA and CRISPR in a sucrose-aided multiphase aqueous system Microbiology spectrum, 12(1), e0226723 | China | Int | No | NR | 4. Diagnostics | NR |
| Wei, J. et al (2023). MASTR Pouch: Palm-size lab for point-of-care detection of Mpox using recombinase polymerase amplification and CRISPR technology Sensors and Actuators B: Chemical, 390, | China | Int | Yes | NR | 4. Diagnostics | NR |
| Wettengel, J. M. et al (2023). Implementation and clinical evaluation of an Mpox virus laboratory-developed test on a fully automated random-access platform Journal of Medical Virology, 95(8), e29022 | Germany | Obs | No | NR | 4. Diagnostics | NR |
| Wilber, E. et al (2023). Utility of a Viral Vesicular Panel Multiplex Polymerase Chain Reaction Assay for the Diagnosis of Monkeypox, Herpes Simplex, and Varicella Zoster Viruses Open Forum Infectious Diseases, 10(3), ofad140 | NR | Obs | No | NR | 4. Diagnostics | Adults, Healthcare workers |
| Winters, M. et al (2022). Attitudes towards Monkeypox vaccination and predictors of vaccination intentions among the US general public PLoS ONE, 17(12 December), e0278622 | USA | Obs | No | NR | 7. Social sciences | NR |
| Witzel, T. C. et al (2024). Experiences of mpox illness and case management among cis and trans gay, bisexual and other men who have sex with men in England: a qualitative study eClinicalMedicine, 102522 | UK | Obs | No | NR | 7. Social sciences | Adults, MSM |
| Wolff Sagy, Y. et al (2023). Real-world effectiveness of a single dose of mpox vaccine in males Nature Medicine, 29(3), 748-752 | Israel | Obs | No | NR | 3. Prophylaxis | Adults, PLHIV |
| Wong, N. S. et al (2023). Mpox vaccination for men who have sex with men and their differential risk of exposure and infection Human Vaccines and Immunotherapeutics, 19(2), 2252263 | China | Obs | No | NR | 7. Social sciences | Adults, MSM |
| Xia, A. et al (2023). Cross-reactive antibody response to Monkeypox virus surface proteins in a small proportion of individuals with and without Chinese smallpox vaccination history BMC Biol, 21(1), 205 | China | Obs | Yes | IIb | 3. Prophylaxis | Adults |
| Xiao, F. et al (2024). Loop-mediated isothermal amplification coupled with nanoparticle-based lateral flow biosensor for monkeypox virus detection Talanta, 269, 125502 | China | Int | No | I, II | 4. Diagnostics | NR |
| Xu, Y. et al (2024). Behavioral intentions of self-isolation and informing close contacts after developing mpox-related symptoms among young men who have sex with men in China Journal of Medical Virology, 96(2), e29470 | China | Obs | No | NR | 7. Social sciences | Adults, MSM |
| Yan, H. et al (2024). A rapid and sensitive fluorescent chromatography with cloud system for MPXV point-of-care diagnosis Analytica Chimica Acta, 1302, | China | Obs | No | NR | 4. Diagnostics | NR |
| Yang, J. Z. (2024). Comparative risk perception of the monkeypox outbreak and the monkeypox vaccine Risk analysis: an official publication of the Society for Risk Analysis et al (2024). Comparative risk perception of the monkeypox outbreak and the monkeypox vaccine Risk analysis: an official publication of the Society for Risk Analysis, 44(2), 295-303 | USA | Obs | No | NR | 7. Social sciences | NR |
| Yang, J. et al (2023). Development of highly accurate digital PCR method and reference material for monkeypox virus detection Analytical and bioanalytical chemistry, 415(7), 1333-1337 | NR | Obs | No | NR | 4. Diagnostics | NR |
| Yang, X. et al (2023). Development of a CRISPR/Cas12a-recombinase polymerase amplification assay for visual and highly specific identification of the Congo Basin and West African strains of mpox virus Journal of Medical Virology, 95(5), e28757 | China | Obs | No | II | 4. Diagnostics | NR |
| Yang, Y. et al (2024). Mpox knowledge and vaccination hesitancy among healthcare workers in Beijing, China: A cross-sectional survey Vaccine: X, 16, 100434 | China | Obs | No | NR | 7. Social sciences | Adults, Healthcare workers |
| Yates, J. L. et al (2023). Development of a novel serological assay for the detection of mpox infection in vaccinated populations Journal of Medical Virology, 95(10), e29134 | USA | Int | Yes | NR | 4. Diagnostics | Adults |
| Youssef, D. et al (2023). When a neglected tropical zoonotic disease emerges in non-endemic countries: need to proactively fill the unveiled knowledge gaps towards human monkeypox among the Lebanese population Journal of Pharmaceutical Policy and Practice, 16(1), 39 | Lebanon | Obs | No | NR | 7. Social sciences | Adults, Healthcare workers |
| Youssef, D. et al (2023). Following the COVID-19 playbook and battling another infodemic: conspiracy beliefs around human monkeypox among the Lebanese population Journal of Pharmaceutical Policy and Practice, 16(1), 72 | Lebanon | Obs | No | NR | 7. Social sciences | Adults |
| Yu, C. et al (2023). Development of a Novel Loop-Mediated Isothermal Amplification Method for the Rapid Detection of Monkeypox Virus Infections Viruses, 15(1), 84 | NR | Obs | No | I, IIa, IIb | 4. Diagnostics | NR |
| Yu, L. et al (2024). Evaluation of monkeypox knowledge and attitudes among Chinese medical students BMC public health, 24(1), 745 | China | Obs | No | NR | 7. Social sciences | Adults, Students |
| Yu, Q. et al (2023). Molybdenum disulfide-loaded multilayer AuNPs with colorimetric-SERS dual-signal enhancement activities for flexible immunochromatographic diagnosis of monkeypox virus Journal of Hazardous Materials, 459, 132136 | China | Obs | No | NR | 4. Diagnostics | NR |
| Yupari-Azabache, I. L. et al (2023). Validity and Reliability of the Knowledge, Attitudes and Practices Instrument Regarding Monkey Pox in Peru Risk Manag Healthc Policy, 16, 1509-1520 | Peru | Obs | No | NR | 7. Social sciences | Adults |
| Zaeck, L. M. et al (2023). Low levels of monkeypox virus-neutralizing antibodies after MVA-BN vaccination in healthy individuals Nature Medicine, 29(1), 270-278 | Netherlands | Obs | No | IIb | 3. Prophylaxis | Older adults (>65 years) |
| Zatla, I. et al (2024). ASSESSING THE KNOWLEDGE OF SMALLPOX AND MONKEYPOX VIRUS AMONG THE UNIVERSITY OF TLEMCEN MEMBERS IN THE WAKE OF COVID-19: A 2023 CROSS-SECTIONAL STUDY African Journal of Infectious Diseases, 18(1), 5-10 | Algeria | Obs | No | NR | 7. Social sciences | Adults, Older adults |
| Zeidan, R. K. et al (2023). Assessment of Knowledge, Perceptions, and Attitudes During the Global Mpox Outbreak in June 2022: A Cross-Sectional Study From the United Arab Emirates International journal of public health, 68, 1606080 | United Arab Emirates | Obs | No | NR | 7. Social sciences | Adults |
| Zenone, M. et al (2022). Using Data from a Short Video Social Media Platform to Identify Emergent Monkeypox Conspiracy Theories JAMA Network Open, E2236993 | NR | Obs | No | NR | 7. Social sciences | NR |
| Zhang, L. et al (2024). A high-throughput DNA analysis method based on isothermal amplification on a suspension microarray for detecting mpox virus and viruses with comparable symptoms Analytica Chimica Acta, 1299, 342416 | China | Int | No | NR | 4. Diagnostics | NR |
| Zhang, W. et al (2024). Mpox patients' experience from infection to treatment and implications for prevention and control: A multicenter qualitative study in China Journal of Medical Virology, 96(1), e29338 | China | Obs | No | NR | 7. Social sciences | Adults, In-hospital patients |
| Zhang, Z. et al (2023). Rapid Detection of the Monkeypox Virus Genome and Antigen Proteins Based on Surface-Enhanced Raman Spectroscopy ACS applied materials & interfaces, 15(29), 34419-34426 | NR | Int | No | NR | 4. Diagnostics | NR |
| Zhao, F. et al (2024). A field diagnostic method for rapid and sensitive detection of mpox virus Journal of Medical Virology, 96(2), e29469 | China | Obs | No | NR | 4. Diagnostics | NR |
| Zhen, Z. et al (2023). Cross-reactive antibodies against monkeypox virus exist in the population immunized with vaccinia Tian Tan strain in China Infection, Genetics and Evolution, 113, 105477 | China | Obs | Yes | NR | 3. Prophylaxis | Young Children (1-5 years), Children (5-13 years), Adults |
| Zheng, M. et al (2023). Awareness of mpox-related knowledge among men who have sex with men in China BMC Public Health, 23(1), 600 | China | Obs | No | NR | 7. Social sciences | Adults, MSM |
| Zheng, M. et al (2023). Lower rate of mpox vaccination hesitancy and medical consultation among Chinese men who have sex with men living with HIV in comparison with those living without HIV: A national observational study Human Vaccines and Immunotherapeutics, 19(3), 2290788 | China | Obs | No | NR | 7. Social sciences | Adults, PLHIV |
| Zheng, M. et al (2023). Mpox Vaccination Hesitancy and Its Associated Factors among Men Who Have Sex with Men in China: A National Observational Study Vaccines, 11(9), 1432 | China | Obs | No | NR | 7. Social sciences | Adults, MSM |
| Zheng, M. et al (2022). Knowledge and vaccination acceptance toward the human monkeypox among men who have sex with men in China Frontiers in Public Health, 10, | China | Obs | No | NR | 7. Social sciences | Adults, MSM |
| Zhou, H. C. et al (2023). Awareness, Attitude, and Knowledge Among the Healthcare Workers in China at the Onset of the Oversea Monkeypox Outbreak J Gen Intern Med, 38(13), 2914-2920 | China | Obs | No | NR | 7. Social sciences | Adults, Healthcare workers |
| Zhou, J. et al (2023). Rapid detection of monkeypox virus by multiple cross displacement amplification combined with nanoparticle-based biosensor platform Journal of Medical Virology, 95(2), e28479 | China | Obs | No | II | 4. Diagnostics | NR |
| Zimmermann, H. M. L. et al (2023). Mpox stigma among men who have sex with men in the Netherlands: Underlying beliefs and comparisons across other commonly stigmatized infections Journal of Medical Virology, 95(9), e29091 | Netherlands | Obs | No | NR | 7. Social sciences | Adults, MSM, PLHIV |
| Zucker, R. et al (2023). Examining the Patterns of Mpox Vaccine Uptake in a Vulnerable Population Sexually Transmitted Diseases, 50(10), 680-684 | Israel | Obs | No | NR | 7. Social sciences | Adults, at risk group |
| Zucman, D. et al (2022). Monkeypox Vaccine Hesitancy in French Men Having Sex with Men with PrEP or Living with HIV in France Vaccines, 10(10), 1629 | France | Obs | No | NR | 7. Social sciences | Adults, MSM, PLHIV |

*Under Populations Adolescents:13-17 years old; Adults: 18-65 years old

Abbreviations: NR=not reported, USA= United States of America, UK=United Kingdom, Obs=observational, Int=interventional, MSM=men who have sex with men, PLHIV= people living with HIV, NR=not reported.
