# Supplementary material for: Gaps in the global health research landscape for mpox: an analysis of research activities and existing evidence
Source: BMC Med. 2025 Sep 29;23:522. doi: 10.1186/s12916-025-04350-1 (PMC12482760; doi:10.1186/s12916-025-04350-1)
Supplement: Supplementary file 7 — Additional file 7: Fig. S2 Evolution of mpox publications over time [file 12916_2025_4350_MOESM7_ESM.docx]

# **Additional file 7: Fig. S2**. Evolution of mpox publications over time

 
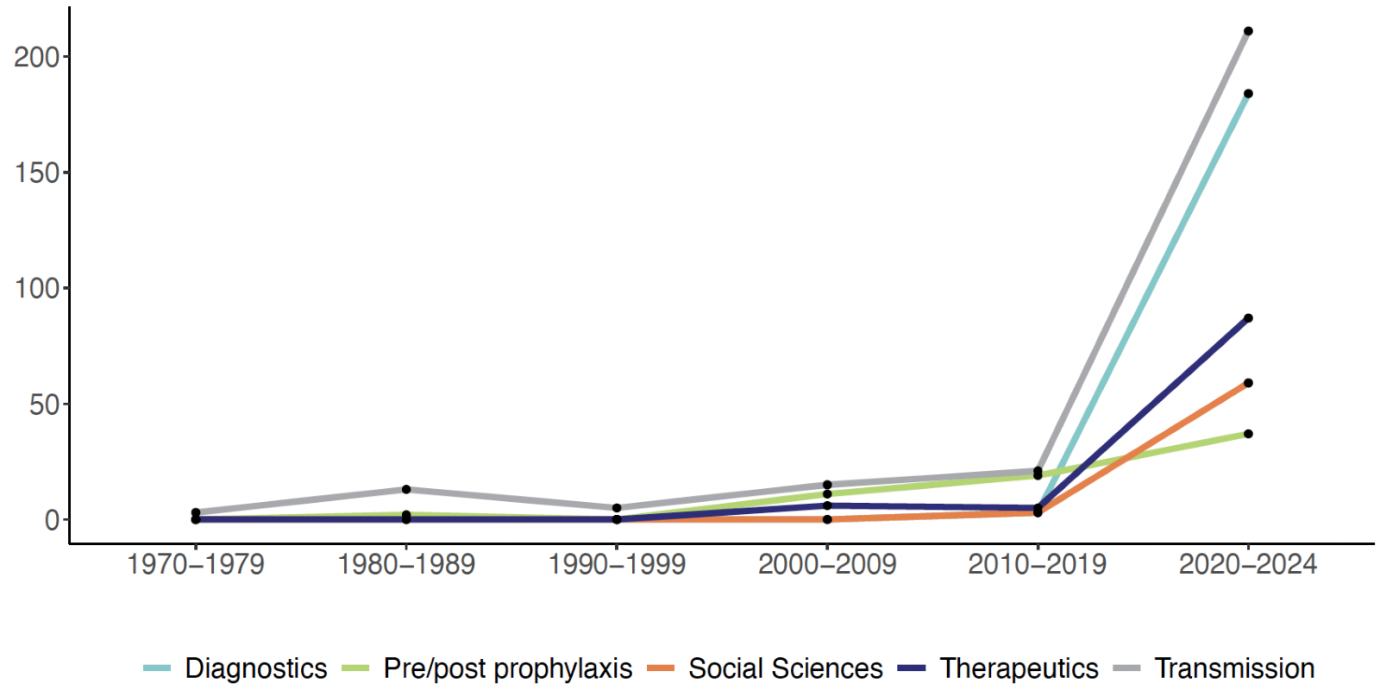


The figure presents the number of primary publications from systematic reviews in domains 2 to 7 by year of publication.
